# Supplementary material for: Functional and structural dissection of glycosyltransferases underlying the glycodiversity of wolfberry-derived bioactive ingredients lycibarbarspermidines
Source: Nat Commun. 2024 May 30;15:4588. doi: 10.1038/s41467-024-49010-9 (PMC11139883; doi:10.1038/s41467-024-49010-9)
Supplement: Supplementary file 1 — Supplementary Information [file 41467_2024_49010_MOESM1_ESM.pdf]

Supplementary Information

Table of Contents

Supplementary Figures **3**

Supplementary Figure 1. Chemical structure formula of lycibarbarspermidines. 3

Supplementary Figure 2. Phylogenetic analysis of LbUGTs with related functional glycosyltransferases from plants. 4

Supplementary Figure 3. HPLC profiles of LbUGT1-20 *in vitro* assays using **1** as the substrate. 5

Supplementary Figure 4. HPLC chromatography and MS spectra of the standards (**4**-**6**, **10**-**14** and **18**). 7

Supplementary Figure 5. Identification of LbUGT3 converting LS-B and LS-D into **15**. 8

Supplementary Figure 6. Effects of reaction temperature on enzyme activity of LbUGT1-5. 9

Supplementary Figure 7. Effects of divalent metal ions on enzyme activity of LbUGT1-5 10

Supplementary Figure 8. Determination of the kinetic parameters of LbUGT1-5. 11

Supplementary Figure 9. Sugar donor selectivity of LbUGT1-5. 12

Supplementary Figure 10. Identification of LbUGT1 transforming LS-D and LbUGT2 converting LS-A to generate Lyciamarspermidine C. 13

Supplementary Figure 11. Stereo view of the electron densities of the UDP in LbUGT1 (A) and UDP in LbUGT3 (B). 14

Supplementary Figure 12. Structural comparison of LbUGT1 and LbUGT3 with other homologs. 15

Supplementary Figure 13. Time evolutions of the two key distances of the binging modes. 16

Supplementary Figure 14. HPLC profiles of LbUGT1 *in vitro* assays using caffeic acid (A) and dihydrocaffeic acid (B) as the substrates. 17

Supplementary Figure 15. The binding conformations of LbUGT2 (A) and LbUGT4 (B). 18

Supplementary Figure 16. HPLC profiles of LbUGT1 *in vitro* assays using kukoamine B as the substrate. 19

Supplementary Figure 17. Di-glycosylation modes of multifunctional glycosyltransferases 20

Supplementary Figure 18. Amino acid sequence alignment of LbUGT1-5 with other plant-derived glycosyltransferases. 21

Supplementary Figure 19. HRESIMS and NMR spectra of **7**/**8**. 25

Supplementary Figure 20. HRESIMS and NMR spectra of **9**. 29

Supplementary Figure 21. HRESIMS and NMR spectra of **15**. 33

Supplementary Figure 22. HRESIMS and NMR spectra of **16**. 37

Supplementary Figure 23. HRESIMS and NMR spectra of **17**. 41

Supplementary Tables **42**

Supplementary Table 1. GenBank ID of UGTs used for phylogenetic analysis. 42

Supplementary Table 2. Identities and positives of LbUGT1-5 to each other. 43

Supplementary Table 3. Data collection and refinement statistics. 44

Supplementary Table 4. NMR assignments for **7** (^{1}H for 600 MHz and ^{13}C for 150 MHz in DMSO-d_6). 45

Supplementary Table 5. NMR assignments for **8** (^{1}H for 600 MHz and ^{13}C for 150 MHz in DMSO-d_6). 47

Supplementary Table 6. NMR assignments for **9** (^{1}H for 400 MHz and ^{13}C for 100 MHz in DMSO-d_6). 49

Supplementary Table 7. NMR assignments for **15** (^{1}H for 400 MHz and ^{13}C for 100 MHz in DMSO-d_6). 51

Supplementary Table 8. NMR assignments for **16** (^{1}H for 600 MHz and ^{13}C for 150 MHz in DMSO-d_6). 53

Supplementary Table 9. NMR assignments for **17** (^{1}H for 600 MHz and ^{13}C for 150 MHz in DMSO-d_6). 55

Supplementary Table 10. Primers used for constructing recombinant plasmids. 57

Supplementary Table 11. Newly predicted DNA and protein sequences involved in this study. 59

Supplementary Figures

A

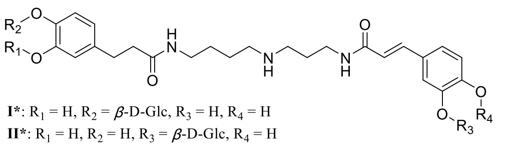

I*: R_1 = H, R_2 = β-D-Glc, R_3 = H, R_4 = H
II*: R_1 = H, R_2 = H, R_3 = β-D-Glc, R_4 = H
III: R_1 = β-D-Glc, R_2 = H, R_3 = H, R_4 = H
IV*: R_1 = H, R_2 = H, R_3 = H; R_4 = β-D-Glc
V*: R_1 = H, R_2 = β-D-Glc, R_3 = β-D-Glc, R_4 = H
VI: R_1 = β-D-Glc, R_2 = H, R_3 = β-D-Glc, R_4 = H
VII: R_1 = H, R_2 = β-D-Glc$^{\underline{4}}\beta$-D-Glc, R_3 = H, R_4 = β-D-Glc
VIII: R_1 =H , R_2 = β-D-Glc, R_3 = H, R_4 = β-D-Glc$^{\underline{4}}\beta$-D-Glc
IX: R_1 =H , R_2 = β-D-Glc, R_3 = H, R_4 = β-D-Glc$^{\underline{3}}\beta$-D-Glc
X: R_1 = H, R_2 = β-D-Glc, R_3 = β-D-Glc$^{\underline{3}}\beta$-D-Glc, R_4 = H
XI: R_1 = β-D-Glc, R_2 = H, R_3 = H; R_4 = β-D-Glc
XII*: R_1 = H, R_2 = β-D-Glc, R_3 = H, R_4 = β-D-Glc
XIII*: R_1 = H, R_2 =H, R_3 = H, R_4 =H

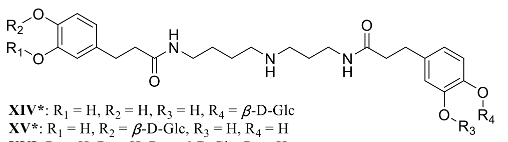

XIV*: R_1 = H, R_2 = H, R_3 = H, R_4 = β-D-Glc
XV*: R_1 = H, R_2 = β-D-Glc, R_3 = H, R_4 = H
XVI: R_1 = H, R_2 = H, R_3 = β-D-Glc, R_4 = H
XVII: R_1 = β-D-Glc, R_2 = H, R_3 = β-D-Glc, R_4 = H
XVII*: R_1 = H, R_2 = β-D-Glc, R_3 = H, R_4 = β-D-Glc
XIX: R_1 = β-D-Glc, R_2 = H, R_3 = H, R_4 = β-D-Glc
XX*: R_1 = H, R_2 =H, R_3 = H, R_4 =H

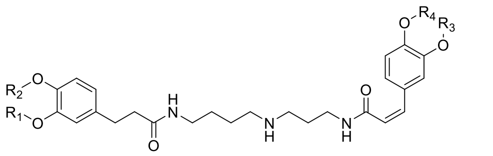

XXI: R_1 = H, R_2 = β-D-Glc, R_3 = β-D-Glc, R_4 = H
XXII: R_1 = H, R_2 = H, R_3 = β-D-Glc, R_4 = H
XXIII: R_1 = H, R_2 = H, R_3 = H, R_4 = β-D-Glc
XXIV: R_1 = H, R_2 = β-D-Glc, R_3 = H, R_4 = H
XXV: R_1 = H, R_2 = β-D-Glc, R_3 = H, R_4 = β-D-Glc

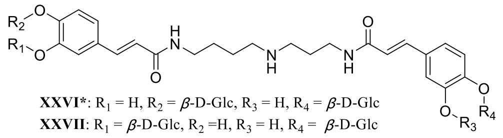

XXVI*: R_1 = H, R_2 = β-D-Glc, R_3 = H, R_4 = β-D-Glc
XXVII: R_1 = β-D-Glc, R_2 =H, R_3 = H, R_4 = β-D-Glc
XXVIII*: R_1 = H, R_2 =H, R_3 = H, R_4 =H

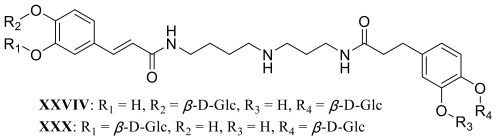

XXVIV: R_1 = H, R_2 = β-D-Glc, R_3 = H, R_4 = β-D-Glc
XXX: R_1 = β-D-Glc, R_2 = H, R_3 = H, R_4 = β-D-Glc

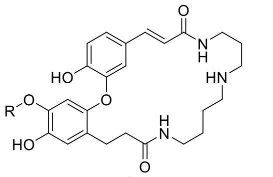

XXXI: R = β-D-Glc
XXXII: R = β-D-Glc $^{\underline{3}}\beta$-D-Glc

B

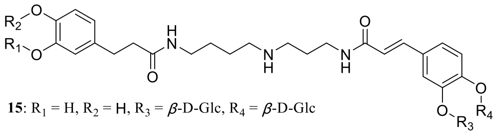

15: R_1 = H, R_2 = H, R_3 = β-D-Glc, R_4 = β-D-Glc

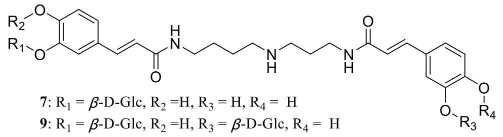

7: R_1 = β-D-Glc, R_2 =H, R_3 = H, R_4 = H
9: R_1 = β-D-Glc, R_2 =H, R_3 = β-D-Glc, R_4 = H
16: R_1 = β-D-Glc, R_2 =β-D-Glc, R_3 = H, R_4 = H
17: R_1 = H, R_2 =H, R_3 = β-D-Glc, R_4 = β-D-Glc

Supplementary Figure 1. Chemical structure formula of lycibarbarspermidines.

(A) Lycibarbarspermidines were isolated from wolfberry by our group. The asterisk-marked compounds were utilized in this study. (B) The new lycibarbarspermidines identified in this work.

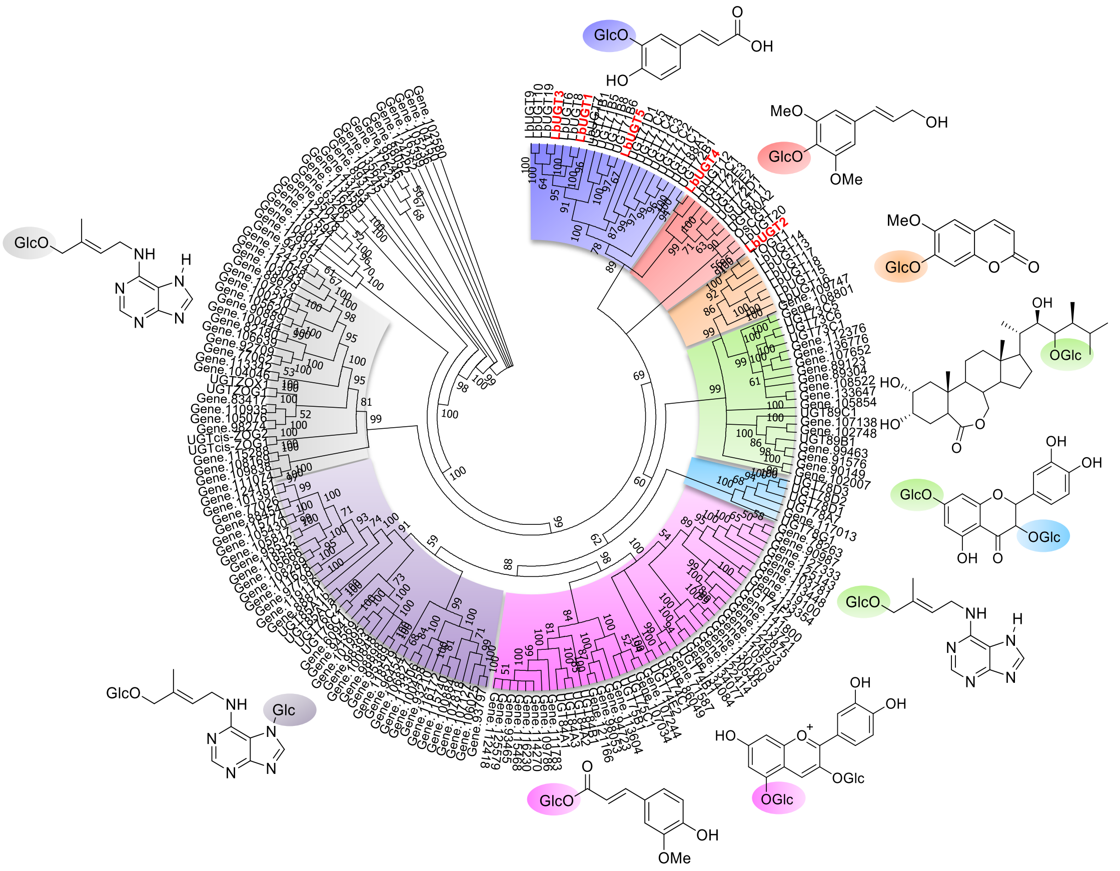

Supplementary Figure 2. Phylogenetic analysis of LbUGTs with related functional glycosyltransferases from plants.

LbUGT1-5 were marked by red font. The GenBank ID of the UGTs were listed in Supplementary Table **1**.

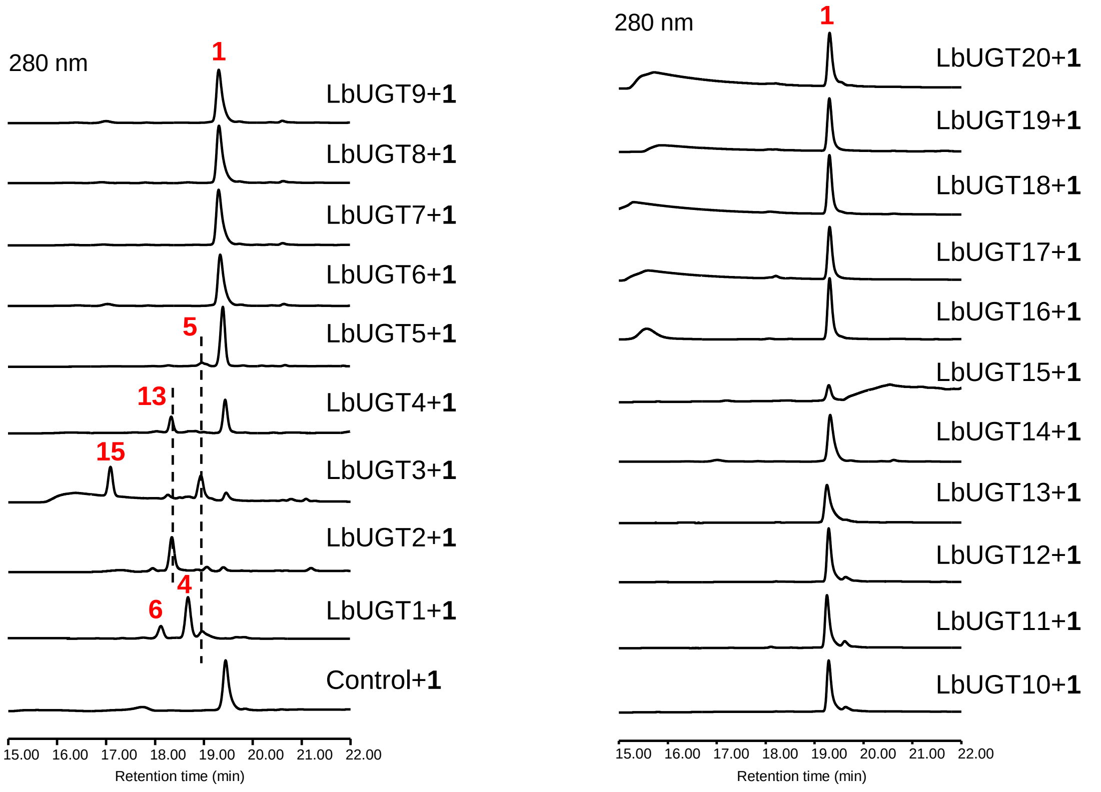

Supplementary Figure 3. HPLC profiles of LbUGT1-20 *in vitro* assays using 1 as the substrate.

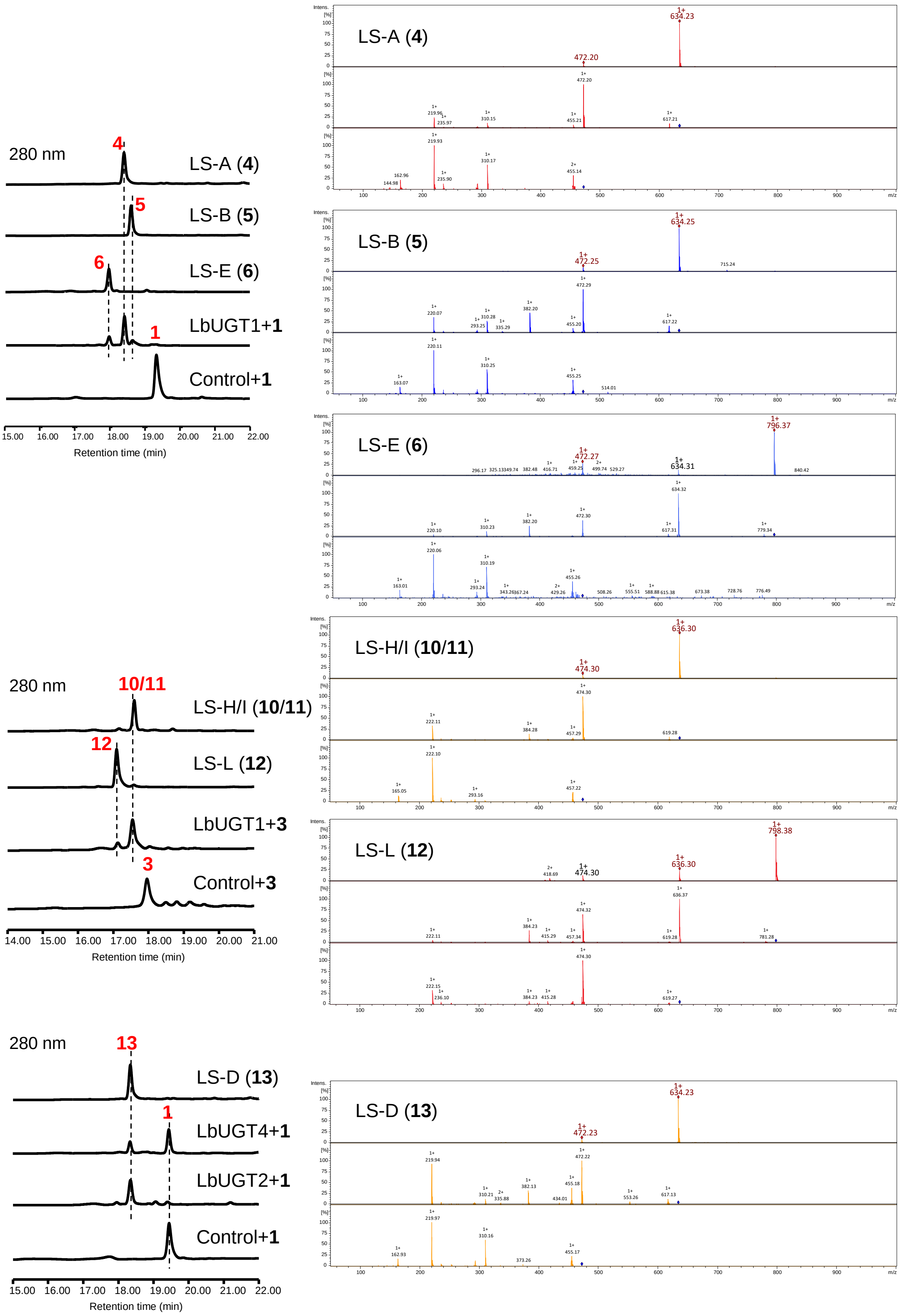
280 nm
LS-A (4)
LS-B (5)
LS-E (6)
LbUGT1+1
Control+1
Retention time (min)
LS-H/I (10/11)
LS-L (12)
LbUGT1+3
Control+3
LS-D (13)
LbUGT4+1
LbUGT2+1
Intens. [%]
m/z
634.23
472.20
634.25
472.25
796.37
472.27
634.31
636.30
474.30
798.38
472.23

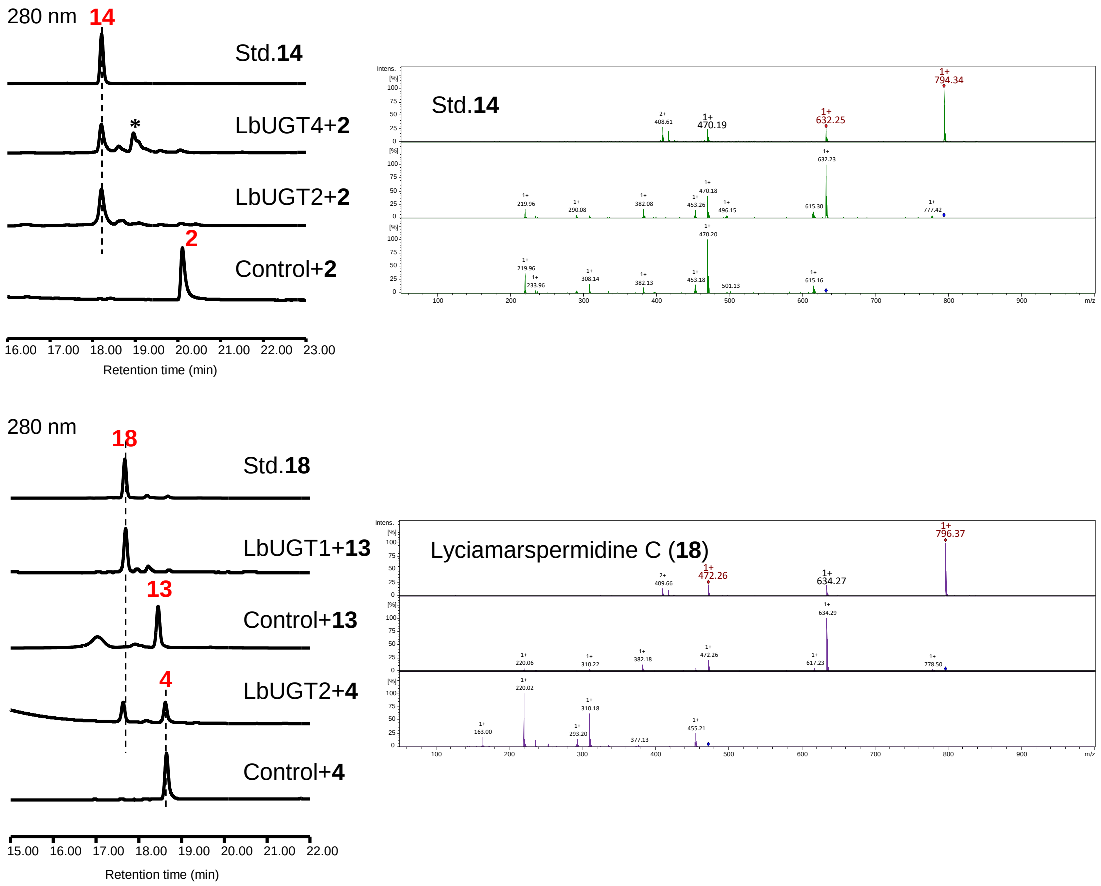

Supplementary Figure 4. HPLC chromatography and MS spectra of the standards (4-6, 10-14 and 18).

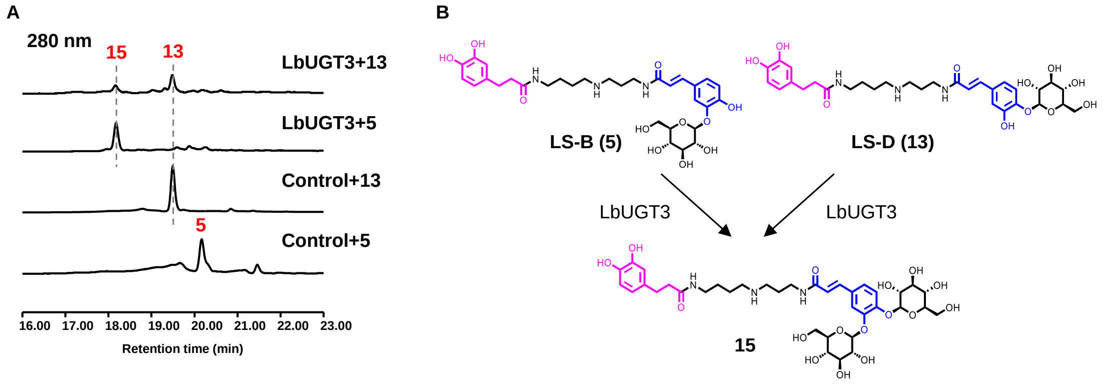

Supplementary Figure 5. Identification of LbUGT3 converting LS-B and LS-D into 15.

(A) HPLC profiles of LbUGT3 *in vitro* assays using **5** and **13** as the substrates. (B) Reactions catalyzed by LbUGT3.

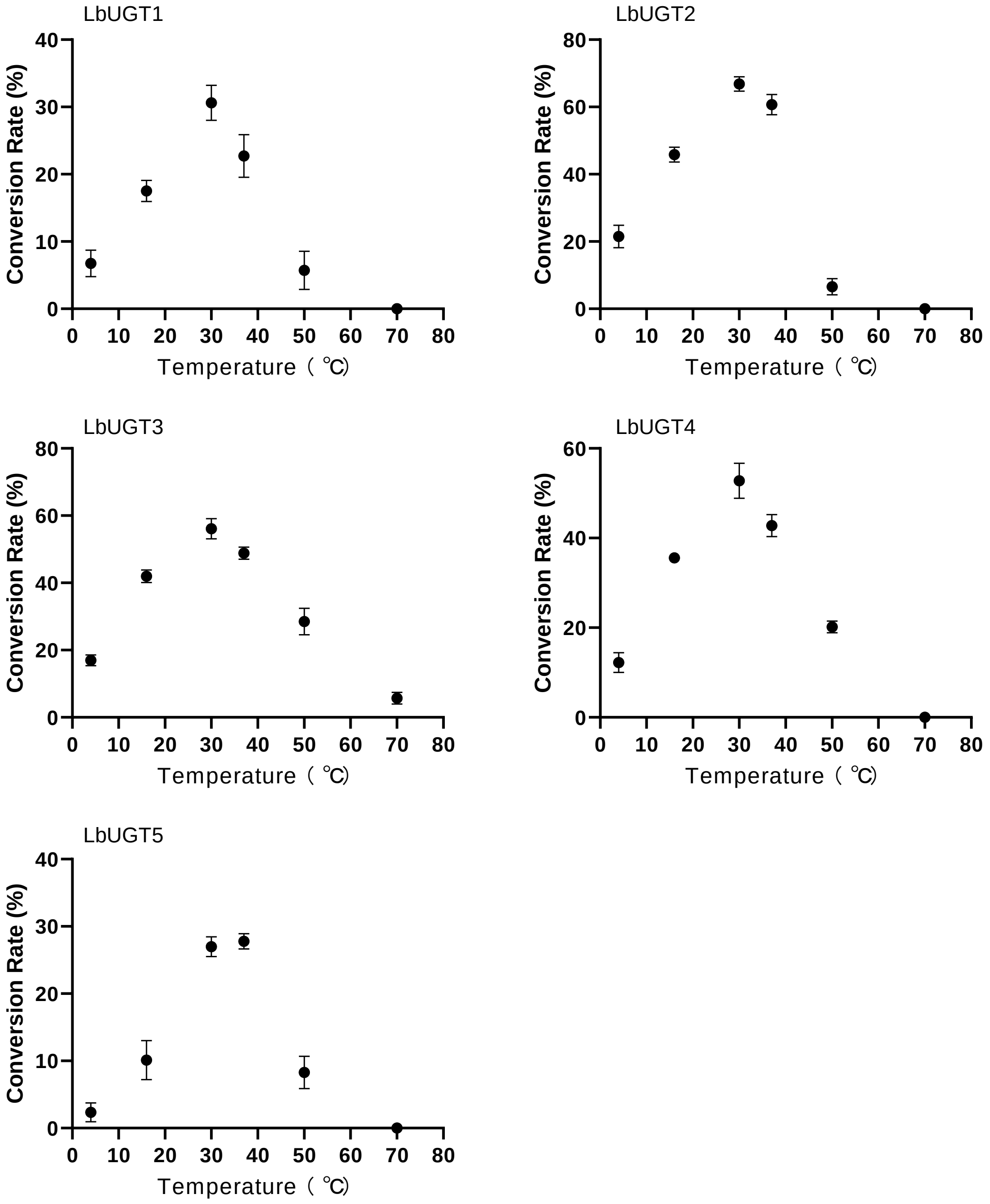

Supplementary Figure 6. Effects of reaction temperature on enzyme activity of LbUGT1-5.

1 was used as sugar acceptor for LbUGT1-4, **2** was used as substrate for LbUGT5 and the reaction mixtures were incubated with UDP-Glc at 30°C for 3 h. Source data are provided as a Source Data file.

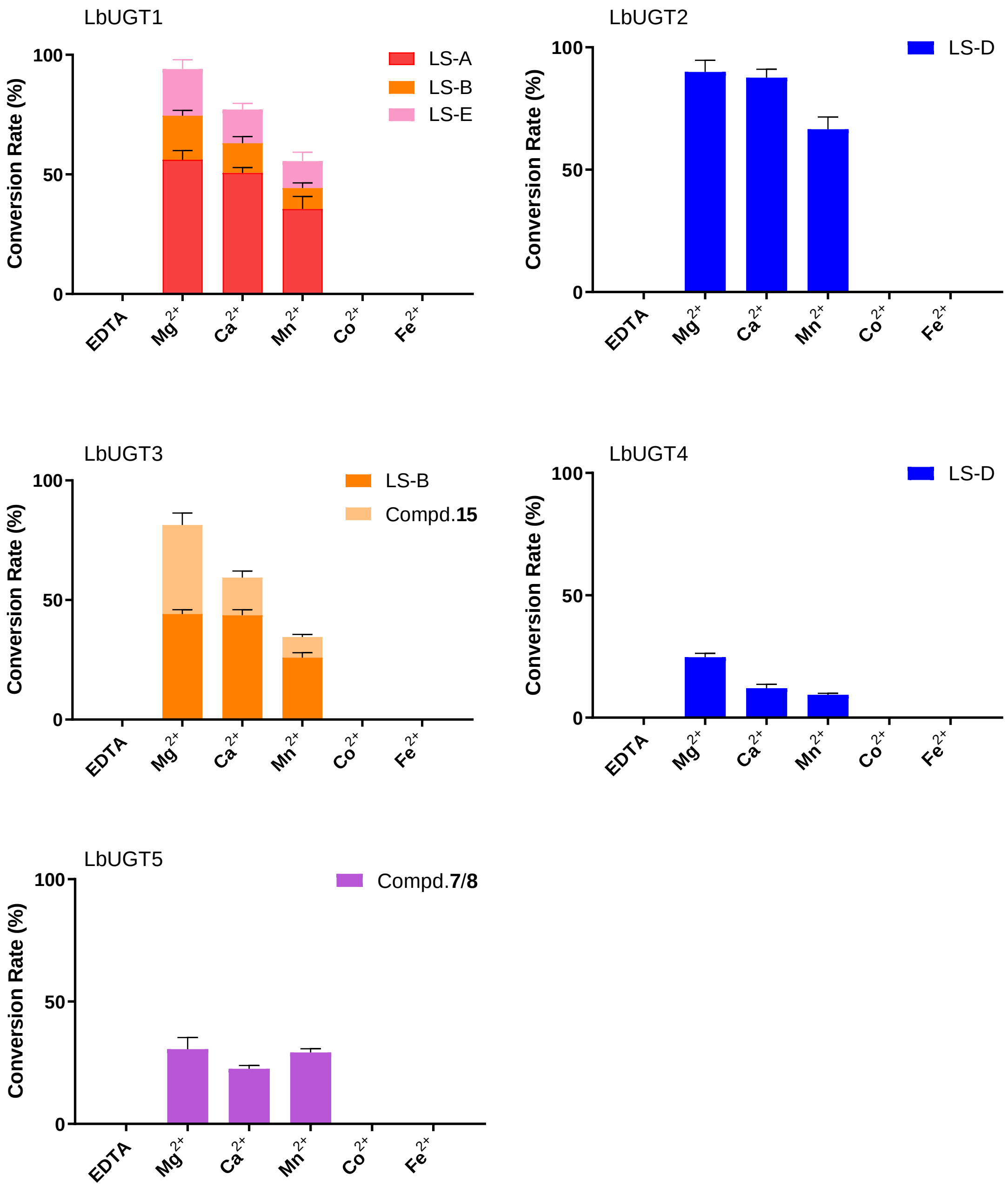

Supplementary Figure 7. Effects of divalent metal ions on enzyme activity of LbUGT1-5. **1** was used as sugar acceptor for LbUGT1-4, **2** was used as substrate for LbUGT5 and the reaction mixtures were incubated with UDP-Glc at 30°C for 12 h. Source data are provided as a Source Data file.

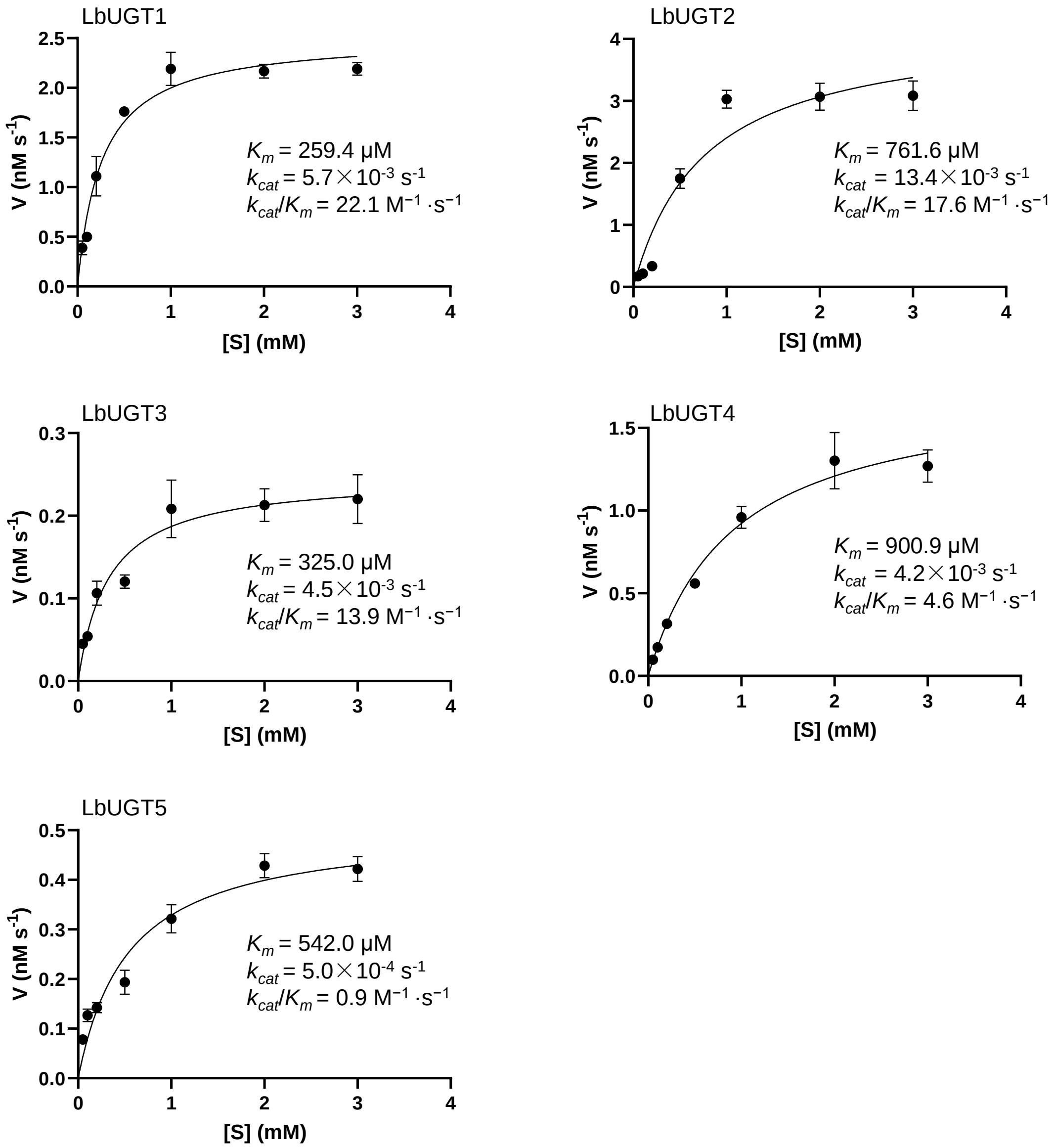

Supplementary Figure 8. Determination of the kinetic parameters of LbUGT1-5.

1 was used as sugar acceptor for LbUGT1-4, **2** was used as substrate for LbUGT5 and the reaction mixtures were incubated with UDP-Glc at 30°C for 1 h. Source data are provided as a Source Data file.

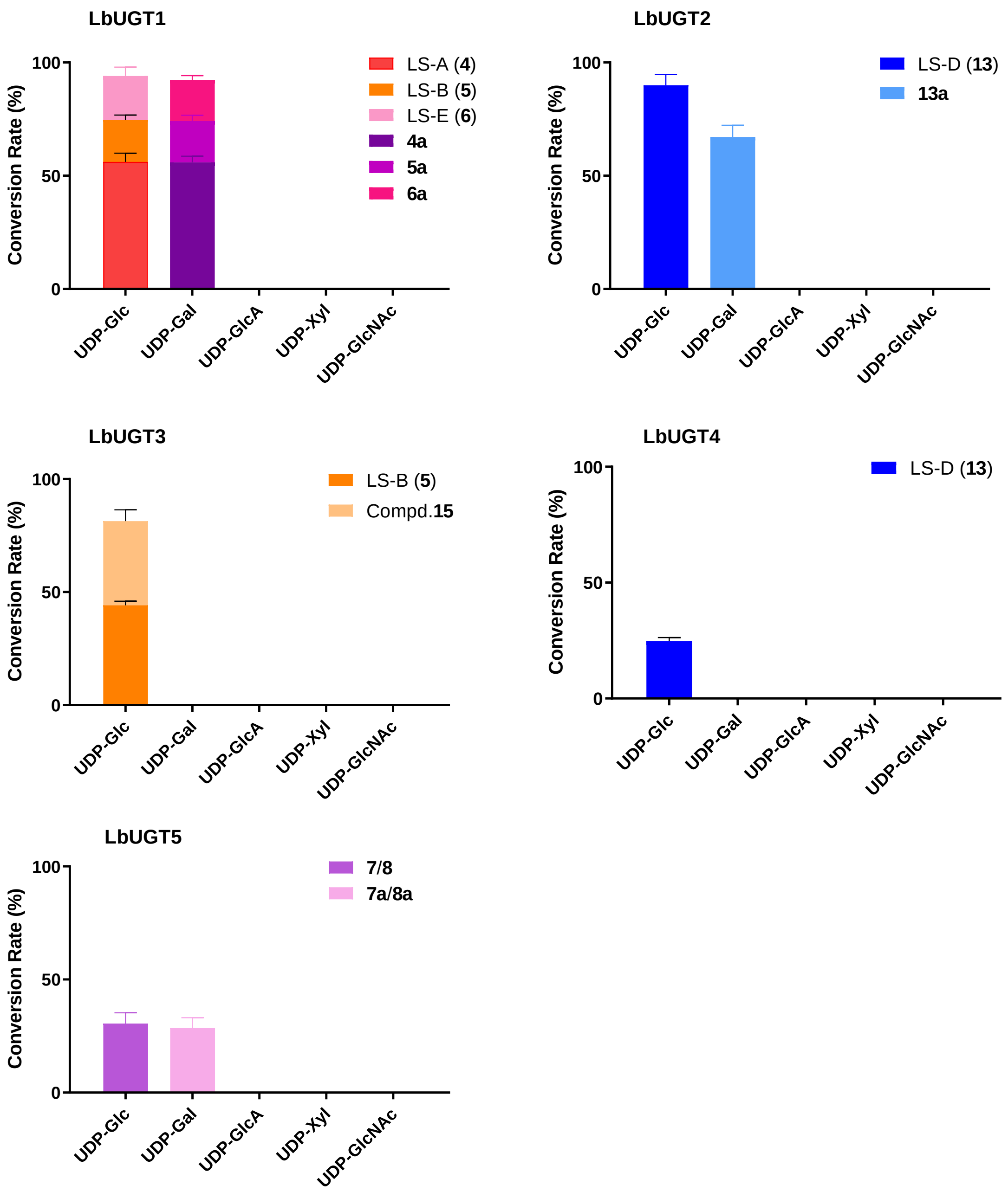

Supplementary Figure 9. Sugar donor selectivity of LbUGT1-5.

Conversion rates of LbUGT1-5 using five sugar donors, **1** was used as sugar acceptor for LbUGT1-4, **2** was used as substrate for LbUGT5 and the reaction mixtures were incubated with UDP-Glc at 30°C for 12 h. Source data are provided as a Source Data file.

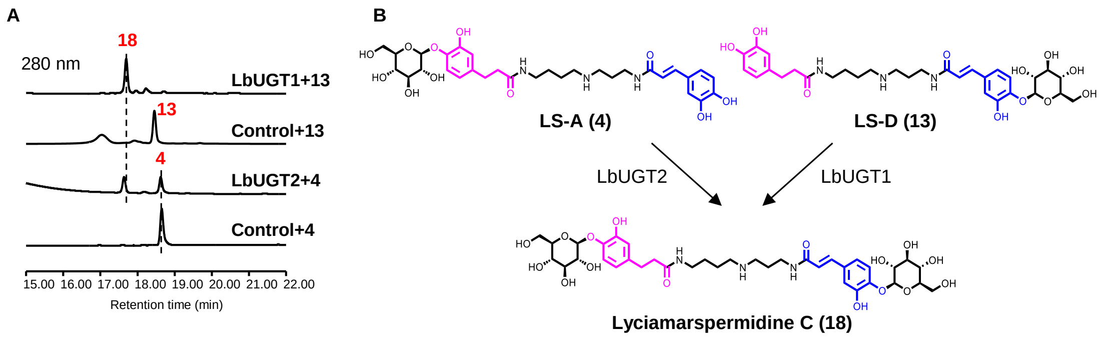

Supplementary Figure 10. Identification of LbUGT1 transforming LS-D and LbUGT2 converting LS-A to generate Lyciamarspermidine C.

(A) HPLC profiles of LbUGT1 and 2 *in vitro* assays using **13** or **4** as the substrates. (B) Reactions catalyzed by LbUGT1 and 2.

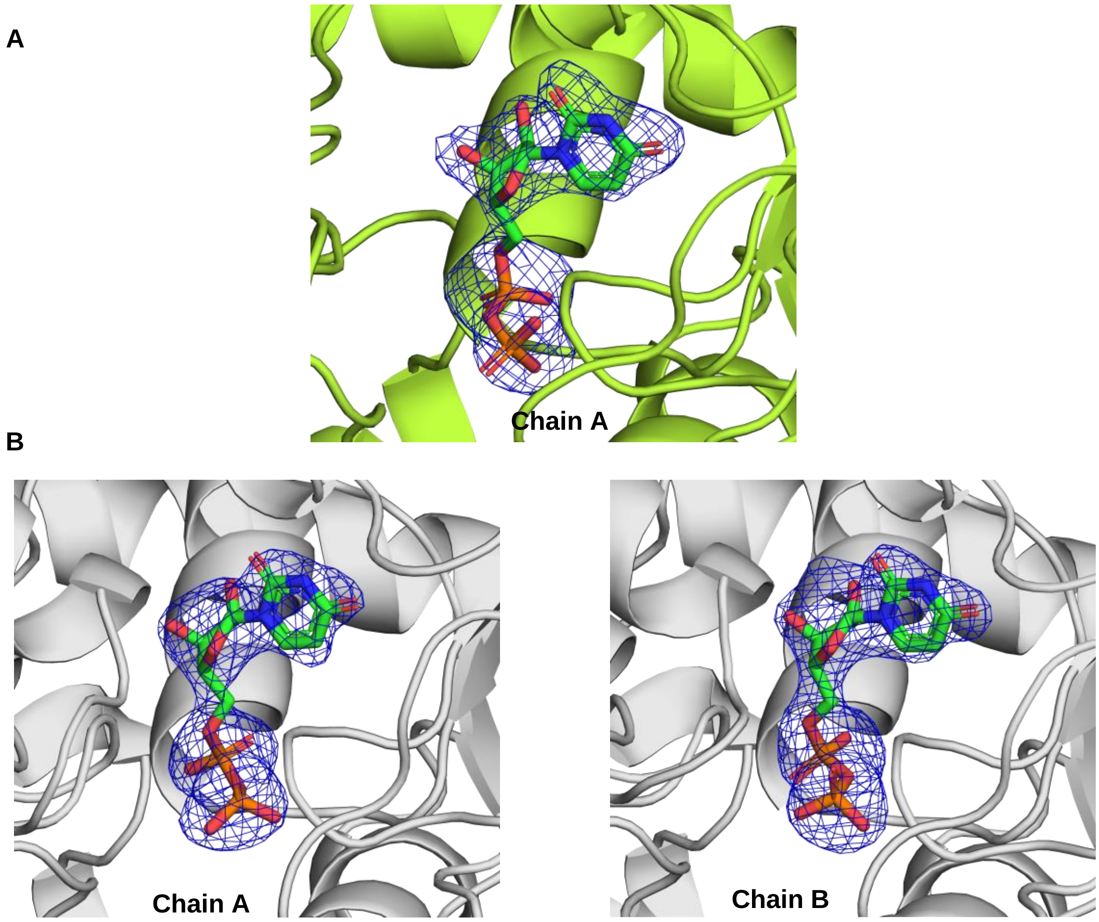

Supplementary Figure 11. Stereo view of the electron densities of the UDP in LbUGT1 (A) and UDP in LbUGT3 (B).

Simulated annealing omit map are shown in blue mesh and contoured at 3.0σ (A) and 2.0σ (B).

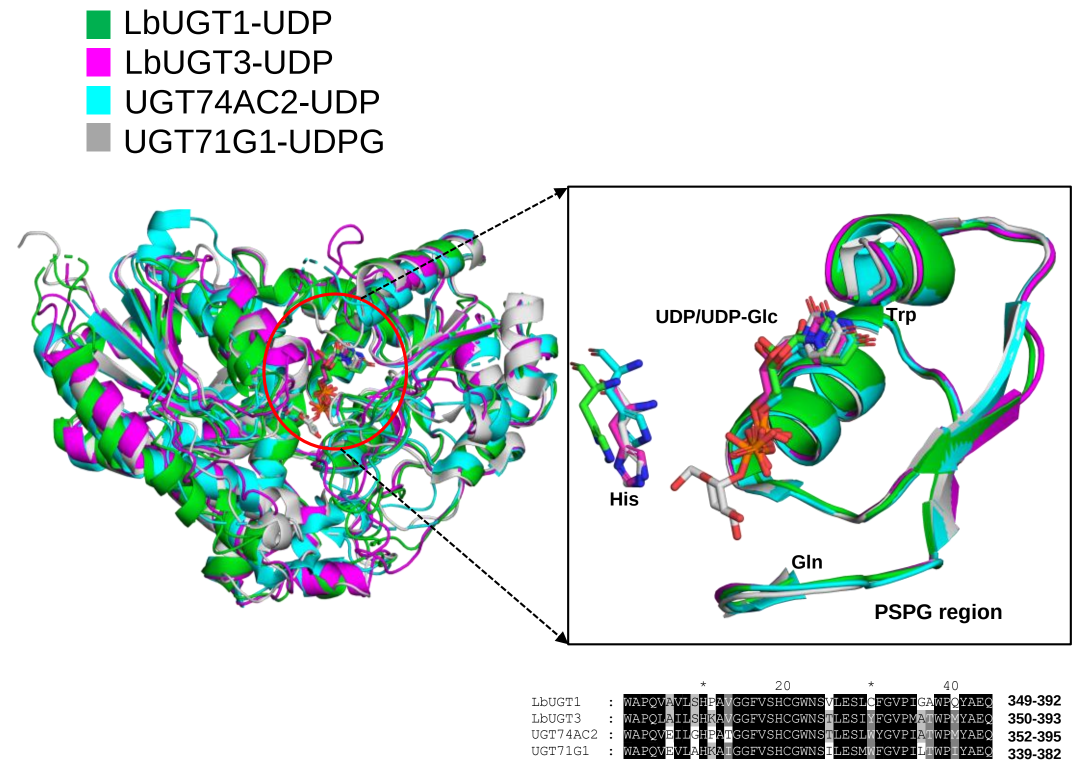

Supplementary Figure 12. Structural comparison of LbUGT1 and LbUGT3 with other homologs.

Superposition of LbUGT1 (green) and LbUGT3 (magenta) with UGT74AC2 (cyan, PDB ID: 7bv3) and UGT71G1 (gray, PDB ID: 2acw) identified by DALI search. The conserved catalytic residue His, PSPG regions and UDP moiety are highlighted. The sequence positions of the PSPG box are also shown below.

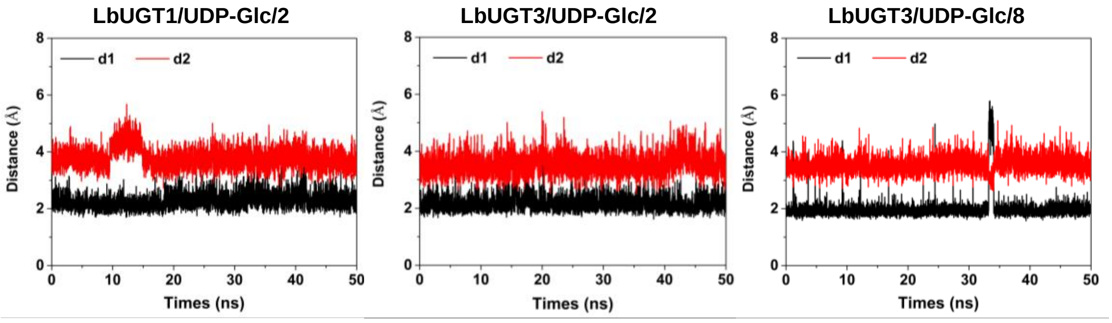

Supplementary Figure 13. Time evolutions of the two key distances of the binging modes. Time evolutions of the two key distances between the hydroxyl H atom of substrate and the N atom of the catalytic histidine (d1) and between the hydroxyl O atom of substrate and the acetal C atom of UDP-Glc (d2) in the 50 ns MD simulations of LbUGT1 and LbUGT3 binding modes.

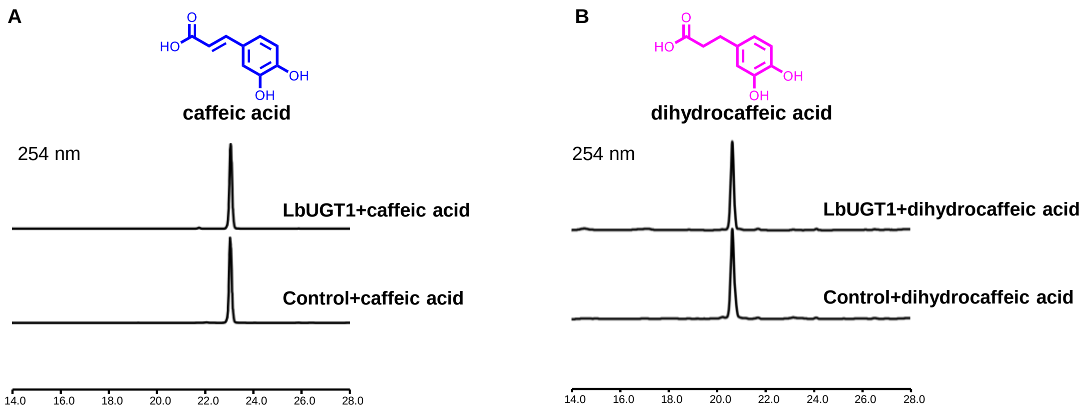

Supplementary Figure 14. HPLC profiles of LbUGT1 *in vitro* assays using caffeic acid (A) and dihydrocaffeic acid (B) as the substrates.

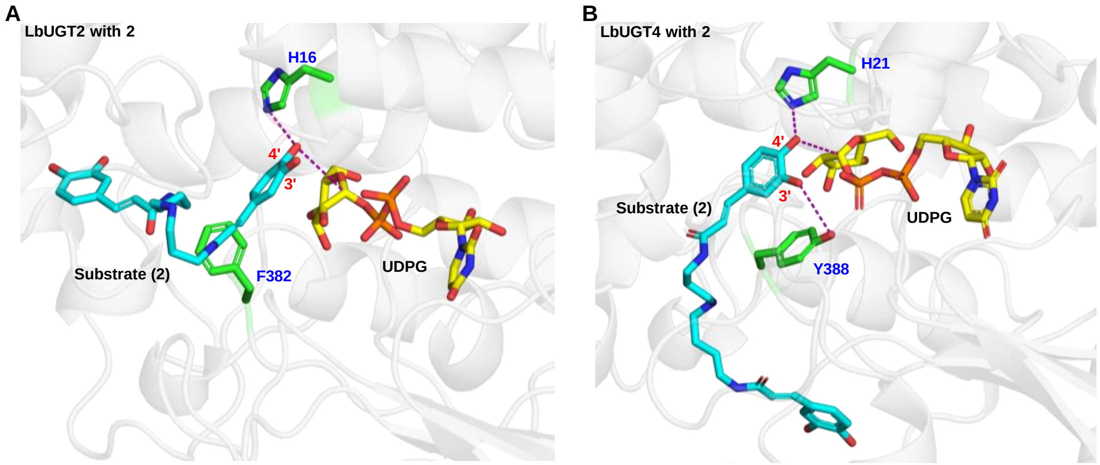

Supplementary Figure 15. The binding conformations of LbUGT2 (A) and LbUGT4 (B). The common substrate **2** shown in cyan and UDP-Glc shown in yellow. The key amino acids were labeled by green sticks and the hydrogen bonds were shown with the purple dash.

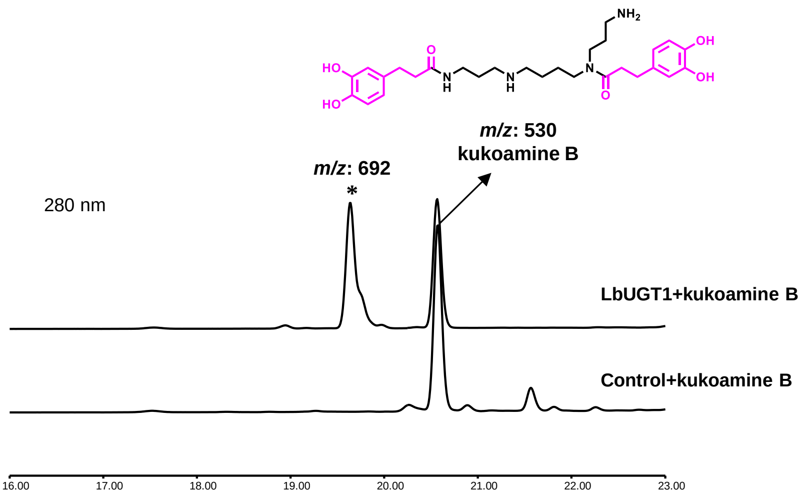

Supplementary Figure 16. HPLC profiles of LbUGT1 *in vitro* assays using kukoamine B as the substrate.

The peak marked with an asterisk is the presumed monoglycoside, as indicated by its molecular weight being 162 Da greater than that of kukoamine B.

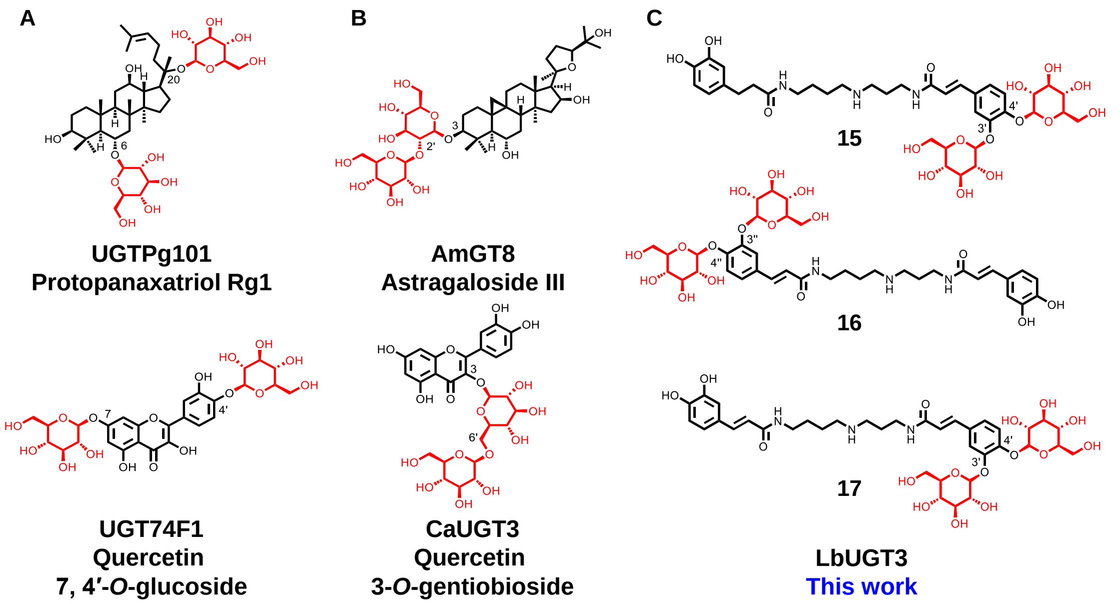

Supplementary Figure 17. Di-glycosylation modes of multifunctional glycosyltransferases. (A) The mode of di-glycosylation with distantly positioned glycosylation sites. (B) The mode of di-glycosylation through elongation of the glucose chain. (C) The di-glycosylation of the ortho-hydroxyl groups on the benzene ring.

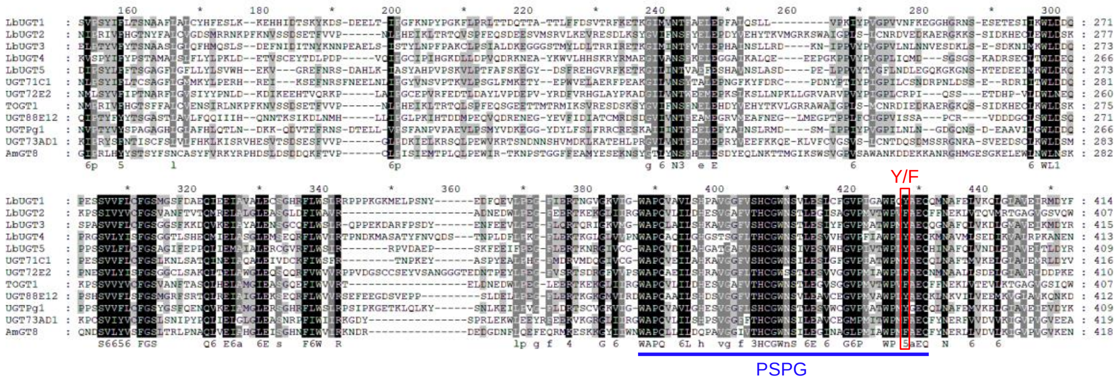

Supplementary Figure 18. Amino acid sequence alignment of LbUGT1-5 with other plant-derived glycosyltransferases.

A

Elemental Composition Report **Page 1**

Single Mass Analysis
Tolerance = 20.0 PPM / DBE: min = -1.5, max = 50.0
Element prediction: Off
Number of isotope peaks used for i-FIT = 2

Monoisotopic Mass, Even Electron Ions
244 formula(e) evaluated with 4 results within limits (all results (up to 1000) for each mass)
Elements Used:
C: 0-40 H: 0-60 N: 2-4 O: 0-20
106-469-4-6
20211122-60 44 (0.374) 1: TOF MS ES+ 1.84e+004

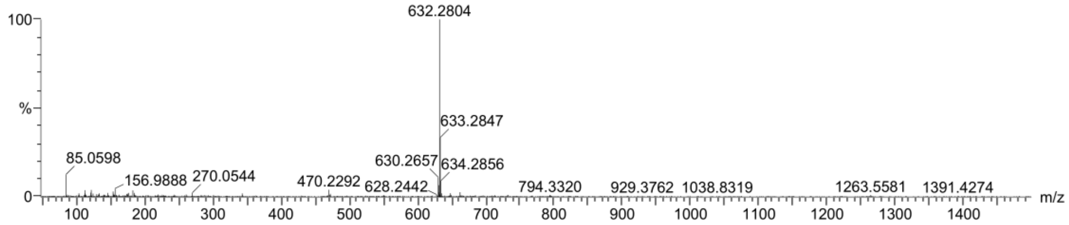

Minimum:				-1.5		
Maximum:		5.0	20.0	50.0		
Mass	Calc. Mass	mDa	PPM	DBE	Conf(%)	Formula
632.2804	632.2819	-1.5	-2.4	12.5	95.41	C31 H42 N3 O11
	632.2761	4.3	6.8	21.5	0.99	C38 H38 N3 O6
	632.2878	-7.4	-11.7	3.5	3.28	C24 H46 N3 O16
	632.2726	7.8	12.3	-0.5	0.32	C20 H46 N3 O19

B

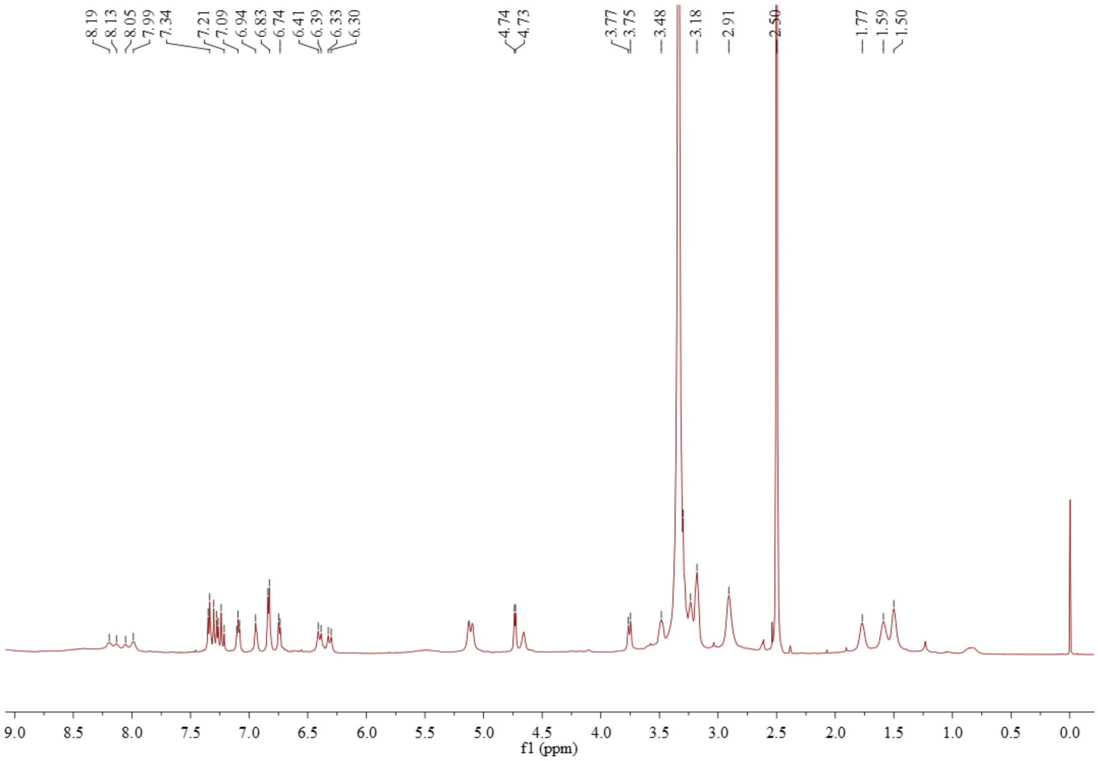

C

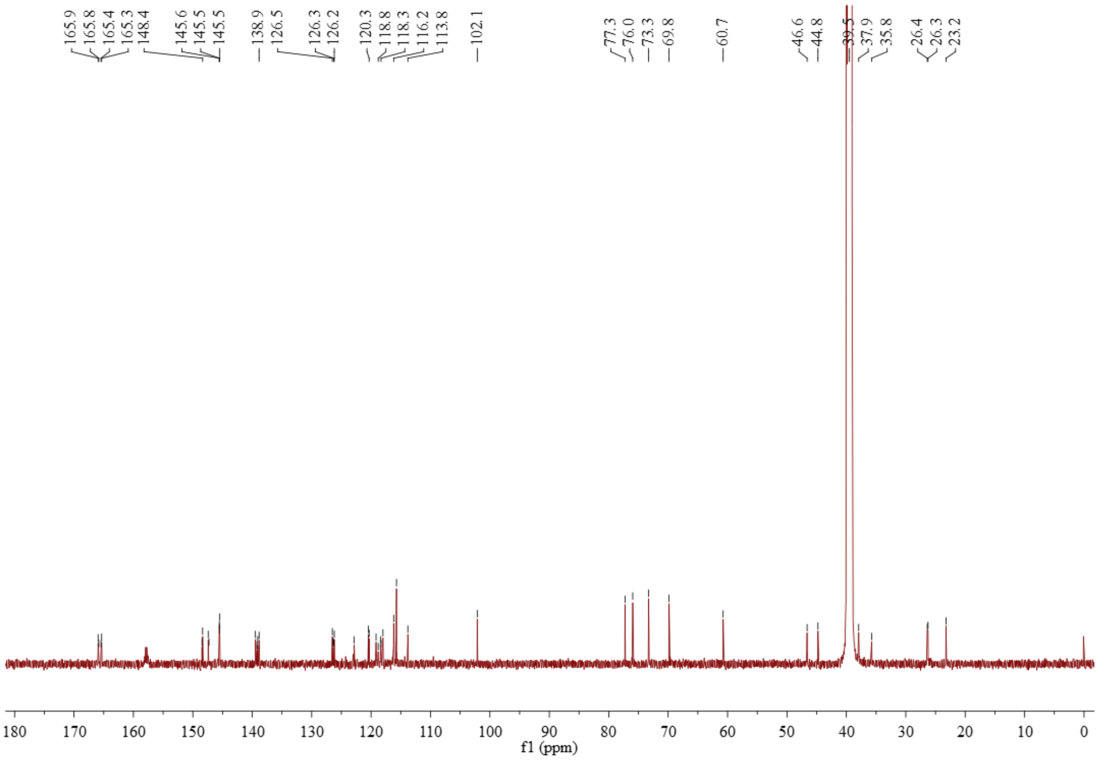

165.9
165.8
165.4
165.3
148.4
145.6
145.5
145.5
138.9
126.5
126.3
126.2
120.3
118.8
118.3
116.2
113.8
102.1
77.3
76.0
73.3
69.8
60.7
46.6
44.8
39.5
37.9
35.8
26.4
26.3
23.2
180 170 160 150 140 130 120 110 100 90 80 70 60 50 40 30 20 10 0
f1 (ppm)

D

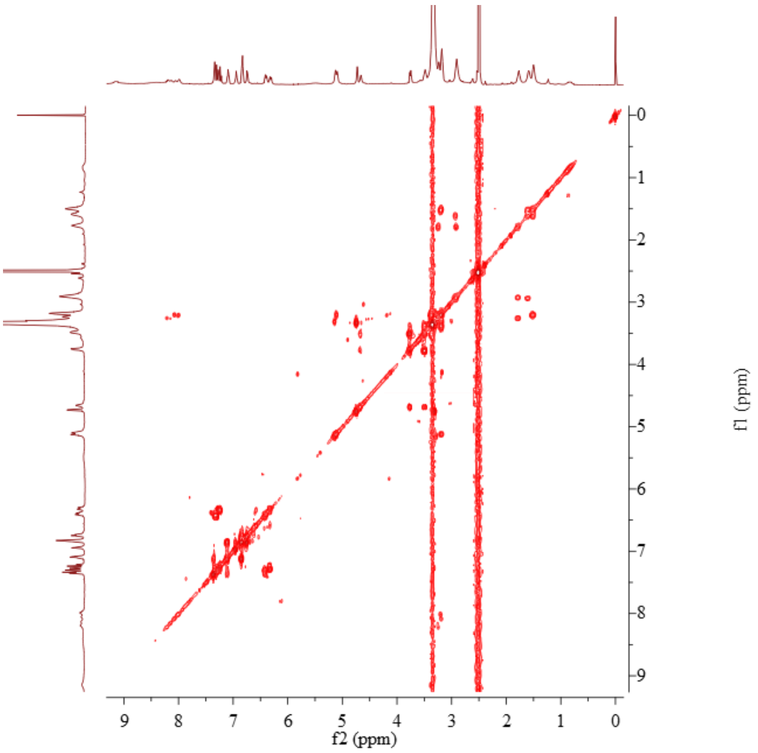

0 1 2 3 4 5 6 7 8 9
f1 (ppm)
9 8 7 6 5 4 3 2 1 0
f2 (ppm)

E

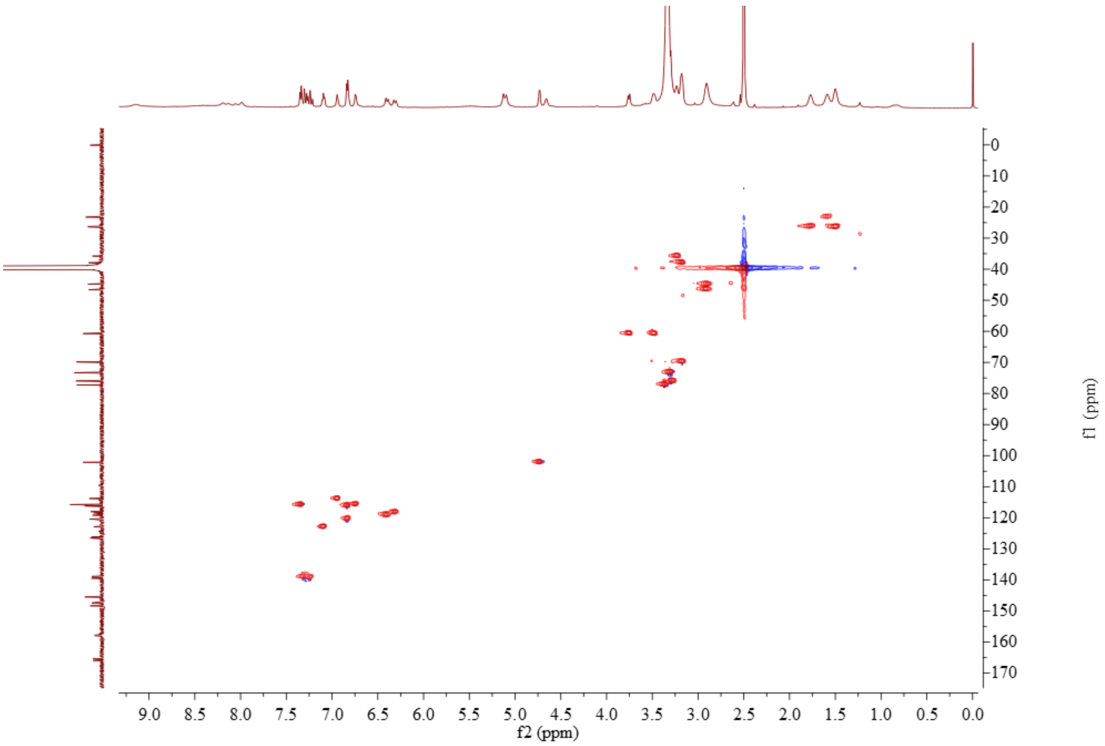

0
10
20
30
40
50
60
70
80
90
100
110
120
130
140
150
160
170
f1 (ppm)
9.0
8.5
8.0
7.5
7.0
6.5
6.0
5.5
5.0
4.5
4.0
3.5
3.0
2.5
2.0
1.5
1.0
0.5
0.0
f2 (ppm)

F

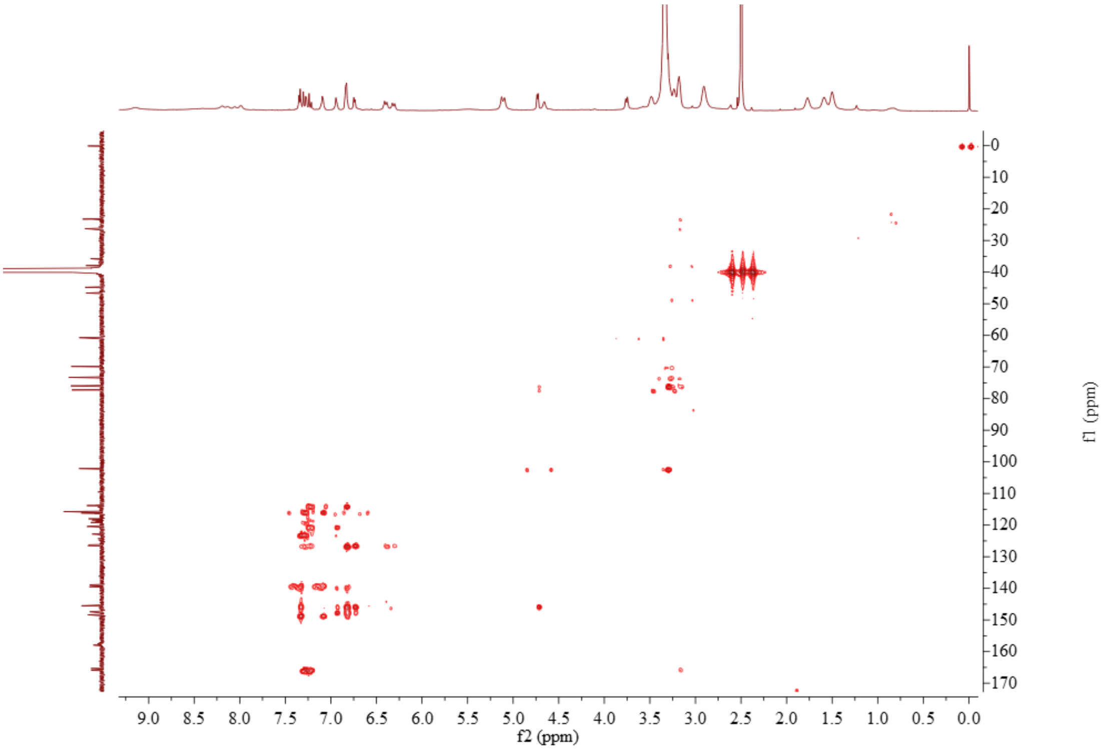

0
10
20
30
40
50
60
70
80
90
100
110
120
130
140
150
160
170
f1 (ppm)
9.0
8.5
8.0
7.5
7.0
6.5
6.0
5.5
5.0
4.5
4.0
3.5
3.0
2.5
2.0
1.5
1.0
0.5
0.0
f2 (ppm)

G

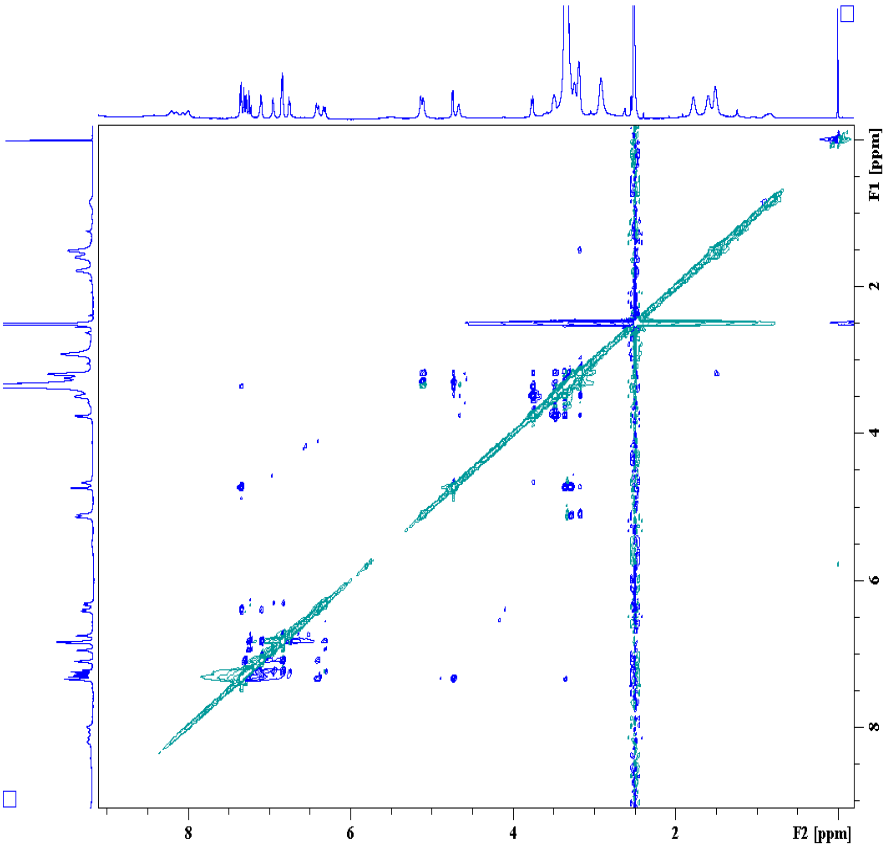

Supplementary Figure 19. HRESIMS and NMR spectra of 7/8.

(A) HRESIMS spectrum; (B) ^{1}H NMR spectrum in DMSO-d_6 at 600 MHz; (C) ^{13}C NMR spectrum in DMSO-d_6 at 150 MHz ; (D) ^{1}H-^{1}H COSY spectrum in DMSO-d_6 at 600 MHz; (E) HSQC spectrum in DMSO-d_6 at 600 MHz; (F) HMBC spectrum in DMSO-d_6 at 600 MHz; (G) ROESY spectrum in DMSO-d_6 at 600 MHz.

A

Elemental Composition Report **Page 1**

Single Mass Analysis
Tolerance = 5.0 mDa / DBE: min = -1.5, max = 50.0
Element prediction: Off
Number of isotope peaks used for i-FIT = 3

Monoisotopic Mass, Even Electron Ions
507 formula(e) evaluated with 4 results within limits (up to 50 best isotopic matches for each mass)
Elements Used:
C: 0-40 H: 0-60 N: 0-5 O: 0-20 Na: 0-1
89787-469-1
20220221054 54 (0.449) 1: TOF MS ES+
7.99e+004

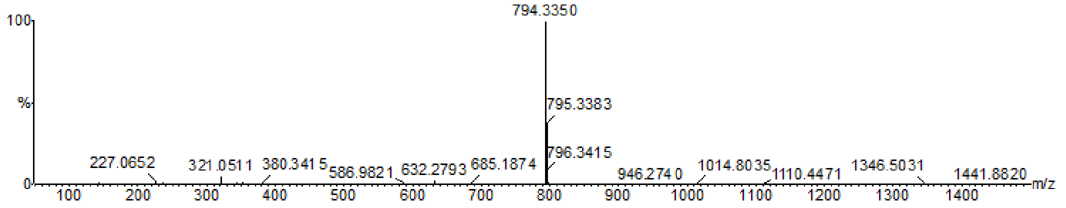

Minimum:				-1.5				
Maximum:		5.0	10.0	50.0				
Mass	Calc. Mass	mDa	PPM	DBE	i-FIT	Norm	Conf(%)	Formula
794.3350	794.3348	0.2	0.3	13.5	272.2	0.918	39.94	C37 H52 N3 O16
	794.3324	2.6	3.3	10.5	272.4	1.200	30.13	C35 H53 N3 O16 Na
	794.3364	-1.4	-1.8	14.5	273.0	1.788	16.72	C40 H53 N O14 Na
	794.3307	4.3	5.4	9.5	273.3	2.025	13.21	C32 H52 N5 O18

B

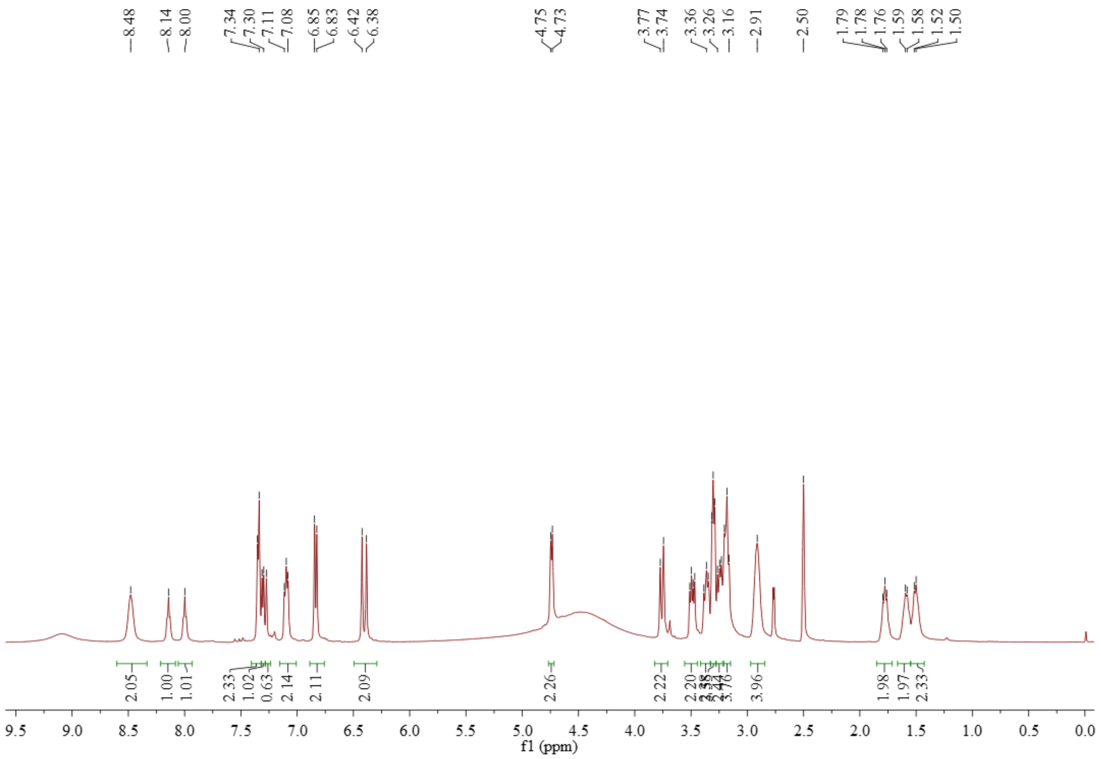

C

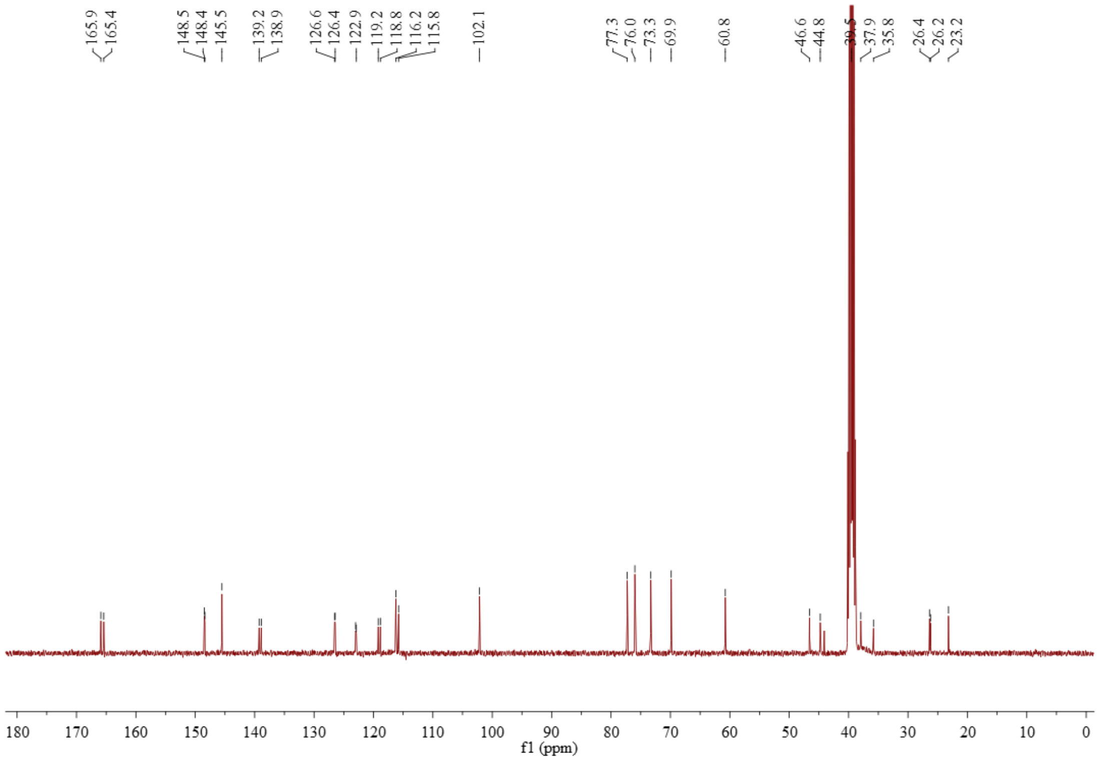
165.9
165.4
148.5
148.4
145.5
139.2
138.9
126.6
126.4
122.9
119.2
118.8
116.2
115.8
102.1
77.3
76.0
73.3
69.9
60.8
46.6
44.8
37.9
35.8
26.4
26.2
23.2
180 170 160 150 140 130 120 110 100 90 80 70 60 50 40 30 20 10 0
f1 (ppm)

D

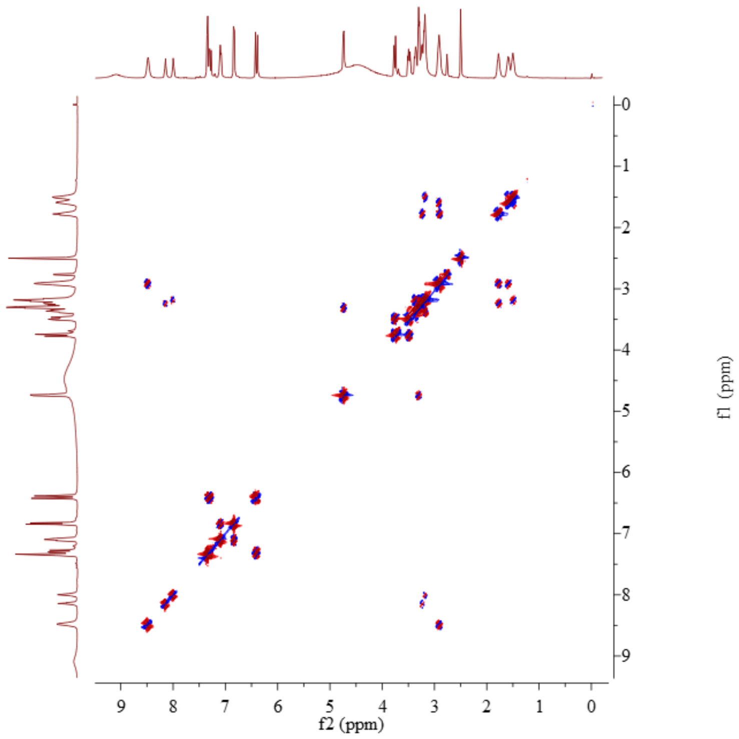
9 8 7 6 5 4 3 2 1 0
f2 (ppm)
0 1 2 3 4 5 6 7 8 9
f1 (ppm)

E

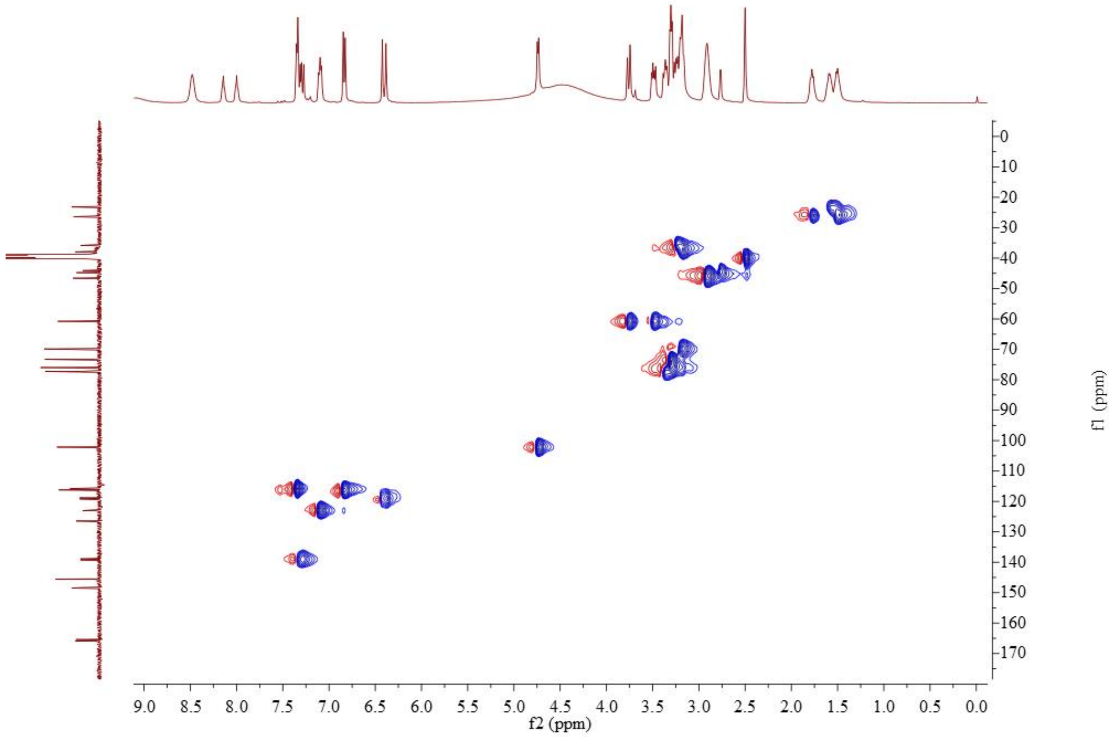
9.0
8.5
8.0
7.5
7.0
6.5
6.0
5.5
5.0
4.5
4.0
3.5
3.0
2.5
2.0
1.5
1.0
0.5
0.0
f2 (ppm)
f1 (ppm)
0
10
20
30
40
50
60
70
80
90
100
110
120
130
140
150
160
170

F

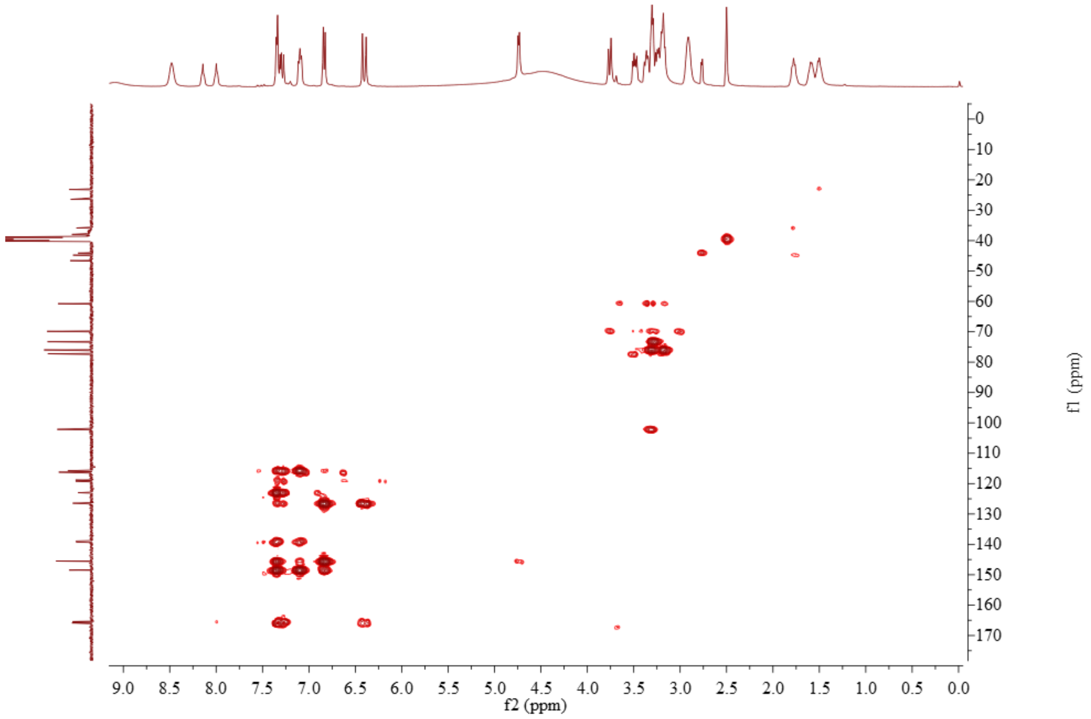
9.0
8.5
8.0
7.5
7.0
6.5
6.0
5.5
5.0
4.5
4.0
3.5
3.0
2.5
2.0
1.5
1.0
0.5
0.0
f2 (ppm)
f1 (ppm)
0
10
20
30
40
50
60
70
80
90
100
110
120
130
140
150
160
170

G

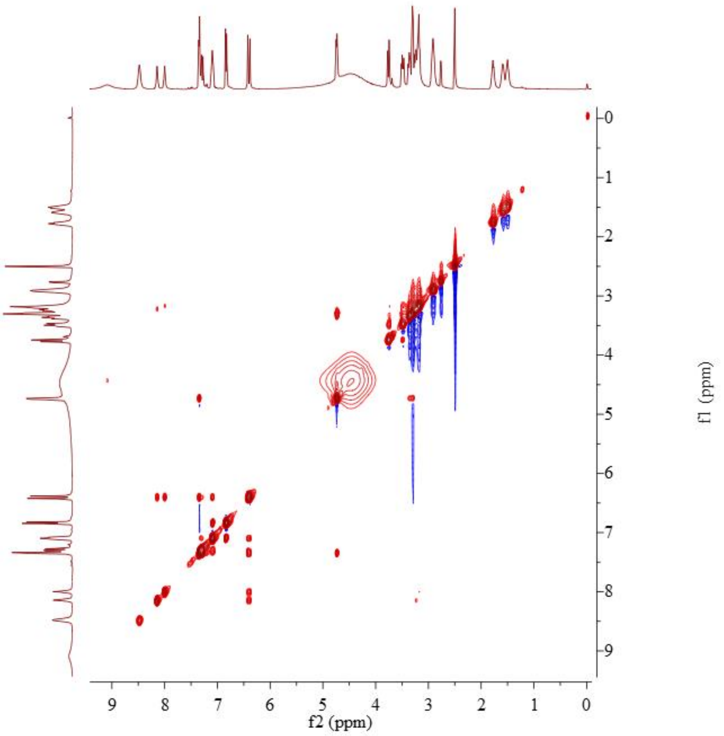

Supplementary Figure 20. HRESIMS and NMR spectra of 9.

(A) HRESIMS spectrum; (B) ^{1}H NMR spectrum in DMSO-d_6 at 400 MHz; (C) ^{13}C NMR spectrum in DMSO-d_6 at 100 MHz ; (D) ^{1}H-^{1}H COSY spectrum in DMSO-d_6 at 400 MHz; (E) HSQC spectrum in DMSO-d_6 at 400 MHz; (F) HMBC spectrum in DMSO-d_6 at 400 MHz; (G) ROESY spectrum in DMSO-d_6 at 400 MHz.

A

Elemental Composition Report **Page 1**

Single Mass Analysis
Tolerance = 20.0 PPM / DBE: min = -1.5, max = 50.0
Element prediction: Off
Number of isotope peaks used for i-FIT = 2

Monoisotopic Mass, Even Electron Ions
124 formula(e) evaluated with 2 results within limits (all results (up to 1000) for each mass)
Elements Used:
C: 0-40 H: 0-60 N: 2-4 O: 0-20
106657-1
20211122-57 48 (0.404) 1: TOF MS ES+ 4.03e+003

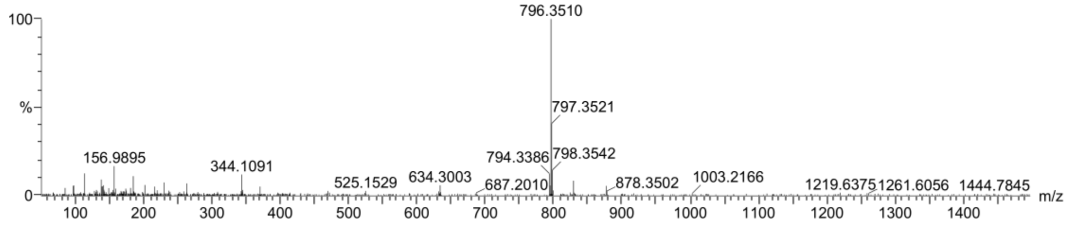

Minimum:				-1.5		
Maximum:		5.0	20.0	50.0		
Mass	Calc. Mass	mDa	PPM	DBE	Conf(%)	Formula
796.3510	796.3504	0.6	0.8	12.5	91.46	C37 H54 N3 O16
	796.3352	15.8	19.8	8.5	8.54	C33 H54 N3 O19

B

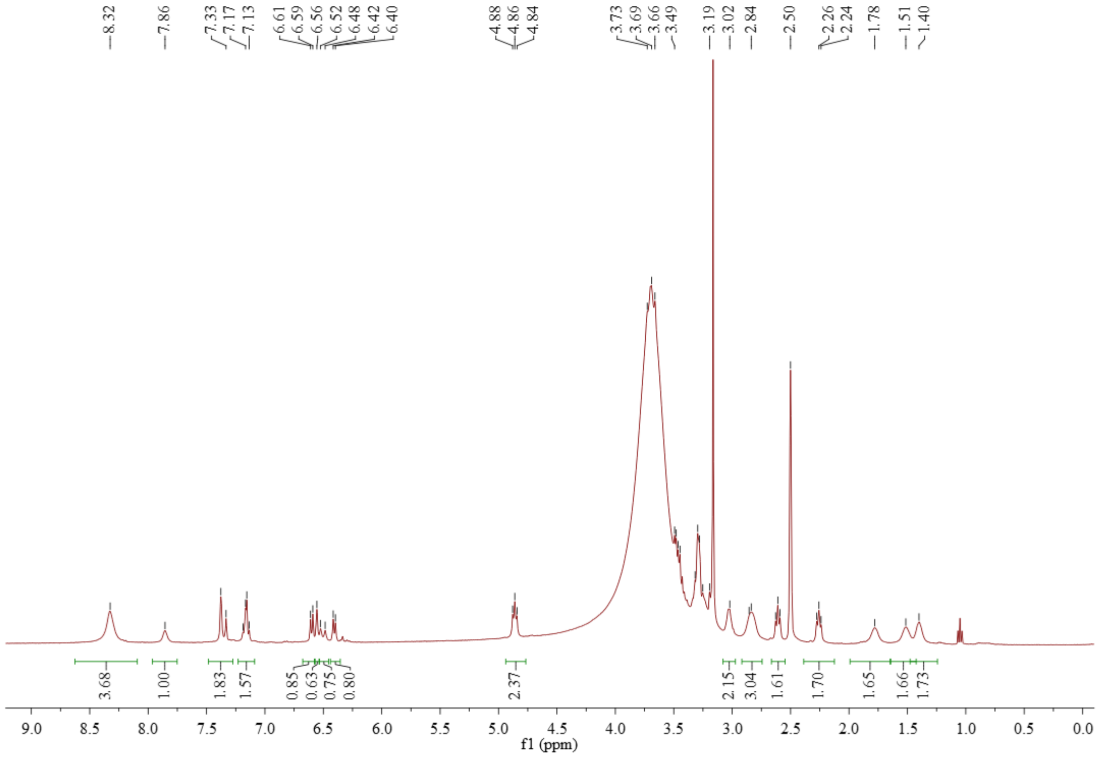

C

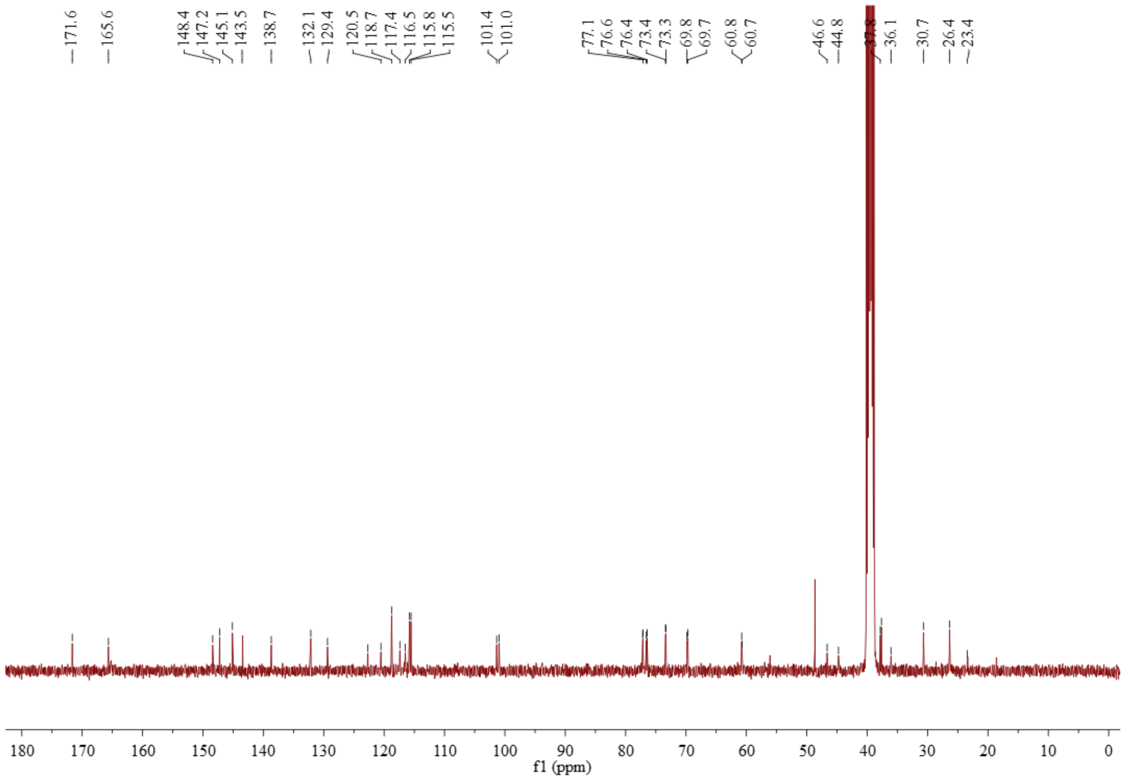

171.6
165.6
148.4
147.2
145.1
143.5
138.7
132.1
129.4
120.5
118.7
117.4
116.5
115.8
115.5
101.4
101.0
77.1
76.6
76.4
73.4
73.3
69.8
69.7
60.8
60.7
46.6
44.8
37.8
36.1
30.7
26.4
23.4
180 170 160 150 140 130 120 110 100 90 80 70 60 50 40 30 20 10 0
f1 (ppm)

D

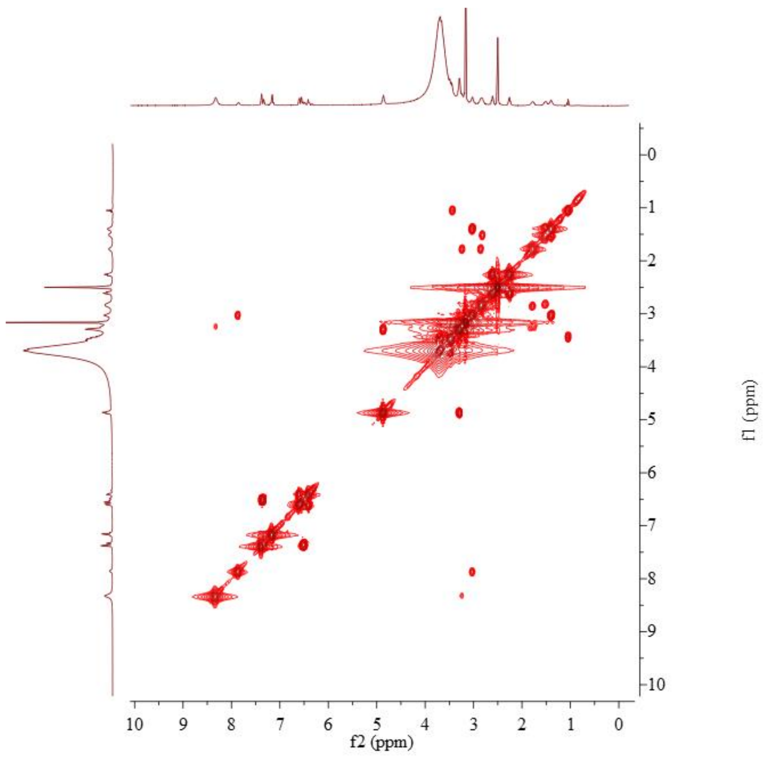

0 1 2 3 4 5 6 7 8 9 10
f1 (ppm)
10 9 8 7 6 5 4 3 2 1 0
f2 (ppm)

E

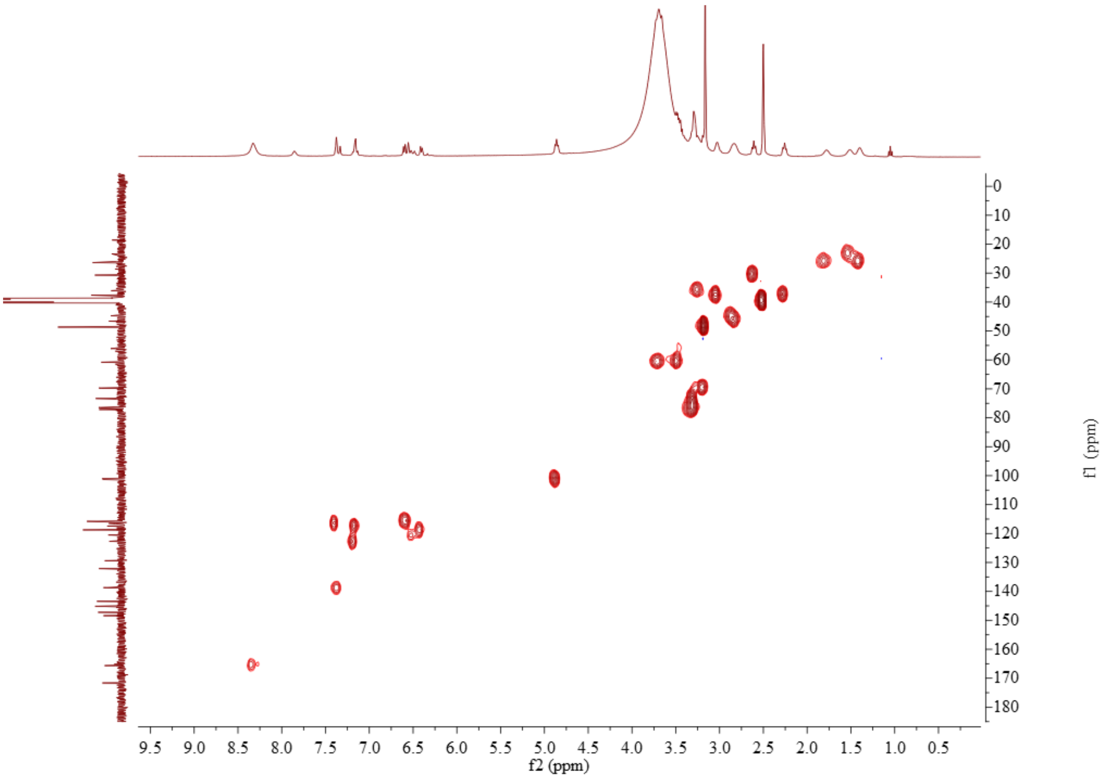
9.5 9.0 8.5 8.0 7.5 7.0 6.5 6.0 5.5 5.0 4.5 4.0 3.5 3.0 2.5 2.0 1.5 1.0 0.5
f2 (ppm)
0 10 20 30 40 50 60 70 80 90 100 110 120 130 140 150 160 170 180
f1 (ppm)

F

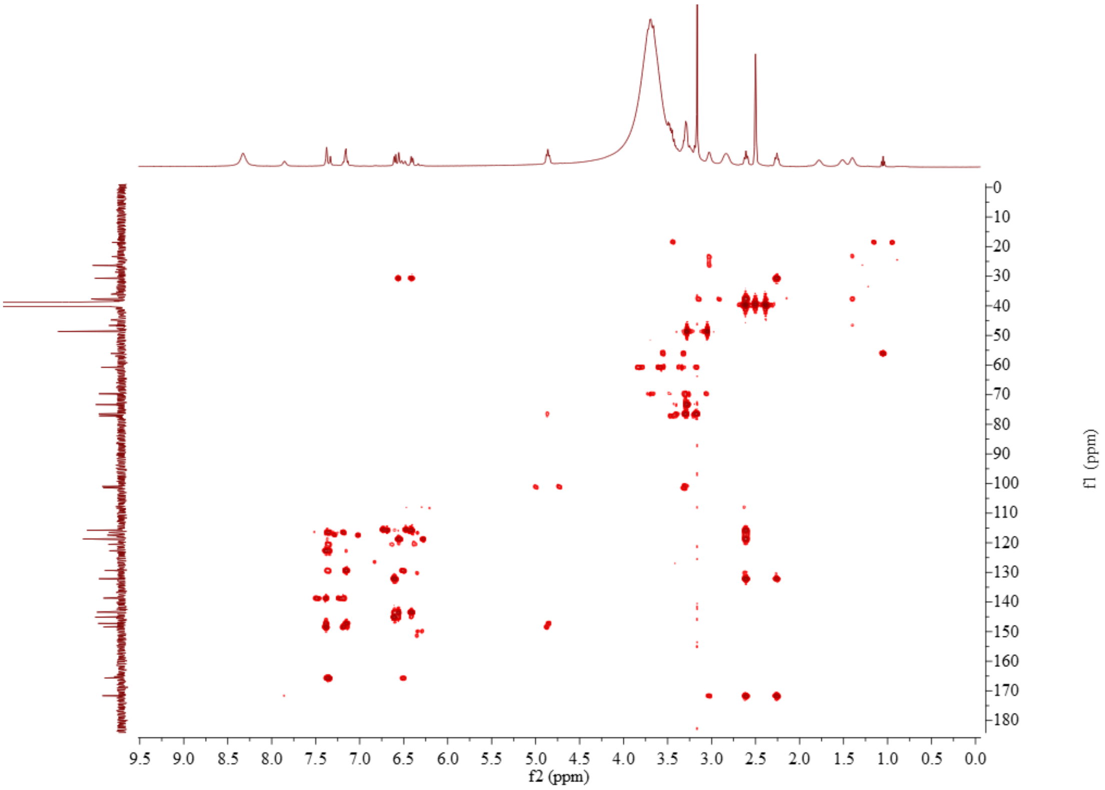
9.5 9.0 8.5 8.0 7.5 7.0 6.5 6.0 5.5 5.0 4.5 4.0 3.5 3.0 2.5 2.0 1.5 1.0 0.5 0.0
f2 (ppm)
0 10 20 30 40 50 60 70 80 90 100 110 120 130 140 150 160 170 180
f1 (ppm)

G

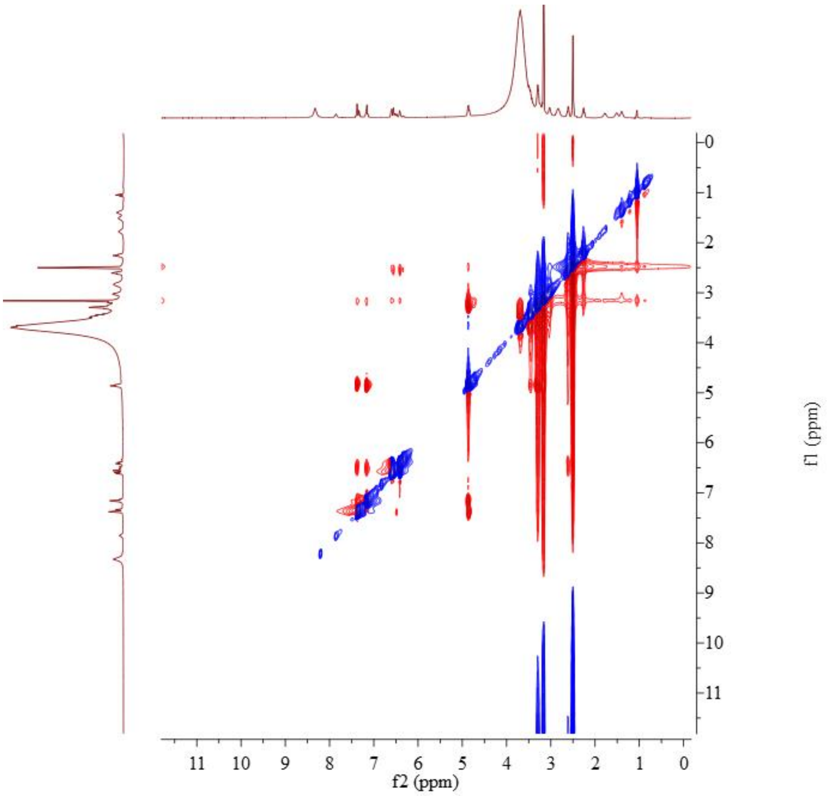

Supplementary Figure 21. HRESIMS and NMR spectra of 15.

(A) HRESIMS spectrum; (B) ^{1}H NMR spectrum in DMSO-d_6 at 400 MHz; (C) ^{13}C NMR spectrum in DMSO-d_6 at 100 MHz ; (D) ^{1}H-^{1}H COSY spectrum in DMSO-d_6 at 400 MHz; (E) HSQC spectrum in DMSO-d_6 at 400 MHz; (F) HMBC spectrum in DMSO-d_6 at 400 MHz; (G) ROESY spectrum in DMSO-d_6 at 400 MHz.

A

Elemental Composition Report

Page 1

Single Mass Analysis

Tolerance = 20.0 PPM / DBE: min = -1.5, max = 50.0
Element prediction: Off
Number of isotope peaks used for i-FIT = 2

Monoisotopic Mass, Even Electron Ions
123 formula(e) evaluated with 2 results within limits (all results (up to 1000) for each mass)
Elements Used:
C: 0-40 H: 0-60 N: 2-4 O: 0-20
106-469-3-2
20211122-59 41 (0.341)

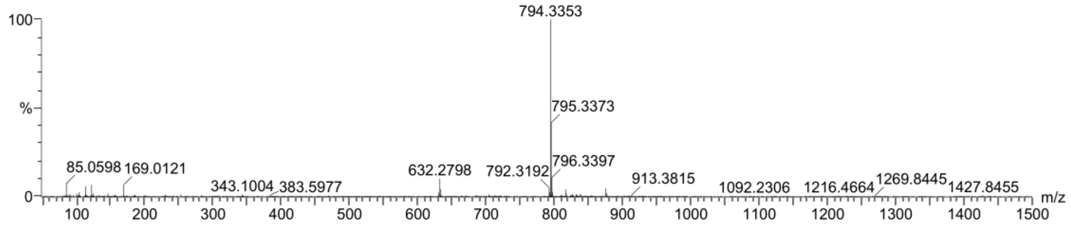

Minimum:				-1.5		
Maximum:		5.0	20.0	50.0		

Mass	Calc. Mass	mDa	PPM	DBE	Conf(%)	Formula
794.3353	794.3348	0.5	0.6	13.5	99.79	C37 H52 N3 O16
	794.3195	15.8	19.9	9.5	0.21	C33 H52 N3 O19

B

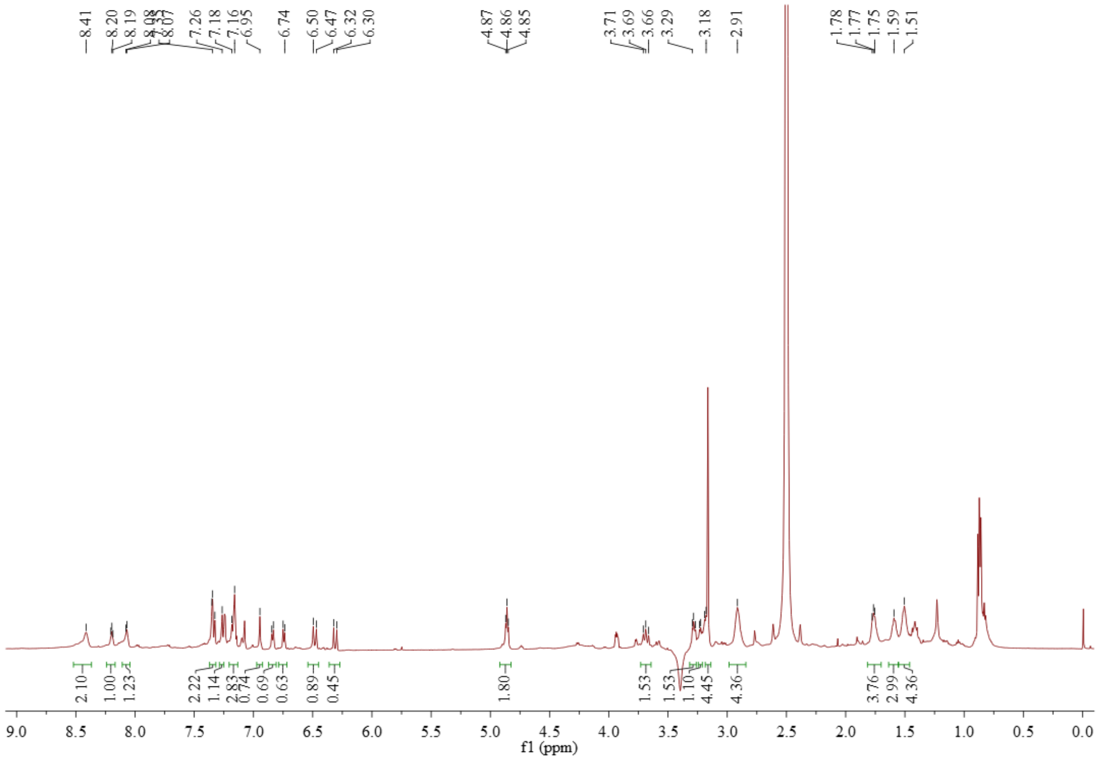

C

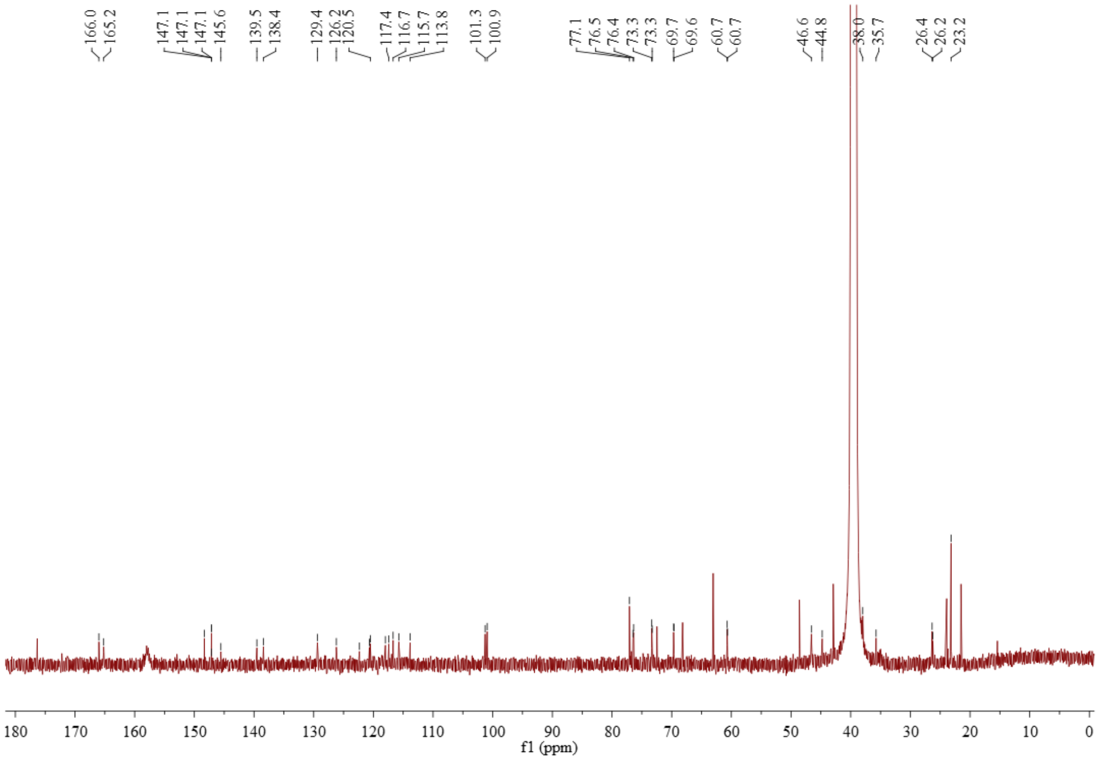
166.0
165.2
147.1
147.1
147.1
145.6
139.5
138.4
129.4
126.2
120.5
117.4
116.7
115.7
113.8
101.3
100.9
77.1
76.5
76.4
73.3
73.3
69.7
69.6
60.7
60.7
46.6
44.8
38.0
35.7
26.4
26.2
23.2
180 170 160 150 140 130 120 110 100 90 80 70 60 50 40 30 20 10 0
f1 (ppm)

D

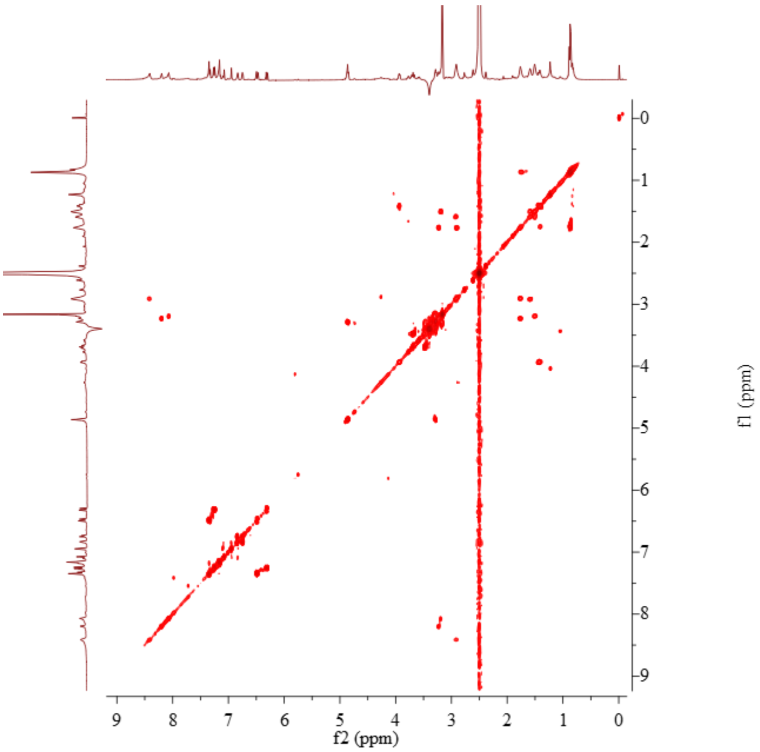
9 8 7 6 5 4 3 2 1 0
f2 (ppm)
0 1 2 3 4 5 6 7 8 9
f1 (ppm)

E

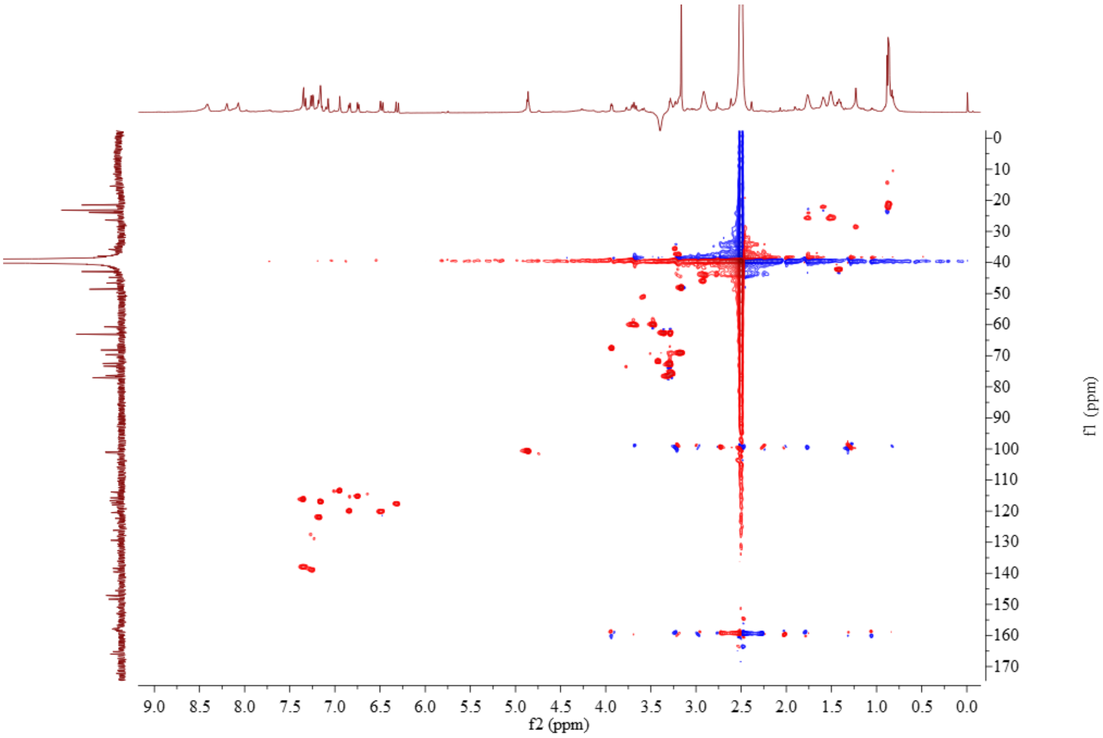

F

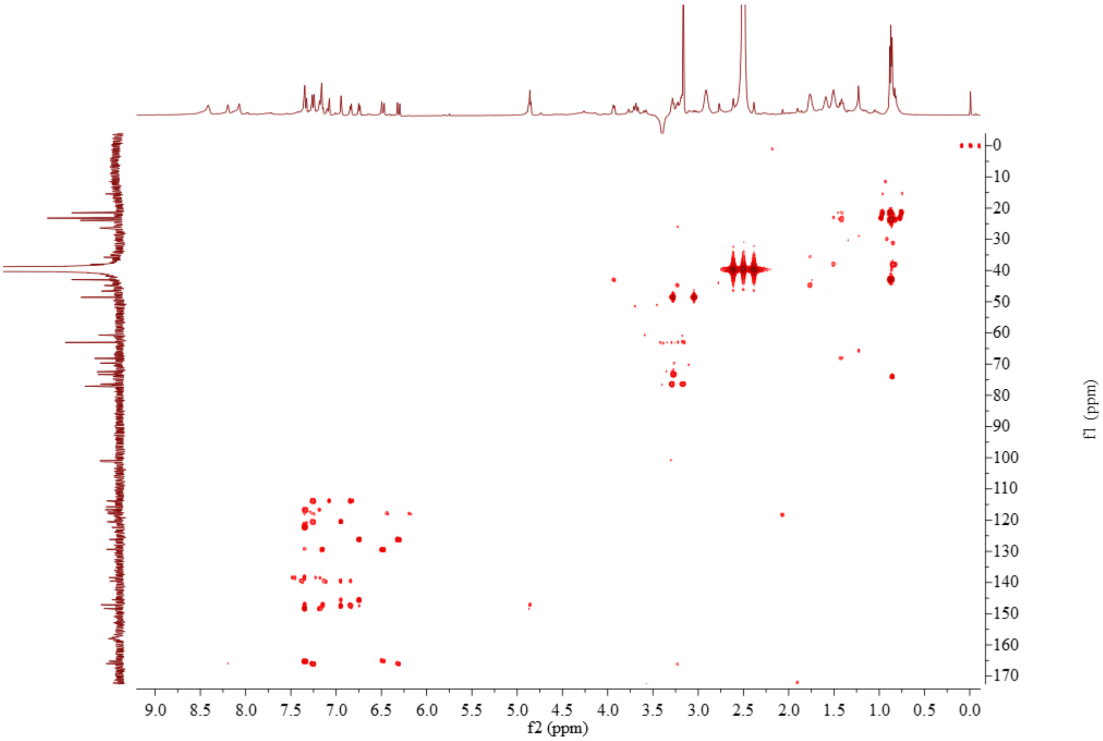

G

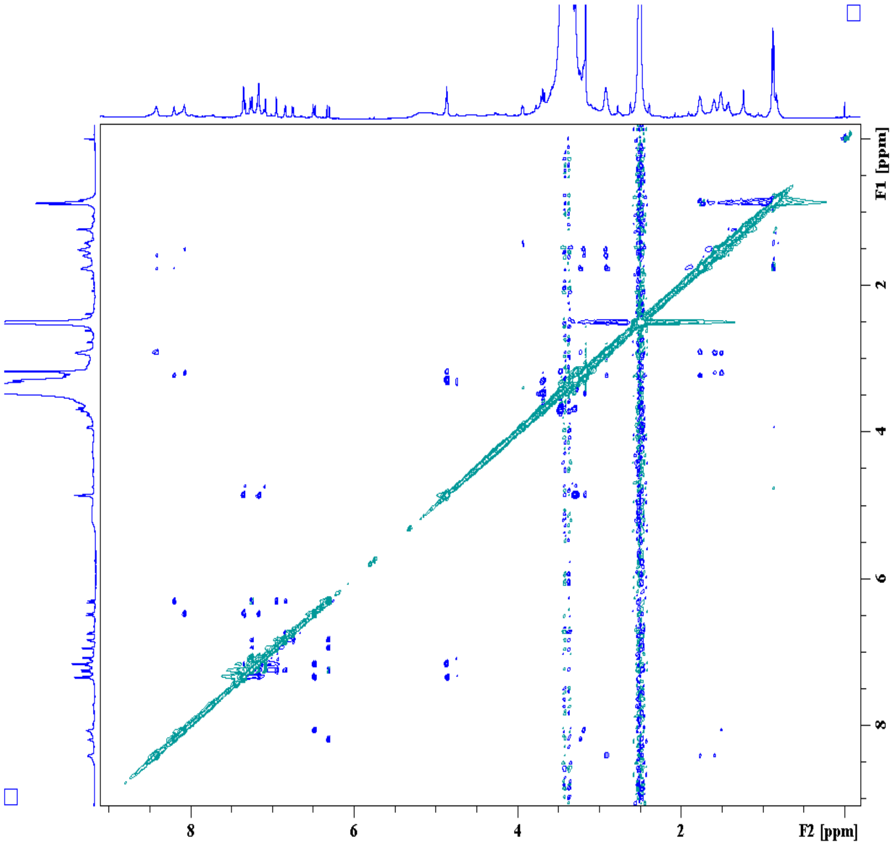

Supplementary Figure 22. HRESIMS and NMR spectra of 16.

(A) HRESIMS spectrum; (B) ^{1}H NMR spectrum in DMSO-d_6 at 600 MHz; (C) ^{13}C NMR spectrum in DMSO-d_6 at 150 MHz ; (D) ^{1}H-^{1}H COSY spectrum in DMSO-d_6 at 600 MHz; (E) HSQC spectrum in DMSO-d_6 at 600 MHz; (F) HMBC spectrum in DMSO-d_6 at 600 MHz; (G) ROESY spectrum in DMSO-d_6 at 600 MHz.

A

Elemental Composition Report **Page 1**

Single Mass Analysis
Tolerance = 20.0 PPM / DBE: min = -1.5, max = 50.0
Element prediction: Off
Number of isotope peaks used for i-FIT = 2

Monoisotopic Mass, Even Electron Ions
123 formula(e) evaluated with 2 results within limits (all results (up to 1000) for each mass)
Elements Used:
C: 0-40 H: 0-60 N: 2-4 O: 0-20
106-469-3-1
20211122-58 46 (0.389) 1: TOF MS ES+ 1.06e+004

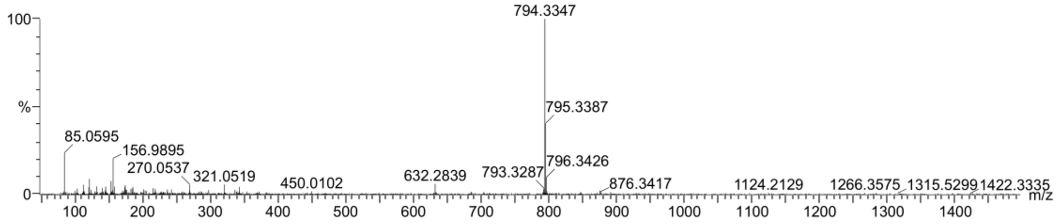

Minimum:				-1.5		
Maximum:		5.0	20.0	50.0		

Mass	Calc. Mass	mDa	PPM	DBE	Conf(%)	Formula
794.3347	794.3348	-0.1	-0.1	13.5	93.65	C37 H52 N3 O16
	794.3195	15.2	19.1	9.5	6.35	C33 H52 N3 O19

B

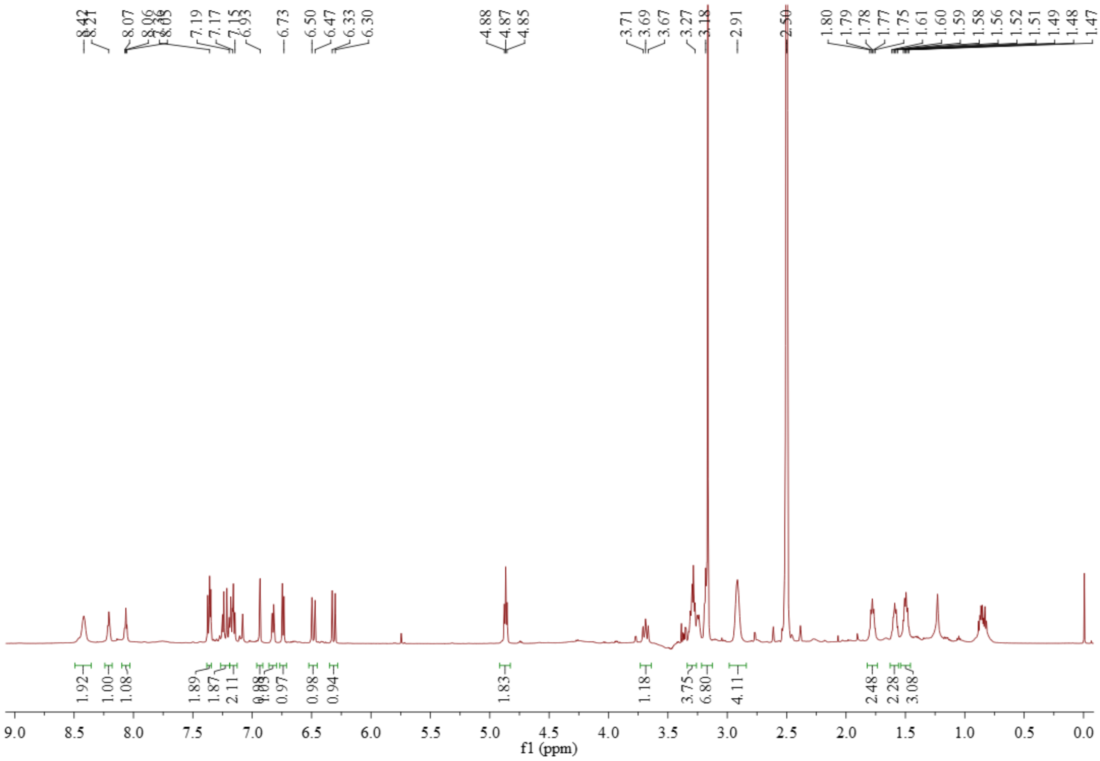

C

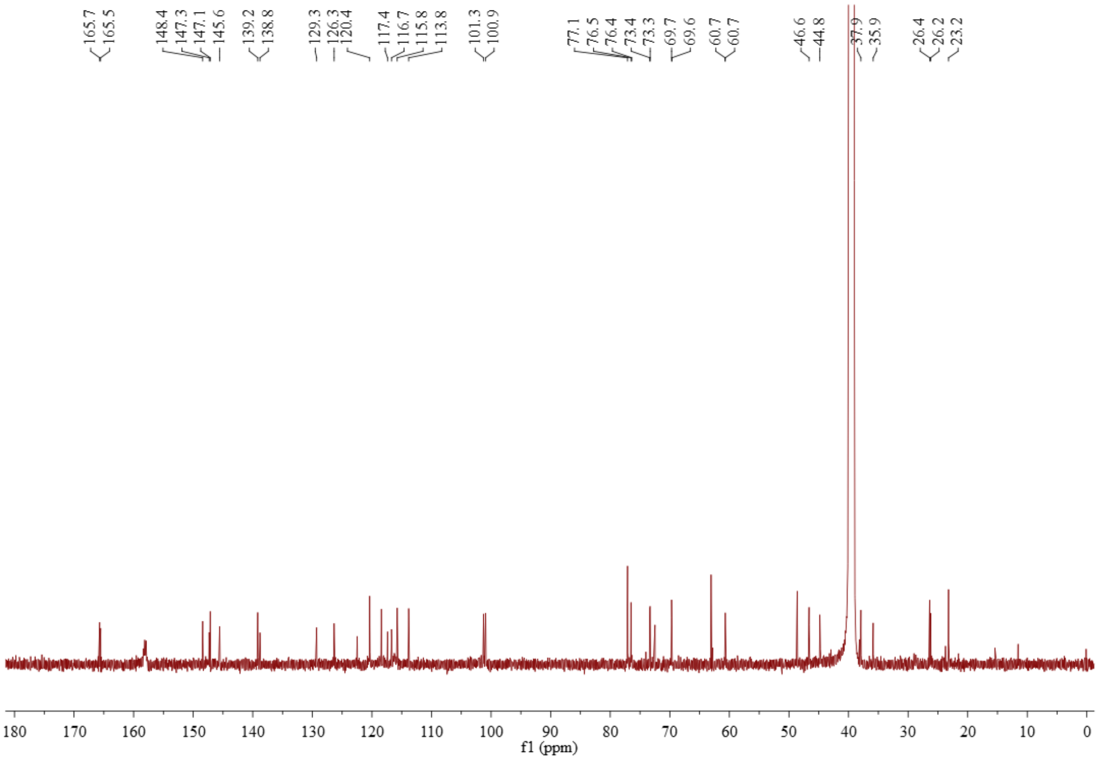

165.7
165.5
148.4
147.3
147.1
145.6
139.2
138.8
129.3
126.3
120.4
117.4
116.7
115.8
113.8
101.3
100.9
77.1
76.5
76.4
73.4
73.3
69.7
69.6
60.7
60.7
46.6
44.8
37.9
35.9
26.4
26.2
23.2
f1 (ppm)

D

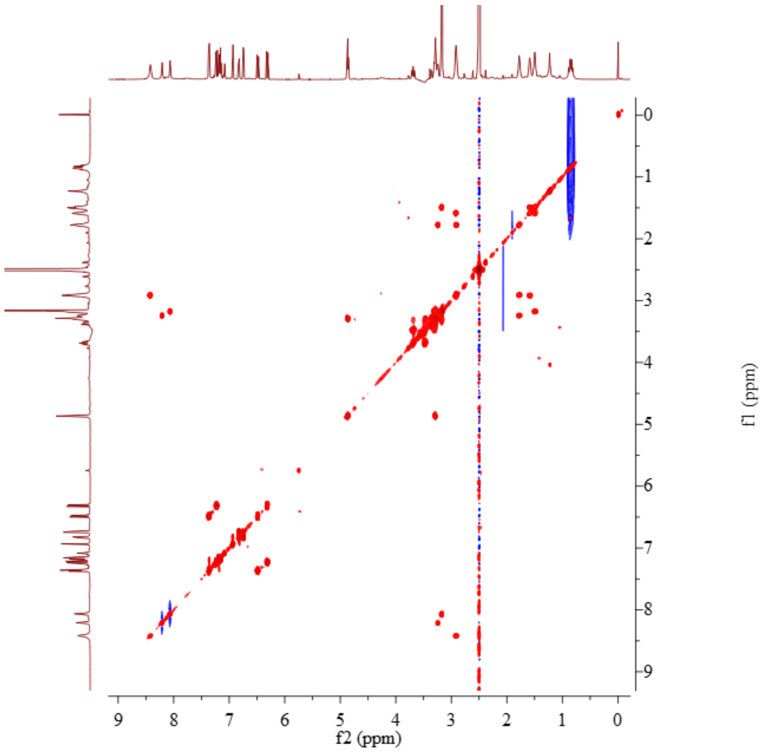

f2 (ppm)
f1 (ppm)

E

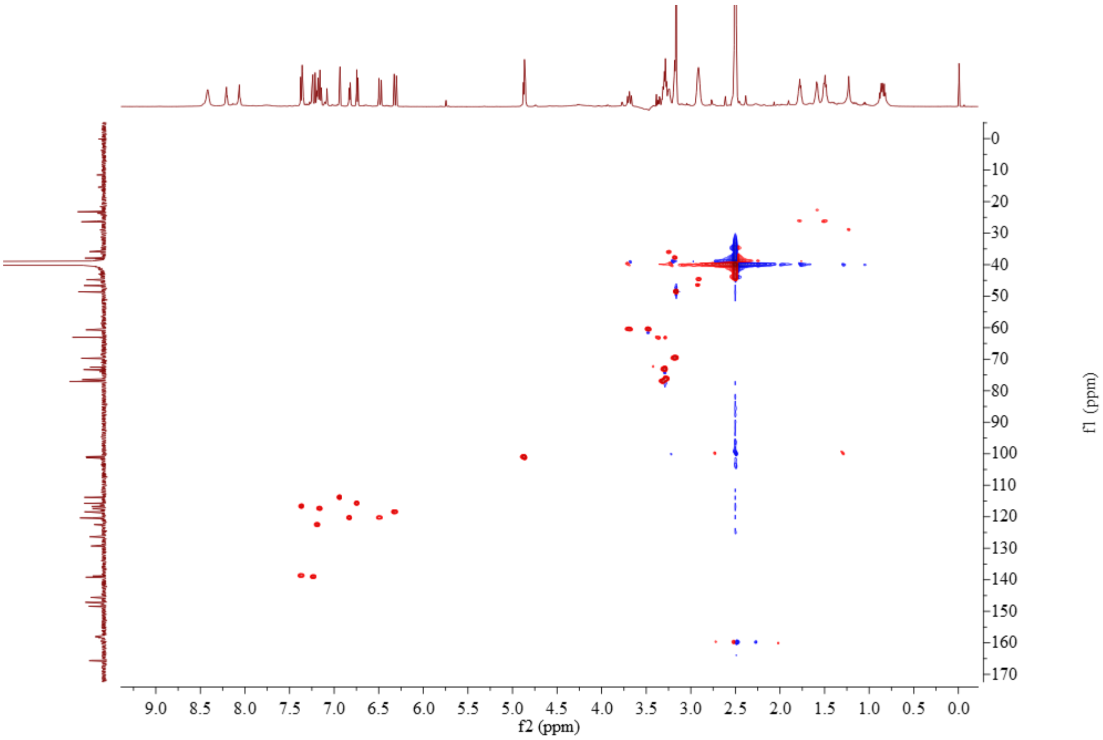
9.0 8.5 8.0 7.5 7.0 6.5 6.0 5.5 5.0 4.5 4.0 3.5 3.0 2.5 2.0 1.5 1.0 0.5 0.0
f2 (ppm)
0 10 20 30 40 50 60 70 80 90 100 110 120 130 140 150 160 170
f1 (ppm)

F

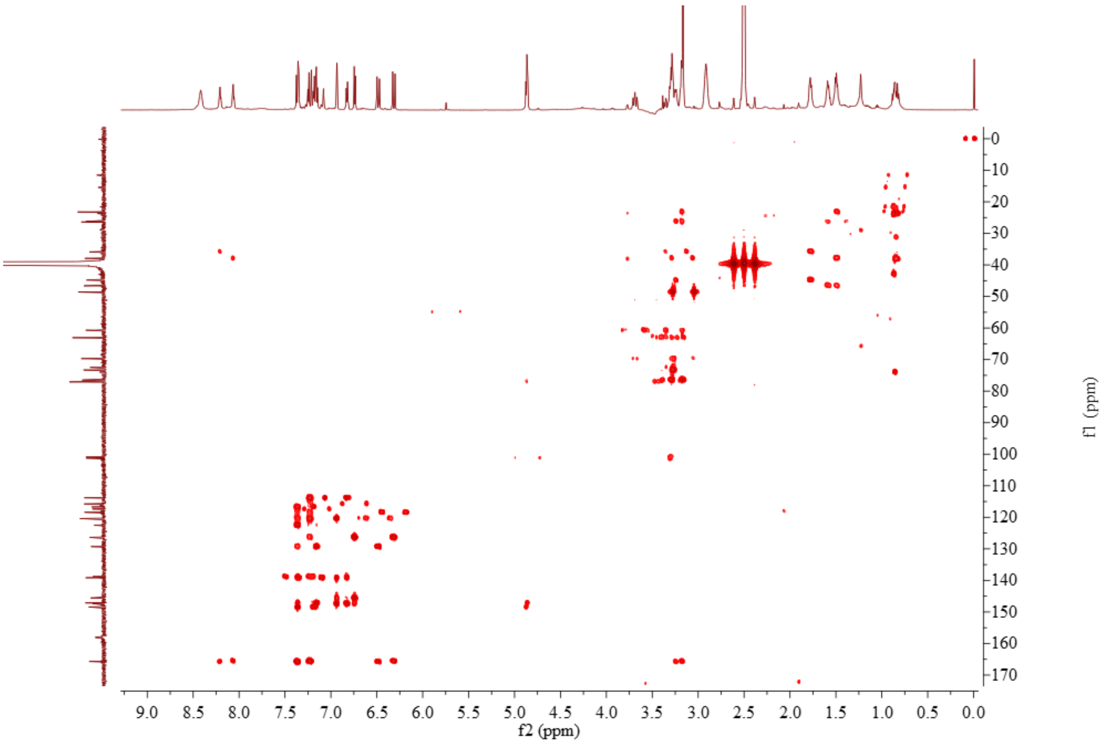
9.0 8.5 8.0 7.5 7.0 6.5 6.0 5.5 5.0 4.5 4.0 3.5 3.0 2.5 2.0 1.5 1.0 0.5 0.0
f2 (ppm)
0 10 20 30 40 50 60 70 80 90 100 110 120 130 140 150 160 170
f1 (ppm)

G

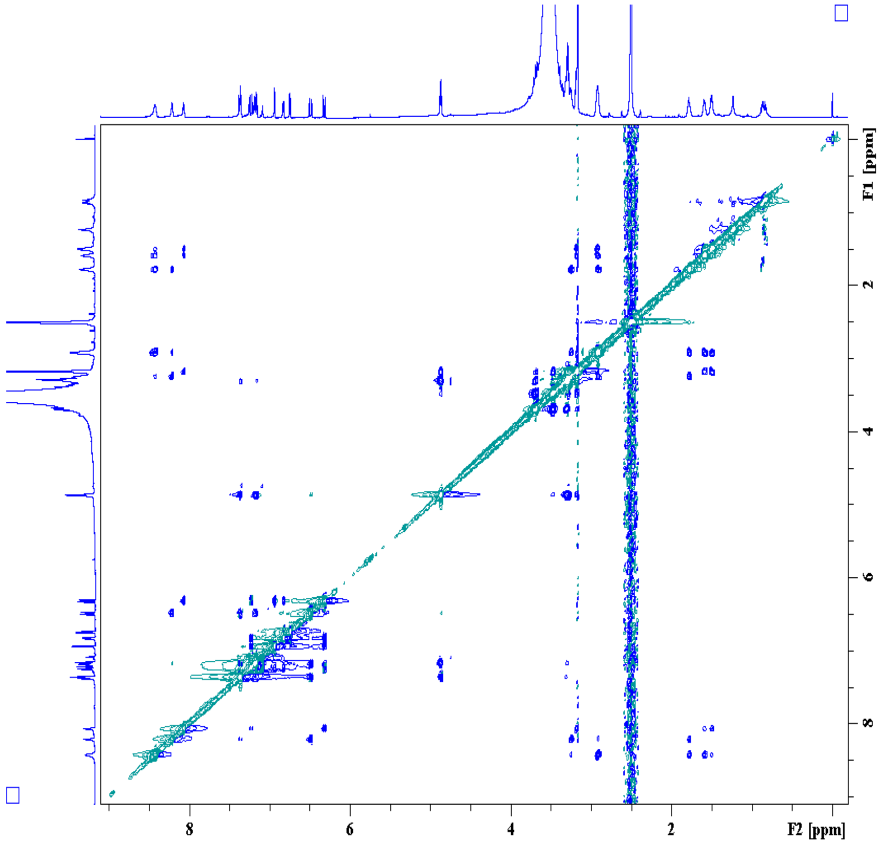

Supplementary Figure 23. HRESIMS and NMR spectra of 17.

(A) HRESIMS spectrum; (B) ^{1}H NMR spectrum in DMSO-d_6 at 600 MHz; (C) ^{13}C NMR spectrum in DMSO-d_6 at 150 MHz ; (D) ^{1}H-^{1}H COSY spectrum in DMSO-d_6 at 600 MHz; (E) HSQC spectrum in DMSO-d_6 at 600 MHz; (F) HMBC spectrum in DMSO-d_6 at 600 MHz; (G) ROESY spectrum in DMSO-d_6 at 600 MHz.

Supplementary Tables

Supplementary Table 1. GenBank ID of UGTs used for phylogenetic analysis.

UGTs	UGT71B1	UGT71B5	UGT71B6	UGT71B8	UGT71C1
Genbank ID	NP_188812.1	NP_193263.1	NP_188815.2	NP_188817.1	NP_180536.1
UGTs	UGT71C2	UGT71C3	UGT71C4	UGT71C5	UGT71D1
Genbank ID	NP_180535.1	NP_172206.1	NP_563784.2	NP_172204.1	NP_180534.1
UGTs	UGT88A1	UGT72B1	UGT72C1	UGT72D1	UGT72E2
Genbank ID	NP_850597.1	NP_192016.1	O23205.3	Q9ZU72.1	Q9LVR1.1
UGTs	UGT72E3	UGT80A2	OsCGT	TOGT1	UGT73C1
Genbank ID	O81498.1	CAE7683128.1	Q5VMI0.1	KAG6571965.1	XP_020884151.1
UGTs	UGT73C5	UGT73C6	UGT89C1	UGT89B1	UGT78D1
Genbank ID	OAP09184.1	OAP07438.1	Q9LNE6.1	OAP14423.1	OAP13716.1
UGTs	UGT78D2	UGT78D3	UGT78A7	UGT78G1	UGT74F2
Genbank ID	OAO89857.1	OAO94865.1	BBD34311.1	KAG7023401.1	QWT69380.1
UGTs	UGT74B1	UGT74C1	UGT74E2	UGT75B1	UGT75C1
Genbank ID	OAP18511.1	CAE7415096.1	OAP17332.1	OAP16927.1	KAG6593628.1
UGTs	UGT84A1	UGT84A2	UGT84A3	UGT84B1	UGT76C1
Genbank ID	OAO99238.1	OAP06181.1	OAP00592.1	OAP11221.1	OAO89564.1
UGTs	UGT76C2	UGT85A1	UGTcis-ZOG1	UGTcis-ZOG2	UGTZOG1
Genbank ID	OAO93987.1	OAP13723.1	AAK53551.1	AAL92460.1	AAD04166.1
UGTs	UGTZOX1				
Genbank ID	AAD51778.1				

Supplementary Table 2. Identities and positives of LbUGT1-5 to each other.

Identities/ Positives (%)	LbUGT1	LbUGT2	LbUGT3	LbUGT4	LbUGT5
LbUGT1		29/47	53/70	32/49	43/61
LbUGT2	29/47		28/45	30/47	30/46
LbUGT3	53/70	28/45		32/52	45/64
LbUGT4	32/49	30/47	32/52		31/49
LbUGT5	43/61	30/46	45/64	31/49	

Supplementary Table 3. Data collection and refinement statistics.

**Name**	LbUGT1-UDP complex	LbUGT3-UDP complex
**PDB ID**	8WP5	8W53
**Data collection**		
Space group	*P4₁2₁2*	*P22₁2₁*
Cell dimensions		
a, b, c (Å)	122.24, 122.24, 81.52	72.44, 80.84, 180.24
α, β, γ (°)	90.00, 90.00, 90.00	90.00, 90.00, 90.00
Resolution (Å) [a]	48.90 - 2.57	31.03 - 2.43
	(2.64 - 2.57)	(2.49 - 2.43)
R_{merge}	0.159 (2.040)	0.128 (0.986)
R_{pim}	0.044 (0.562)	0.037 (0.279)
I/σI	18.2 (2.2)	11.6 (2.5)
Redundancy	25.6 (26.8)	12.9 (13.3)
Completeness (%)	100.0 (99.9)	100.0 (100.0)
CC1/2	0.999 (0.794)	0.999 (0.617)
**Refinement**		
Resolution (Å)	48.90-2.57	31.02 - 2.43
No. reflections	20229	40714
R_{work} / R_{free}	0.243/0.286	0.219/0.266
No. non-H atoms		
Protein	3736	7507
Ligand	26	56
Water	21	45
B factor(Å^2)		
Protein	67.8	69.1
Ligand	67.6	60.4
Water	56.6	51.9
R.m.s. deviations		
Bond lengths (Å)	0.003	0.003
Bond angles (˚)	0.64	0.67
Ramachandran (%)		
Favored	94.48	95.89
Outliers	0.42	0.32

[a]Values in parentheses are for highest-resolution shell

Supplementary Table 4. NMR assignments for 7 (^{1}H for 600 MHz and ^{13}C for 150 MHz in DMSO-d_6).

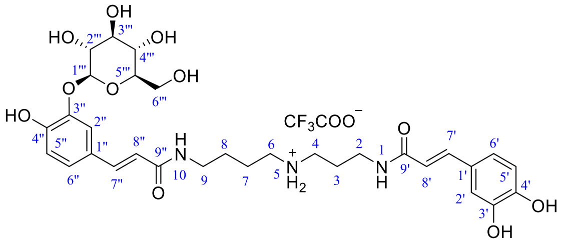

Position	δ_C, type	δ_H (J in Hz)[a]	^{1}H–^{1}H COSY	HMBC	ROESY
1		8.13/8.19	2		
2	35.8, CH_2	3.23	1, 3		
3	26.3, CH_2	1.77	2, 4		
4	44.8, CH_2	2.91, br s	3		
5					
6	46.6, CH_2	2.91, br s	7		
7	23.2, CH_2	1.59	6, 8		
8	26.4, CH_2	1.50	7, 9	7, 9	
9	37.9, CH_2	3.18	8, 10	7, 8, 9''	
10		8.05/7.99	9		
1'	126.2/126.3, C				
2'	113.8, CH	6.94		3', 4', 6', 7'	7', 8'
3'	145.5/145.6, C				
4'	147.3/147.4, C				
5'	115.7, CH	6.74, d (7.8)	6'	1', 3', 4'	
6'	120.3/120.5, CH	6.83, d (7.8)	5'	1', 2', 4', 7'	7', 8'
7'	139.5, CH	7.25, br d (15.6)	8'	1', 2', 6', 8', 9'	2', 6'
8'	118.0, CH	6.31, d (15.0)	7'	1', 9'	2', 6'
9'	165.8/165.9, C				
1''	126.4/126.5, C				
2''	115.7, CH	7.34 br s		3'', 4'', 6'', 7''	8'', 1'''
3''	145.5, C				
4''	148.4/148.5, C				
5''	116.2, CH	6.83, d (7.8)	6''	1'', 3'', 4''	
6''	122.8, CH	7.10, br t (7.2)	5''	2'', 4'', 5'', 7''	7'', 8''
7''	138.9, CH	7.29, br d (15.0)	8''	1'', 2'', 6'', 8'', 9''	6''
8''	119.2, CH	6.40, br d (15.0)	7''	1'', 9''	2'', 6''
9''	165.3/165.4, C				
1'''	102.1, CH	4.73, br d (5.4)	2'''	3'', 3''', 5'''	2'', 3''', 5'''
2'''	73.3, CH	3.30	1''', 3'''	1''', 3'''	
3'''	76.0, CH	3.28	2''', 4'''	2''', 4'''	1'''
4'''	69.8, CH	3.18	3''', 5'''	3''', 5'''	6'''a, 6'''b

5'''	77.3, CH	3.36	4''', 6'''a, 6'''b	1''', 6'''	1'''
6'''	60.7, CH_2	a: 3.48	5''', 6'''b	5'''	4'''
		b: 3.76	5''', 6'''a		4'''

[a] The indiscernible signals from the complex multiplicity are reported without designating multiplicity.

Supplementary Table 5. NMR assignments for 8 (^{1}H for 600 MHz and ^{13}C for 150 MHz in DMSO-d_6).

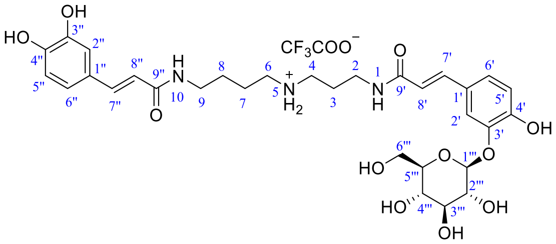

Position	δ_C, type	δ_H (J in Hz)[a]	^{1}H–^{1}H COSY	HMBC	ROESY
1		8.13/8.19	2		
2	35.8, CH_2	3.23	1, 3		
3	26.3, CH_2	1.77	2, 4		
4	44.8, CH_2	2.91, br s	3		
5					
6	46.6, CH_2	2.91, br s	7		
7	23.2, CH_2	1.59	6, 8		
8	26.4, CH_2	1.50	7, 9	7, 9	
9	37.9, CH_2	3.18	8, 10	7, 8, 9''	
10		8.05/7.99	9		
1'	126.4/126.5, C				
2'	115.7, CH	7.34 br s		3', 4', 6', 7'	8', 1'''
3'	145.5, C				
4'	148.4/148.5, C				
5'	116.2, CH	6.83, d (7.8)	6'	1', 3', 4'	
6'	122.8, CH	7.10, br t (7.2)	5'	2', 4', 5', 7'	7', 8'
7'	139.2, CH	7.31, br d (15.0)	8'	1', 2', 6', 9'	6'
8'	118.8, CH	6.40, br d (15.0)	7'	1'	2', 6'
9'	165.8/165.9, C				
1''	126.2/126.3, C				
2''	113.8, CH	6.94		3'', 4'', 6''	7'', 8''
3''	145.5/145.6, C				
4''	147.3/147.4, C				
5''	115.7, CH	6.74, d (7.8)	6''	1'', 3'', 4''	
6''	120.3/120.5, CH	6.83, d (7.8)	5''	1'', 2'', 4'', 7''	7'', 8''
7''	139.1, CH	7.23, br d (15.6)	8''	1'', 2'', 6'', 8'', 9''	2'', 6''
8''	118.4, CH	6.32, d (15.0)	7''	1''	2'', 6''
9''	165.3/165.4, C				
1'''	102.1, CH	4.73, br d (5.4)	2'''	3', 3''', 5'''	2', 3''', 5'''
2'''	73.3, CH	3.30	1''', 3'''	1''', 3'''	
3'''	76.0, CH	3.28	2''', 4'''	2''', 4'''	1'''
4'''	69.8, CH	3.18	3''', 5'''	3''', 5'''	6'''a, 6'''b

5'''	77.3, CH	3.36	4''', 6'''a, 6'''b	1''', 6'''	1'''
6'''	60.7, CH_2	a: 3.48	5''', 6'''b	5'''	4'''
		b: 3.76	5''', 6'''a		4'''

[a] The indiscernible signals from the complex multiplicity are reported without designating multiplicity.

Supplementary Table 6. NMR assignments for 9 (^{1}H for 400 MHz and ^{13}C for 100 MHz in DMSO-d_6).

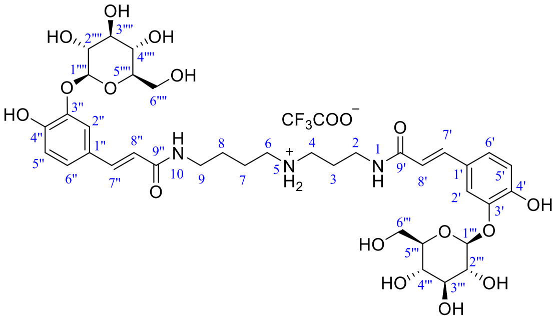

Position	δ_C, type	δ_H (J in Hz) [a]	^{1}H–^{1}H COSY	HMBC	ROESY
1		8.14, t (5.2)	2	9'	3, 8'
2	35.8, CH_2	3.25	1, 3	4, 9'	
3	26.2, CH_2	1.78, quint (6.8)	2, 4	2, 4	1
4	44.8, CH_2	2.91, br s	3, 5		
5		8.48, br s	4, 6		
6	46.6, CH_2	2.91, br s	5, 7		
7	23.2, CH_2	1.59, quint (6.0)	6, 8	6, 8	
8	26.4, CH_2	1.51, quint (6.4)	7, 9	6, 7, 9	10
9	37.9, CH_2	3.18	8, 10		
10		8.00, t (5.1)	9	9''	8, 8''
1'	126.6, C				
2'	115.8, CH	7.34		1', 3', 4', 6', 7'	7', 8', 1'''
3'	145.5, C				
4'	148.5, C				
5'	116.2, CH	6.84, d (8.4)	6'	1', 3', 4'	
6'	123.0, CH	7.10, br t (6.8)	5'	2', 4', 7', 5'	7'
7'	139.2, CH	7.31	8'	1', 2', 6', 8', 9'	2', 6'
8'	118.8, CH	6.38	7'	1', 9'	1, 2', 6'
9'	165.9, C				
1''	126.4, C				
2''	115.8, CH	7.34		1'', 3'', 4'', 6'', 7''	7'', 8'', 1''''
3''	145.5, C				
4''	148.4, C				
5''	116.2, CH	6.84, d (8.4)	6''	1'', 3'', 4''	
6''	122.9, CH	7.10, br t (6.8)	5''	2'', 4'', 7'', 5''	5'', 7''
7''	138.9, CH	7.27, br d (15.7)	8''	1'', 2'', 6'', 8'', 9''	2'', 6''
8''	119.2, CH	6.42	7''	1'', 9''	10, 2'', 6''
9''	165.4, C				
1'''	102.1, CH	4.74, d (6.0)	2'''	3'	2', 3''', 5'''
2'''	73.3, CH	3.30	1''', 3'''	1''', 3'''	

3'''	76.0, CH	3.25	2''', 4'''	2''', 4'''	1'''
4'''	69.9, CH	3.18	3''', 5'''	3''', 5''', 6'''	
5'''	77.3, CH	3.36	4''', 6'''a, 6'''b	1''', 6'''	1'''
6'''	60.8, CH_2	a: 3.49, dd (11.8, 5.9)	5''', 6'''b	5'''	
		b: 3.75, br d (11.2)	5''', 6'''a	4'''	
1''''	102.1, CH	4.74, d (6.0)	2''''	3''	2'', 3'''', 5''''
2''''	73.3, CH	3.30	1'''', 3''''	1'''', 3''''	
3''''	76.0, CH	3.25	2'''', 4''''	2'''', 4''''	1''''
4''''	69.9, CH	3.18	3'''', 5''''	3'''', 5'''', 6''''	
5''''	77.3, CH	3.36	4'''', 6''''a, 6''''b	1'''', 6''''	1''''
6''''	60.8, CH_2	a: 3.49, dd (11.8, 5.9)	5'''', 6''''b	5''''	
		b: 3.75, br d (12.7)	5'''', 6''''a	4''''	

[a] The indiscernible signals from the complex multiplicity are reported without designating multiplicity.

Supplementary Table 7. NMR assignments for 15 (^{1}H for 400 MHz and ^{13}C for 100 MHz in DMSO-d_6).

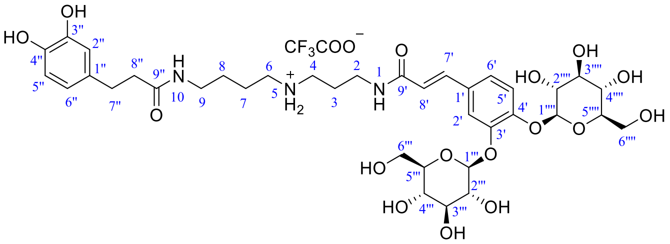

Position	δ_C, type	δ_H (*J* in Hz)[a]	^{1}H–^{1}H COSY	HMBC	ROESY
1		8.32, br s	2		
2	36.1, CH_2	3.25	1, 3		
3	26.4, CH_2	1.78, br s	2, 4		
4	44.8, CH_2	2.86, br s	3, 5		
5		8.32, br s	4, 6		
6	46.6, CH_2	2.84, br s	5, 7		
7	23.4, CH_2	1.51, br s	6, 8		
8	26.4, CH_2	1.40, br s	7, 9	6, 7, 9	
9	37.8, CH_2	3.02, br s	8, 10	7, 8, 9''	
10		7.86, br s	9	9''	
1'	129.4, C				
2'	116.5, CH	7.38, br s		1', 3', 4', 6', 7'	6', 8', 1'''
3'	147.2, C				
4'	148.4, C				
5'	117.4, CH	7.16, br d (8.4)	6'	1', 3', 4', 6'	1''''
6'	122.7, CH	7.18, br d (8.4)	5'	2', 4', 5', 7'	2', 7'
7'	138.7, CH	7.33, br s	8'	1', 2', 6', 8', 9'	6'
8'	120.5, CH	6.48, br d (15.6)	7'	1', 9'	2'
9'	165.6, C				
1''	132.1, C				
2''	115.5, CH	6.56, s		3'', 4'', 6'', 7''	
3''	145.1, C				
4''	143.5, C				
5''	115.8, CH	6.59, d (8.0)	6''	1'', 3'', 4'', 6''	
6''	118.7, CH	6.40, d (8.0)	5''	4'', 5'', 7''	7''
7''	30.7, CH_2	2.61, br t (7.6)	8''	1'', 2'', 6'', 8'', 9''	6''
8''	37.6, CH_2	2.25, br t (7.2)	7''	1'', 7'', 9''	
9''	171.6, C				
1'''	101.0, CH	4.85, br d (8.0)	2'''	3', 3'''	2', 3''', 5'''
2'''	73.3, CH	3.30	1''', 3'''	1''', 3''', 4'''	
3'''	76.6, CH	3.27	2''', 4'''	2''', 4'''	1'''
4'''	69.7, CH	3.18	3''', 5'''	3''', 5''', 6'''	6'''a, 6'''b

5'''	77.1, CH	3.30	4''', 6'''a, 6'''b	3''', 4''', 6'''	1'''
6'''	60.7, CH_2	a: 3.47	5''', 6'''b	4''', 5''',	4'''
		b: 3.69, br t (12.0)	5''', 6'''a	4'''	4'''
1''''	101.4, CH	4.87, br d (8.0)	2''''	4', 3''''	5', 3'''', 5''''
2''''	73.4, CH	3.29	1'''', 3''''	1'''', 3'''', 4''''	
3''''	76.4, CH	3.27	2'''', 4''''	2'''', 4''''	1''''
4''''	69.8, CH	3.18	3'''', 5''''	3'''', 5'''', 6''''	6''''a, 6''''b
5''''	77.2, CH	3.31	4'''', 6''''a, 6''''b	3'''', 4'''', 6''''	1''''
6''''	60.8, CH_2	a: 3.47	5'''', 6''''b	4'''', 5''''	4''''
		b: 3.69, br t (12.0)	5'''', 6''''a	4''''	4''''

[a] The indiscernible signals from the complex multiplicity are reported without designating multiplicity.

Supplementary Table 8. NMR assignments for 16 (^{1}H for 600 MHz and ^{13}C for 150 MHz in DMSO-d_6).

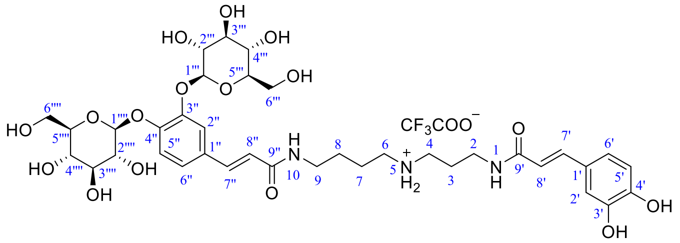

Position	δ_C, type	δ_H (J in Hz) [a]	^{1}H–^{1}H COSY	HMBC	ROESY
1		8.20, t (5.4)	2	9'	3, 8'
2	35.7, CH_2	3.23	1, 3	3, 4, 9'	4
3	26.2, CH_2	1.77	2, 4	2, 4	1, 5
4	44.8, CH_2	2.91, br s	3, 5		2
5		8.41, br s	4, 6		3, 7
6	46.6, CH_2	2.91, br s	5, 7		8
7	23.2, CH_2	1.59	6, 8	6, 8	5, 9
8	26.4, CH_2	1.51	7, 9	7, 9	6, 10
9	38.0, CH_2	3.19	8, 10	8	
10		8.07, t (5.1)	9	9''	8, 8''
1'	126.2, C				
2'	113.8, CH	6.95		3', 4', 6', 7'	7', 8'
3'	145.6, C				
4'	147.1, C				
5'	115.7, CH	6.75, d (7.8)	6'	1', 3', 4'	
6'	120.7, CH	6.84, d (7.8)	5'	2', 4', 7'	7', 8'
7'	139.5, CH	7.26	8'	1', 2', 6', 8', 9'	2', 6'
8'	118.0, CH	6.31, d (15.6)	7'	1', 9'	1, 2', 6'
9'	166.0, C				
1''	129.4, C				
2''	116.7, CH	7.35		1'', 3'', 4'', 6'', 7''	8'', 1'''
3''	147.1, C				
4''	148.3, C				
5''	117.4, CH	7.16	6''	1'', 3'', 4''	1''''
6''	122.3, CH	7.18	5''	2'', 4'', 7''	8''
7''	138.4, CH	7.33	8''	1'', 2'', 6'', 8'', 9''	
8''	120.5, CH	6.49, d (15.6)	7''	1'', 9''	10, 2'', 6''
9''	165.2, C				
1'''	100.9, CH	4.86, br d (6.6)	2'''	3''	2'', 3''', 5'''
2'''	73.3, CH	3.30	1''', 3'''	1''', 3'''	
3'''	76.4, CH	3.28	2''', 4'''	2''', 4'''	1'''
4'''	69.7, CH	3.18	3''', 5'''	3''', 6'''	

5'''	77.1, CH	3.31	4''', 6'''a, 6'''b	3'''	1'''
6'''	60.7, CH_2	a: 3.48	5''', 6'''b		
		b: 3.69, br t (12.0)	5''', 6'''a		
1''''	101.3, CH	4.87, br d (6.0)	2''''	4''	5'', 3'''', 5''''
2''''	73.3, CH	3.29	1'''', 3''''	1'''', 3''''	
3''''	76.5, CH	3.28	2'''', 4''''	2'''', 4''''	1''''
4''''	69.6, CH	3.18	3'''', 5''''	3'''', 6''''	
5''''	77.1, CH	3.31	4'''', 6''''a, 6''''b	3''''	1''''
6''''	60.7, CH_2	a: 3.48	5'''', 6''''b		
		b: 3.69, br t (12.7)	5'''', 6''''a		

[a] The indiscernible signals from the complex multiplicity are reported without designating multiplicity.

Supplementary Table 9. NMR assignments for 17 (^{1}H for 600 MHz and ^{13}C for 150 MHz in DMSO-d_6).

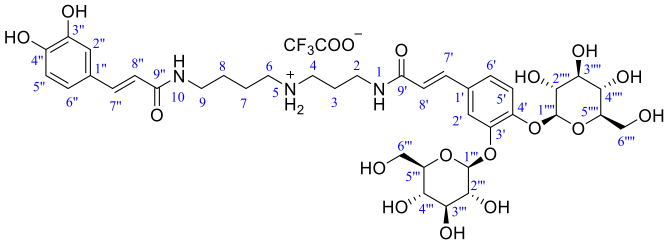

Position	δ_C, type	δ_H (J in Hz)[a]	^{1}H–^{1}H COSY	HMBC	ROESY
1		8.21, t (5.9)	2	2, 9'	3, 8'
2	35.9, CH_2	3.25	1, 3	3, 4, 9'	
3	26.2, CH_2	1.78, quint (6.9)	2, 4	2, 4	1, 5
4	44.8, CH_2	2.91, br s	3, 5		
5		8.42, br s	4, 6		3, 7
6	46.6, CH_2	2.91, br s	5, 7		8
7	23.2, CH_2	1.59, quint (7.1)	6, 8	6, 8, 9	5, 9
8	26.4, CH_2	1.49, quint (7.0)	7, 9	6, 7, 9	6, 10
9	37.9, CH_2	3.18	8, 10	7, 8, 9''	7
10		8.06, t (5.9)	9	9, 9''	8, 8''
1'	129.3, C				
2'	116.7, CH	7.36, br d (6.0)		1', 3', 4', 6', 7'	6', 8', 1'''
3'	147.3, C				
4'	148.4, C				
5'	117.4, CH	7.16	6'	1', 3'	1''''
6'	122.5, CH	7.20	5'	2', 4'	2', 8'
7'	138.8, CH	7.37, d (12.0)	8'	1', 2', 6', 8', 9'	
8'	120.3, CH	6.49, d (15.8)	7'	1', 9'	1, 2', 6'
9'	165.7, C				
1''	126.3, C				
2''	113.8, CH	6.93, d (1.2)		1'', 3'', 4'', 6'', 7''	8''
3''	145.6, C				
4''	147.1, C				
5''	115.8, CH	6.74, d (8.1)	6''	1'', 3'', 4''	
6''	120.4, CH	6.83, dd (8.2, 1.8)	5''	2'', 4'', 7''	7'', 8''
7''	139.2, CH	7.24	8''	1'', 2'', 6'', 8'', 9''	6''
8''	118.4, CH	6.32, d (15.2)	7''	1'', 9''	10, 2'', 6''
9''	165.5, C				
1'''	100.9, CH	4.86, br d (6.6)	2'''	3', 3'''	2', 3''', 5'''
2'''	73.4, CH	3.30	1''', 3'''	1''', 3''', 4'''	
3'''	76.4, CH	3.27	2''', 4'''	2''', 4'''	1'''
4'''	69.7, CH	3.18	3''', 5'''	3''', 5''', 6'''	6'''a, 6'''b

5'''	77.1, CH	3.30	4''', 6'''a, 6'''b	3''', 4''', 6'''	1'''
6'''	60.7, CH_2	a: 3.48	5''', 6'''b	4''', 5'''	4'''
		b: 3.69 br t (12.0)	5''', 6'''a	4'''	4'''
1''''	101.3, CH	4.87, br d (6.6)	2''''	4', 3''''	5', 3'''', 5''''
2''''	73.3, CH	3.29	1'''', 3''''	1'''', 3'''', 4''''	
3''''	76.5, CH	3.27	2'''', 4''''	2'''', 4''''	1''''
4''''	69.6, CH	3.18	3'''', 5''''	3'''', 5'''', 6''''	6''''a, 6''''b
5''''	77.1, CH	3.30	4'''', 6''''a, 6''''b	3'''', 4'''', 6''''	1''''
6''''	60.7, CH_2	a: 3.48	5'''', 6''''b	4'''', 5''''	4''''
		b: 3.69, br t (12.0)	5'''', 6''''a	4''''	4''''

[a] The indiscernible signals from the complex multiplicity are reported without designating multiplicity.

Supplementary Table 10. Primers used for constructing recombinant plasmids.

Primer	Sequence (5' to 3')	Usage
Inf-LbUGT1-F	tccccgggaattcatATGACAACGAAAGCAGAGCT	Cloning of LbUGT1 from *L. barbarum* L. cDNA.
Inf-LbUGT1-R	agtcacgatgaattcTCAAGAAATGTTACTAATGACATCCTC	
Inf-LbUGT2-F	tccccgggaattcatATGGGACAGCTCCATTTTTT	Cloning of LbUGT2 from *L. barbarum* L. cDNA.
Inf-LbUGT2-R	agtcacgatgaattcTCAATCACTACTTAATGACT	
Inf-LbUGT3-F	tccccgggaattcatATGAAAGAAACCAAGAACATAG	Cloning of LbUGT3 from *L. barbarum* L. cDNA.
Inf-LbUGT3-R	agtcacgatgaattcTTACCGAGTATTCTCCATGAT	
Inf-LbUGT4-F	tccccgggaattcatATGAAAGAATTAATTTTCATTCC	Cloning of LbUGT4 from *L. barbarum* L. cDNA.
Inf-LbUGT4-R	agtcacgatgaattcCTAAATATTAGTGATTACATCCTTAATTAG	
Inf-LbUGT5-F	tccccgggaattcatATGAGCAAATTAGAGCTGGT	Cloning of LbUGT5 from *L. barbarum* L. cDNA.
Inf-LbUGT5-R	agtcacgatgaattcCTAGGAATCAAGAATAGTTTCAACA	
Inf-LbUGT6-F	tccccgggaattcatATGGCAGAAACACCTGTT	Cloning of LbUGT6 from *L. barbarum* L. cDNA.
Inf-LbUGT6-R	agtcacgatgaattcTCAATTATTTGACACTTTTTTCTTCAAT	
Inf-LbUGT7-F	tccccgggaattcatATGGAGAGAACAGAGTTGAT	Cloning of LbUGT7 from *L. barbarum* L. cDNA.
Inf-LbUGT7-R	agtcacgatgaattcTCAATTGGACGAATGATCCT	
Inf-LbUGT8-F	tccccgggaattcatATGGAGACAAAAGCAGAACTAG	Cloning of LbUGT8 from *L. barbarum* L. cDNA.
Inf-LbUGT8-R	agtcacgatgaattcTTATCGCGCCTTCAATTTCC	
Inf-LbUGT9-F	tccccgggaattcatATGAAGAGTGCAGAGTTAGT	Cloning of LbUGT9 from *L. barbarum* L. cDNA.
Inf-LbUGT9-R	agtcacgatgaattcTTAGATCTTCATGACAGTCTCAAC	
Inf-LbUGT10-F	tccccgggaattcatATGAAGAGAGTAGAGTTAGTATTCA	Cloning of LbUGT10 from *L. barbarum* L. cDNA.
Inf-LbUGT10-R	agtcacgatgaattcTTAGCTCTTCATGACAGTCT	
Inf-LbUGT11-F	tccccgggaattcatATGGCTGGCTCACAGGAACT	Cloning of LbUGT11 from *L. barbarum* L. cDNA.
Inf-LbUGT11-R	agtcacgatgaattcTTAGGAAGTTTCCTCAGACATGTT	
Inf-LbUGT12-F	tccccgggaattcatATGGCTATAGCAGAAGAAATC	Cloning of LbUGT12 from *L. barbarum* L. cDNA.
Inf-LbUGT12-R	agtcacgatgaattcTCACTGAGATTTCCAAAACTG	
Inf-LbUGT13-F	tccccgggaattcatATGCATCAACTCCATTTTTTC	Cloning of LbUGT13 from *L. barbarum* L. cDNA.
Inf-LbUGT13-R	agtcacgatgaattcCTAATTACTTGTAAAACTATACG	
Inf-LbUGT14-F	tccccgggaattcatATGGGTCAGCTCCATTTTTT	Cloning of LbUGT14 from *L. barbarum* L. cDNA.
Inf-LbUGT14-R	agtcacgatgaattcTTAATGACTTGTAGAACTATATGTAC	
Inf-LbUGT15-F	tccccgggaattcatATGCATGAAAAAACTGATCAAC	Cloning of LbUGT15 from *L. barbarum* L. cDNA.
Inf-LbUGT15-R	agtcacgatgaattcTCAGTTGGAGCTGCTCTTC	
Inf-LbUGT16-F	tccccgggaattcatATGGATCACAATCAACTACACG	Cloning of LbUGT16 from *L. barbarum* L. cDNA.
Inf-LbUGT16-R	agtcacgatgaattcTTAAGTCGATGAGGAAGAAGCAA	
Inf-LbUGT17-F	tccccgggaattcatATGGATAGAAAAACTGATAAACTTC	Cloning of LbUGT17 from *L. barbarum* L. cDNA.
Inf-LbUGT17-R	agtcacgatgaattcTCAGTTGGAGCTGCTTTTCC	
Inf-LbUGT18-F	tccccgggaattcatATGCTTGAAAAAACTGATCAACTTC	Cloning of LbUGT18 from *L. barbarum* L. cDNA.
Inf-LbUGT18-R	agtcacgatgaattcTCAGTTGGAGCTGCTCTTCC	
Inf-LbUGT19-F	tccccgggaattcatATGGATCGCACTCAACTACACG	Cloning of LbUGT19 from *L. barbarum* L. cDNA.
Inf-LbUGT19-R	agtcacgatgaattcTTAAGTCCATGAGTAAGAAGCAA	
Inf-LbUGT20-F	tccccgggaattcatATGCATCAACTCCATTATTAC	Cloning of LbUGT20 from *L. barbarum* L. cDNA.
Inf-LbUGT20-R	agtcacgatgaattcCTAACTACTTGTAATACTATACG	
GEX-F	cacgtttggtggtggcgac	DNA sequencing of recombinant pGEX-2TK plasmids.
GEX-R	cttgtctgctcccggcatcc	
H17A(UGT1)-F	TTCCTTCACCTGGAGCAGGTGCCCTTATCTCCGCCATTGAA A	Construction of H17A mutant plasmid of LbUGT1.
H17A(UGT1)-R	ACCTGCTCCAGGTGAAGGAA	
E83A(UTG1)-F	TGGATTGTTGAAAAATAAGGCATCCTTCTTCACTGACTTTA TTC	Construction of E83A mutant plasmid of LbUGT1.
E83A(UTG1)-R	CCTTATTTTTCAACAATCCAGC	
T144A(UTG1)-F	TTCCGAGTTATATTTTCCTCGCTTCAAATGCTGCTTTTCTT	Construction of T144A mutant plasmid of LbUGT1.
T144A(UGT1)-R	GAGGAAAATATAACTCGGAACAC	
D197A(UTG1)-F	CTTGCCTAGATTGACAACAGCTCAAACAACCGCGACGACC	Construction of D197A mutant plasmid of LbUGT1.
D197A(UTG1)-R	CTGTTGTCAATCTAGGCAAG	
Q352A(UGT1)-F	AAGTTATAGGATGGGCACCTGCAGTGGCAGTACTATCTCA C	Construction of Q352A mutant plasmid of LbUGT1.

Q352A(UGT1)-R	AGGTGCCCATCCTATAACTT	
H367A(UGT1)-F	CAGTGGGTGGATTTGTATCTGCTTGTGGATGGAATTCTGTGC	Construction of H367A mutant plasmid of LbUGT1.
H367A(UGT1)-R	AGATACAAATCCACCCACTG	
N371A(UGT1)-F	TTGTATCTCATTGTGGATGGGCTTCTGTGCTTGAAAGCTTGT	Construction of N371A mutant plasmid of LbUGT1.
N371A(UGT1)-R	CCATCCACAATGAGATACAA	
S372A(UGT1)-F	TATCTCATTGTGGATGGAATGCTGTGCTTGAAAGCTTGTGT	Construction of S372A mutant plasmid of LbUGT1.
S372A(UGT1)-R	ATTCCATCCACAATGAGATA	
E375A(UGT1)-F	TGGATGGAATTCTGTGCTTGCAAGCTTGTGTTTTGGAGTCC	Construction of E375A mutant plasmid of LbUGT1.
E375A(UGT1)-R	CAAGCACAGAATTCCATCCA	
Y389A(UGT1)-F	CAATAGGCGCTTGGCCACAGGCCGCGGAACAGCAGATGAATG	Construction of Y389A mutant plasmid of LbUGT1.
Y389A(UGT1)-R	CTGTGGCCAAGCGCCTATTG	
Y389F(UGT1)-F	CAATAGGCGCTTGGCCACAGTTCGCGGAACAGCAGATGAATG	Construction of Y389F mutant plasmid of LbUGT1.
Y389F(UGT1)-R	CTGTGGCCAAGCGCCTATTG	
A390F(UGT1)-F	TAGGCGCTTGGCCACAGTACTTCGAACAGCAGATGAATGCCTT	Construction of A390F mutant plasmid of LbUGT1.
A390F(UGT1)-R	GTACTGTGGCCAAGCGCCTA	
A390D(UGT1)-F	TAGGCGCTTGGCCACAGTACGATGAACAGCAGATGAATGCCTT	Construction of A390D mutant plasmid of LbUGT1.
A390D(UGT1)-R	GTACTGTGGCCAAGCGCCTA	
E391A(UGT1)-F	CGCTTGGCCACAGTACGCGGCACAGCAGATGAATGCCTTCG	Construction of E391A mutant plasmid of LbUGT1.
E391A(UGT1)-R	CCGCGTACTGTGGCCAAGCG	
Q392A(UGT1)-F	CTTGGCCACAGTACGCGGAAGCGCAGATGAATGCCTTCGAGT	Construction of Q392A mutant plasmid of LbUGT1.
Q392A(UGT1)-R	TTCCGCGTACTGTGGCCAAG	
D416A(UGT1)-F	TCGAATGGACTATTTCAAGGCCTTCGAAGGGAAACATGGGC	Construction of D416A mutant plasmid of LbUGT1.
D416A(UGT1)-R	CCTTGAAATAGTCCATTCGA	
E418A(UGT1)-F	GGACTATTTCAAGGACTTCGCAGGGAAACATGGGCCTGTC	Construction of E418A mutant plasmid of LbUGT1.
E418A(UGT1)-R	CGAAGTCCTTGAAATAGTCC	
F382A(UGT2)-F	CGATGGTGACGTGGCCAGTGGCTGCTGAGCAATTTTTCAATG	Construction of F382A mutant plasmid of LbUGT2.
F382A(UGT2)-R	CACTGGCCACGTCACCATCG	
G16A (UGT3)-F	TGATCTTCATTCCCTGTCCAGCAATTGGTCATTTAGTATCCACAG	Construction of G16A mutant plasmid of LbUGT3.
G16A (UGT3)-R	TGGACAGGGAATGAAGATCA	
G16L (UGT3)-F	TGATCTTCATTCCCTGTCCACTAATTGGTCATTTAGTATCCACAG	Construction of G16L mutant plasmid of LbUGT3.
G16L (UGT3)-R	TGGACAGGGAATGAAGATCA	
H19A(UGT3)-F	TTCCCTGTCCAGGAATTGGTGCTTTAGTATCCACAGTAGAAATGG	Construction of H19A mutant plasmid of LbUGT3.
H19A(UGT3)-R	ACCAATTCCTGGACAGGGAA	
R321A(UGT3)-F	AACCACCAGAGAAAGATGCAGCGTTTCCAAGTGACTATGAAAATTTTGAAG	Construction of R321A mutant plasmid of LbUGT3.
R321A(UGT3)-R	TGCATCTTTCTCTGGTGGTT	
Y390A(UGT3)-F	CAATGGCTACTTGGCCAATGGCCGCAGAGCAACAAGGGAATG	Construction of Y390A mutant plasmid of LbUGT3.
Y390A(UGT3)-R	CATTGGCCAAGTAGCCATTG	
Y390F(UGT3)-F	CAATGGCTACTTGGCCAATGTTCGCAGAGCAACAAGGGAATG	Construction of Y390F mutant plasmid of LbUGT3.
Y390F(UGT3)-R	CATTGGCCAAGTAGCCATTG	
Y388A(UGT4)-F	CATTCATAGCTTGGCCACTCGCTGCAGAACAAAAAATGAATG	Construction of Y388A mutant plasmid of LbUGT4.
Y388A(UGT4)-R	GAGTGGCCAAGCTATGAATG	
Y384A(UGT5)-F	CAATTGTGACATGGCCCCTTGCTGCAGAACAACATATCAATGC	Construction of Y384A mutant plasmid of LbUGT5.
Y384A(UGT5)-R	AAGGGGCCATGTCACAATTG	

Supplementary Table 11. Newly predicted DNA and protein sequences involved in this study..

Gene	Predicted DNA sequence	Predicted protein sequence
LbUGT1	ATGACAACGAAAGCAGAGCTTGTTTTCATTCCTTCACCTGGAGCA<br>GGTCACCTTATCTCCGCCATTGAAATTGCAAAACTCATTCTCAACA<br>GAGATGAAAGACTTTGCATTTCTGTCCTCATCATGAAGCTTCCAAT<br>GGATTTCGGCGTTCAATCCTACGTCGCATCACTTTCTTCTTCACCT<br>CGCTTGCAGTTCGTCGATATCACCCTTGACGAGAAAACTGTTGCT<br>GGATTGTTGAAAAATAAGGAATCCTTCTTCACTGACTTTATTCAAG<br>GTCACAAACCAAAAGTAAAAGATTTTATTCATAACACAAGCTTTT<br>CTAAATCCGGTTCGGGGTTTCTCGGCTTGCAGGCTTTGTTCTTGA<br>CATGTTCTGTACCTCTATGATTGATGTGGCAAGCGAATTTAGTGTT<br>CCGAGTTATATTTTCCTCACTTCAAATGCTGCTTTTCTTGCACTTTG<br>TTATCATTTTGAGTCCCTTAAAAAAGAGCATCATATAGATACCTCTA<br>AGTACAAGGACTCTGATGAAGAGTTAACAATCCCTGGTTTTAAAA<br>ACCCGTATCCAGGGAAATTCTTGCCTAGATTGACAACAGATCAAA<br>CAACCGCGACGACCTTATTCTTCGATTCTGTAACTCGATTCAAAG<br>AGACGAAGGGTATTATGGTCAACACATTCGCGGAGCTTGAACCTT<br>TTGCTCTTCAGTCTCTTTTAGTTCCGAAAATATATCCAGTTGGTCCT<br>GTGGTGAATTTTAAAGAAGGGGGTCACGGTCGAAACAGCGAATC<br>GGAAACAGAAAGTATTATCAAATGGTTGGATGATCAGCCAGAATC<br>CTCTGTAGTGTTTTTGTGCTTTGGAAGTATGGGAAGTTTTGATGCC<br>GAACAGATCGAAGAAATAGCAGTTGCATTGGAGTGCAGCGGTCA<br>CAGGTTCTTGTGGTCCTTACGAAGACCTCCACCGAAGGGGAAAA<br>TGGAGCTGCCGAGTAATTACGAAGATTTTCAGGAAGTGCTACCAG<br>AAGGGTTTATTGAAAGGACAAATGGGGTCGGGAAAGTTATAGGAT<br>GGGCACCTCAAGTGGCAGTACTATCTCACCCTGCAGTGGGTGGAT<br>TTGTATCTCATTGTGGATGGAATTCTGTGCTTGAAAGCTTGTGTTT<br>TGGAGTCCCAATAGGCGCTTGGCCACAGTACGCGGAACAGCAGA<br>TGAATGCCTTCGAGTTGGTTAAACAATTGGGGCTGGCAGTGGAGA<br>TTCGAATGGACTATTTCAAGGACTTCGAAGGGAAACATGGGCCTG<br>TCGAGATTGTTACTGCGAAGGAAATTGCAAGTGGCATACGACAGC<br>TGATGGCGGATGGCGAGGAGAATGAAATCAGGAAAAGGGCGAA<br>GGAAATGAAAGAGAAAAGCAGTGCAGCCATGAAGGAGGGTGGT<br>TCATCTTATGCATCTCTTGGGCTTCTAATTGAGGATGTCATTAGTAA<br>CATTTCTTGA	MTTKAELVFIPSPGAGHL<br>ISAIEIAKLILNRDERLCIS<br>VLIMKLPMDFGVQSYVA<br>SLSSSPRLQFVDITLDEK<br>TVAGLLKNKESFFTDFIQ<br>GHKPKVKDFIHNTSFSKS<br>GSGFSRLAGFVLDMFCT<br>SMIDVASEFSVPSYIFLTS<br>NAAFLALCYHFESLKKE<br>HHIDTSKYKDSDEELTIP<br>GFKNPYPGKFLPRLTTD<br>QTTATTLFFDSVTRFKET<br>KGIMVNTFAELEPFALQS<br>LLVPKIYPVGPVVNFKEG<br>GHGRNSESETESIIKWLD<br>DQPESSVVFLCFGSMGS<br>FDAEQIEEIAVALECSGH<br>RFLWSLRRPPPKGKMEL<br>PSNYEDFQEVLPEGFIER<br>TNGVGKVIGWAPQVAVL<br>SHPAVGGFVSHCGWNSV<br>LESLCFGVPIGAWPQYAE<br>QQMNAFELVKQLGLAVE<br>IRMDYFKDFEGKHGPVE<br>IVTAKEIASGIRQLMADG<br>EENEIRKRAKEMKEKSS<br>AAMKEGGSSYASLGLLI<br>EDVISNIS*
LbUGT2	ATGGGACAGCTCCATTTTTTCCTCTTTCCCATGATGGCTCAAGGCC<br>ACATGATTCCTACACTTGACATGGCCAAGCTCATCGCTTCTCGTGG<br>TGTTAAGGCCACTATAATCACTACCCCTCTCAATGAATCCGTTTTC<br>TCCAAAGCAATTCAAAGAAACAAACAGTTGGGTATCGAAATCGA<br>AATCGAAATCCGTTTGATAAAATTCCCAGCTTTGGAGAATGACTT<br>GCCTGAAGATTGCGAGCGACTTGATCTCATCCCTACTGAAGCCCA	MGQLHFFLFPMMAQGH<br>MIPTLDMAKLIASRGVK<br>ATIITTPLNESVFSKAIQR<br>NKQLGIEIEIEIRLIKFPAL<br>ENDLPEDCERLDLIPTEA<br>HLPNFFKAAAMMQEPLE

	TCTTCCCAACTTCTTCAAAGCTGCAGCTATGATGCAAGAACCATT<br>AGAGCAGCTAATTCAAGAATGTCGCCCTGATTGTCTTGTTTCTGAT<br>ATGTTCCTTCCTTGGACAACTGATACTGCAGCTAAATTTAACATTC<br>CAAGAATTGTTTTCCATGGTACAAACTACTTTGCCCTTTGTGTTGG<br>AGACAGTATGAGGCGTAATAAGCCTTTCAAGAATGTCTCATCTGAT<br>TCTGAAACTTTTGTTGTACCGAATTTACCTCATGAAATCAAGCTGA<br>CTAGAACTCAGGTGTCTCCGTTTGAGCAATCGGATGAAGAGTCAG<br>TTATGTCTCGTGTGCTAAAAGAAGTCAGGGAATCGGATTTGAAGA<br>GCTATGGAGTTATCTTCAATAGTTTCTATGAGCTTGAACCAGATTAT<br>GTTGAACATTATACCAAGGTTATGGGTAGAAAATCTTGGGCTATTG<br>GCCCGCTTTCGTTGTGCAACAGGGACGTTGAAGATAAAGCTGAA<br>AGAGGGAAGAAATCCTCTATTGACAAACACGAGTGTTTGGAATG<br>GCTTGATTCGAAGAAACCTAGTTCGATTGTTTACGTTTGTTTTGGA<br>AGCGTCGCAAATTTTACTGTAACTCAGATGAGAGAACTTGCTTTG<br>GGACTCGAAGCTTCTGGACTGGATTTCATTTGGGCTGTTAGAGCA<br>GATAACGAAGATTGGTTGCCTGAAGGGTTCGAGGAAAGAACGAA<br>AGAAAAAGGATTAATTATAAGGGGATGGGCCCCACAAGTGCTGAT<br>TCTTGATCACGAATCTGTGGGAGCTTTTGTGACTCACTGTGGATG<br>GAATTCGACGCTTGAAGGAATATCGGCAGGGGTGCCGATGGTGAC<br>GTGGCCAGTGTTTGCTGAGCAATTTTTCAATGAAAAGTTGGTGAC<br>TCAGGTTATGAGAACCGGGGCTGGCGTCGGTTCGGTGCAATGGA<br>AGAGATCAGCCAGTGAAGGAGTGGAAAAAGAAGCAATCGCGAA<br>GGCGATAAAGAGAGTAATGGTGAGCGAAGAAGCAGAGGGATTTA<br>GAAACAGAGCTAGGGCGTATAAGGAAATGGCAAGACAAGCTATT<br>GAAGAAGGAGGATCATCTTACACTGGACTGACTACTTTGCTGGAA<br>GATATAAGTTCATACGAGTCATTAAGTAGTGATTGA	QLIQECRPDCLVSDMFLP<br>WTTDTAAKFNIPRIVFHG<br>TNYFALCVGDSMRRNKP<br>FKNVSSDSETFVVPNLPH<br>EIKLTRTQVSPFEQSDEES<br>VMSRVLKEVRESDLKSY<br>GVIFNSFYELEPDYVEHY<br>TKVMGRKSWAIGPLSLC<br>NRDVEDKAERGKKSSID<br>KHECLEWLDSKKPSSIV<br>YVCFGSVANFTVTQMRE<br>LALGLEASGLDFIWAVR<br>ADNEDWLPEGFEERTKE<br>KGLIIRGWAPQVLILDHE<br>SVGAFVTHCGWNSTLEG<br>ISAGVPMVTWPVFAEQF<br>FNEKLVTQVMRTGAGV<br>GSVQWKRSASEGVEKE<br>AIAKAIKRVMVSEEAEG<br>FRNRARAYKEMARQAIE<br>EGGSSYTGLTTLLEDISS<br>YESLSSD*
LbUGT3	ATGAAAGAAACCAAGAACATAGAGTTGATCTTCATTCCCTGTCCA<br>GGAATTGGTCATTTAGTATCCACAGTAGAAATGGCAAAACTTCTC<br>ATAACTAGAGAAAAACACATGTCCATAACAGTCCTCATTATCCAAT<br>TGCCTAATGATAACAAGCTTAGTTCTTATATCAAATCAGTTTCCAAT<br>TTCAGCTCAAATTTGAAGTTCATTCAACTCCCTCAAGATGAATCTG<br>TTTTGCAGCTACTCAAAGGGAATATTTTCAGTTCTTTTATTCCTGG<br>TCATAAGCCTGCAGTTAGAGATGCTGTGGCTGAAATTCTCAAGTC<br>AGAATCAGATATCACACTAGCAGGTATTGTCATCGACTTGTTCTGC<br>ACCTCTATGATTGATGTGGCCAATGAACTTGAGCTACCAACCTATG<br>TTTTCTACACCTCTAATGCAGCCTCTCTTGGTCTTCAATTCCATATG<br>CAGAGTCTTAGTGATGAATTTAATATAGATATTACCAATTACAAAA<br>ATAACCCTGAAGCAGAACTTTCTATATCAACATATCTCAATCCATTT<br>CCAGCAAAATGTTTGCCCTCCATAGCCTTAGACAAGGAAGGAGG<br>AGGTTCCACCATGTATCTCGATCTTACGAGAAGGATTCGAGAAAC<br>GAAAGGTATTATGATAAACACATTTGTTGAAATCGAGCCCCATGCG<br>ATCAACTCGCTCTTACGAGACAAGAATATACCGCCTGTATACCCTG<br>TTGGACCCGTGTTGAACCTTAATAATGTTGAAAGTGACAAATTGA<br>GTGAATCTGATAAGAATATTATGAAGTGGCTAGATGATCAATCCCC	MKETKNIELIFIPCPGIGH<br>LVSTVEMAKLLITREKH<br>MSITVLIIQLPNDNKLSS<br>YIKSVSNFSSNLKFIQLP<br>QDESVLQLLKGNIFSSFIP<br>GHKPAVRDAVAEILKSES<br>DITLAGIVIDLFCTSMIDV<br>ANELELPTYVFYTSNAA<br>SLGLQFHMQSLSDEFNID<br>ITNYKNNPEAELSISTYL<br>NPFPAKCLPSIALDKEGG<br>GSTMYLDLTRRIRETKGI<br>MINTFVEIEPHAINSLLR<br>DKNIPPVYPVGPVLNLN<br>NVESDKLSESDKNIMKW<br>LDDQSPASVVFLCFGSG<br>GSFKKDQVKEIAYALEN<br>SGCQFLWSLRQPPEKDA

	TGCATCTGTAGTGTTCCTCTGTTTTGGTAGTGGTGGAAGTTTTAAA AAAGATCAAGTTAAGGAAATAGCCTACGCTCTAGAAAATAGTGGG TGTCAGTTTTTGTGGTCACTGAGGCAACCACCAGAGAAAGATGC AAGGTTTCCAAGTGACTATGAAAATTTTGAAGAAGTCTTGCCAGA AGGGTTCTTGCAAAGAACACAAAGGATTGGAAAGGTGATGGGAT GGGCACCTCAATTGGCAATTTTGTCTCATAAAGCTGTGGGAGGCT TTGTGTCACACTGTGGGTGGAATTCGACTTTGGAAAGCATTTATTT CGGAGTGCCAATGGCTACTTGGCCAATGTACGCAGAGCAACAAG GGAATGCATTTCAATTGGTTAAGGATTTGGGAATGGCAGTCGAGA TTAAGATGGATTACAGGAAGGATCCGAAAGTGATGGGCCAAGAA ATTATAGTGAAAGCAGAGAAGATTGAGAAAGCGATAAGGGAACT TATGGACCCGGAGAATGAAATTTGGATGAAAGTGAAAAATATGAA GGAGAAGGGCAGAGCGGCAACAATGGAAGGTGGCTCTTCTTATA ATTGTATTGGAGGTTTCATCCAGAGTATCATGGAGAATACTCGGTA A	RFPSDYENFEEVLPEGFL QRTQRIGKVMGWAPQL AILSHKAVGGFVSHCGW NSTLESIYFGVPMATWP MYAEQQGNAFQLVKDL GMAVEIKMDYRKDPKV MGQEIIVKAEKIEKAIRE LMDPENEIWMKVKNMK EKGRAATMEGGSSYNCI GGFIQSIMENTR*
LbUGT4	ATGGCAGAAACACCTGTTGTGACTCCCCATATTGCAATATTACCAT CTCCTGGTATGGGTCACTTAATCCCTCTTGTTGAATTCTCTAAAAG ACTCATTCAAAATCACCATTTTTCTGTTACTCTTATTCTTCCTACAG ATGGCCCTGTCTCTAATGCTCAGAAAATTTACCTTAATTCACTCCC TTGTTCCATGGATTATCACCTTCTTCCACCTGTAAACTTCGATGATT TACCACTAGATACTAAAATGGAAACTCGTATTTCCCTTACTGTTAC ACGTTCTCTTCCTTCACTACGTGAAGTCTTTAAAACACTTGTTGA AACCAAGAAAACAGTTGCTCTTGTTGTTGATCTTTTTGGTACTGAT GCATTTGATGTTGCAAATGATTTTAAGGTTTCTCCTTATATTTTTTA CCCTTCTACTGCTATGGCTTTGTCTCTGTTTCTTTACTTGCCTAAGC TTGATGAAACTGTGTCATGTGAGTATACAGACTTGCCTGACCCGG TTCAGATTCCGGGTTGTATTCCTATCCATGGAAAGGATCTTCTTGA CCCGGTTCAGGACAGGAAAAATGAAGCTTACAAATGGGTTCTTC ATCATTCAAAGAGGTATAGAATGGCTGAAGGAATAGTAGCTAACA GCTTTAAAGAATTGGAAGGTGGAGCTATTAAAGCTCTTCAAGAAG AAGAACCCGGTAAACCACCCGTTTACCCGGTTGGACCACTTATCC AGATGGATTCGGGTTCGGGTAGTAAGGCTGACAGGTCAGAGTGT CTGACGTGGCTTGATGAACAGCCACGTGGCTCTGTGTTGTATATTT CGTTCGGGAGTGGTGGGACCCTCTCTCATGAGCAGATGATTGAAC TTGCATCAGGCTTGGAAATGAGTGAACAAAGGTTCTTGTGGGTAA TTAGAACCCCCAATGACAAAATGGCCAGTGCTACTTATTTTAATGT CCAAGACTCAACTAACCCTCTTGATTTCCTCCCAAAAGGGTTCTT GGAAAAGACCAAAGGGTTAGGTTTAGTGGTTCCCAATTGGGCCC CACAGGCTCAAATCCTTGGTCATGGTTCAACTAGCGGGTTCTTAA CTCACTGTGGATGGAACTCGACTTTGGAGAGCGTGGTCCACGGG GTACCATTCATAGCTTGGCCACTCTATGCAGAACAAAAAATGAAT GCGGTTATGTTAAGTGAGGATATAAAAGTGGCACTAAGGCCAAAG GCCAATGAAAATGGAATAGTGGGACGATTAGAAATTGCTAAAGTT GTGAAAGGACTAATGGAAGGTGAAGAAGGAAAAGTAGTACGCA	MAETPVVTPHIAILPSPG MGHLIPLVEFSKRLIQNH HFSVTLILPTDGPVSNAQ KIYLNSLPCSMDYHLLPP VNFDDLPLDTKMETRIS LTVTRSLPSLREVFKTLV ETKKTVALVVDLFGTDA FDVANDFKVSPYIFYPST AMALSLFLYLPKLDETV SCEYTDLPDPVQIPGCIPI HGKDLLDPVQDRKNEA YKWVLHHSKRYRMAEG IVANSFKELEGGAIKALQ EEEPGKPPVYPVGPLIQM DSGSGSKADRSECLTWL DEQPRGSVLYISFGSGGT LSHEQMIELASGLEMSE QRFLWVIRTPNDKMASA TYFNVQDSTNPLDFLPK GFLEKTKGLGLVVPNWA PQAQILGHGSTSGFLTHC GWNSTLESVVHGVPFIA WPLYAEQKMNAVMLSE DIKVALRPKANENGIVG RLEIAKVVKGLMEGEEG KVVRSRMRDLKDAAAK VLSEDGSSTKALAELAT KLKKKVSNN*

	GTAGAATGAGAGATCTTAAAGATGCAGCAGCCAAAGTGCTGAGT<br>GAAGATGGTTCTTCTACAAAAGCACTAGCTGAATTGGCTACTAAA<br>TTGAAGAAAAAAGTGTCAAATAATTGA	
LbUGT5	ATGAGCAAATTAGAGCTGGTGTTTGTGCCTGCCCCAGCCGTTGGT<br>CACCTTGTTTCGACATGCAAGTTTGCAGAAAAGTTGTTTGATAGA<br>GATGAAAGGTTATGTATAACAATTCTGATCATAAGGCCACCCCCAC<br>CATGGGATGTCAGCATAGATGCCTACATCAAACGAAGCTCCTCTG<br>CTCCGGAGGGCAGACGAATTCGATATATCACGCTTCCTCAAGCCG<br>AACCGCCACCTGCAGAAGAACTAGCCAAGTCCATTGAGAACTATT<br>TCTCTCTATTAATAGCAAATTATCGACCCATCGTCAAGGAAGCAAT<br>TATCAGCAACAAGTGGCCTGATTCGAACCCGAAAATTATTGGCCT<br>AGTCATTGATATGTTTTGTAGTGCAATGATTGATGTAGCAAAAGAA<br>ATTGACATTCCTTCCTATCTTTTCTTCACTTCTGGTGCAGGTTTTCT<br>TGGTTTCTTGTTATACCTGTCCGTTTGGCATGAAAAAGTTGGGAG<br>AGAATTTAACAGATCTGATGCTCATTTAAAGATAGCATCCTATGCT<br>CATCCAGTACCCTCAAAAGTATTGCCTACTTTTGCATTCAGCAAGG<br>AAGGTTACGACTCATTTCGCGAACATGGTGTCAGGTTCAAGGAAA<br>CTAAAGGAATTCTCATCAACACAGTTGCGGAATTCGAAAGCCATG<br>CTGTAAATAGCTTAGCATCTGATCCTGAATTACCTCCAGTTTACAC<br>GGTAGGATTTCTTAATGACCTTGAGGGACAAAAAGGTAAGGGAA<br>ATTCCAAGACTGAAGATGAAGAAATCATGAAATGGCTAGACAAG<br>CAGCCACCTTCATCTGTTCTTTTCTGTGCTTTGGAAGCGCGGGTA<br>TTTTTGAGCCACCACAGCTAATCGAGATGGCAATTGCACTCGAAC<br>GATGTGGGGTTAGATTCTTGTGGTCAATACGCCGTCCTGTGGATGC<br>TGAACCTTCAAAATTTGAGGAGATATTCCCAGAAGGGTTCTTGGA<br>GAGGACAAAAAATAGAGGAATAGTATGTGGATGGGCACCCCAAG<br>TCGATATCTTGGCTCACGGGGCAACTGGAGCATTCGTATCTCATTG<br>TGGGTGGAATTCGATTGTAGAGAGTGTATGGCATGGAGTTCCAAT<br>TGTGACATGGCCCCTTTATGCAGAACAACATATCAATGCGTTTCAA<br>TTGGTGAATGATCTTGAGATAGCAGTTGAGTTGACATTGGATTACA<br>GGATGCTCGAAAGTGATCATAGTGAAAGTGTGAAGGCACAGGAG<br>ATGGAGAAAGCTATAAGATGCATAATGGACAGCGAAAATCCCATG<br>AGGAAGAAAGTCAAATATATGGAAGAGATTTGCAGGAAGGCGCT<br>GATGGAGGGTGGATCTTCTTTCAATTCTATGGGACGATTTGTTGAA<br>ACTATTCTTGATTCCTAG	MSKLELVFVPAPAVGHL<br>VSTCKFAEKLFDRDERL<br>CITILIIRPPPPWDVSIDAY<br>IKRSSSAPEGRRIRYITLP<br>QAEPPPAEELAKSIENYF<br>SLLIANYRPIVKEAIISNK<br>WPDSNPKIIGLVIDMFCS<br>AMIDVAKEIDIPSYLFFTS<br>GAGFLGFLLYLSVWHEK<br>VGREFNRSDAHLKIASY<br>AHPVPSKVLPTFAFSKEG<br>YDSFREHGVRFKETKGI<br>LINTVAEFESHAVNSLAS<br>DPELPPVYTVGFLNDLE<br>GQKGKGNSKTEDEEIMK<br>WLDKQPPSSVLFLCFGS<br>AGIFEPPQLIEMAIALER<br>CGVRFLWSIRRPVDAEPS<br>KFEEIFPEGFLERTKNRGI<br>VCGWAPQVDILAHGATG<br>AFVSHCGWNSIVESVWH<br>GVPIVTWPLYAEQHINAF<br>QLVNDLEIAVELTLDYR<br>MLESDHSESVKAQEME<br>KAIRCIMDSENPMRKKV<br>KYMEEICRKALMEGGSS<br>FNSMGRFVETILDS*
