# Supplementary material for: Functional and structural dissection of glycosyltransferases underlying the glycodiversity of wolfberry-derived bioactive ingredients lycibarbarspermidines
Source: Nat Commun. 2024 May 30;15:4588. doi: 10.1038/s41467-024-49010-9 (PMC11139883; doi:10.1038/s41467-024-49010-9)
Supplement: Supplementary file 3 — Description of Additional Supplementary Files [file 41467_2024_49010_MOESM3_ESM.pdf]

Description of Additional Supplementary Files

Supplementary Data 1: The initial and final configurations of LbUGT1-4 complexed with substrate **1**, **2** or **8** for molecular dynamics trajectories.
