# Supplementary material for: Functional and structural dissection of glycosyltransferases underlying the glycodiversity of wolfberry-derived bioactive ingredients lycibarbarspermidines
Source: Nat Commun. 2024 May 30;15:4588. doi: 10.1038/s41467-024-49010-9 (PMC11139883; doi:10.1038/s41467-024-49010-9)
Supplement: Supplementary file 5 — Reporting Summary [file 41467_2024_49010_MOESM5_ESM.pdf]

nature portfolio

Corresponding author(s): Ikuro Abe, Hao Gao, and Dan Hu

Last updated by author(s): Apr 22, 2024

Reporting Summary

Nature Portfolio wishes to improve the reproducibility of the work that we publish. This form provides structure for consistency and transparency in reporting. For further information on Nature Portfolio policies, see our Editorial Policies and the Editorial Policy Checklist.

Statistics

For all statistical analyses, confirm that the following items are present in the figure legend, table legend, main text, or Methods section.

n/a	Confirmed	
☐	☒	The exact sample size (n) for each experimental group/condition, given as a discrete number and unit of measurement
☐	☒	A statement on whether measurements were taken from distinct samples or whether the same sample was measured repeatedly
☐	☒	The statistical test(s) used AND whether they are one- or two-sided<br>*Only common tests should be described solely by name; describe more complex techniques in the Methods section.*
☒	☐	A description of all covariates tested
☒	☐	A description of any assumptions or corrections, such as tests of normality and adjustment for multiple comparisons
☐	☒	A full description of the statistical parameters including central tendency (e.g. means) or other basic estimates (e.g. regression coefficient) AND variation (e.g. standard deviation) or associated estimates of uncertainty (e.g. confidence intervals)
☐	☒	For null hypothesis testing, the test statistic (e.g. F, t, r) with confidence intervals, effect sizes, degrees of freedom and P value noted<br>*Give P values as exact values whenever suitable.*
☒	☐	For Bayesian analysis, information on the choice of priors and Markov chain Monte Carlo settings
☒	☐	For hierarchical and complex designs, identification of the appropriate level for tests and full reporting of outcomes
☒	☐	Estimates of effect sizes (e.g. Cohen's d, Pearson's r), indicating how they were calculated

Our web collection on statistics for biologists contains articles on many of the points above.

Software and code

Policy information about availability of computer code

Data collection	LC data: Dionex UltiMate 3000 HPLC system (Thermo Scientific). MS data: amaZon SL ion trap mass spectrometer (Bruker). HRESIMS data: Waters Synapt G2 TOF mass spectrometer (Waters). NMR data: Bruker AV 400/600 spectrometers (Bruker). X-ray data: the beam-line BL19U1 at the Shanghai Synchrotron Radiation Facility. Computational modeling data: Autodock 4.0 for molecular docking. AMBER22 (https://ambermd.org/AmberMD.php) for MD calculations.
Data analysis	LC data: Chromatography Data System v7.1 (Thermo Scientific). MS data: HyStar v3.2 (Bruker). HRESIMS data: Xcalibur v4.2 (Thermo Scientific). NMR data: MestReNova v9.1.0. Kinetic parameter: GraphPad Prism v8.0. X-ray data: the xia2 (https://github.com/xia2/xia2), PHENIX v1.16_3549-000, Coot v0.8.9, Autodock 4.0 and Pymol v0.99 softwares were used for data processing and analysis. MD simulations were performed with AMBER22 (https://ambermd.org/AmberMD.php).

For manuscripts utilizing custom algorithms or software that are central to the research but not yet described in published literature, software must be made available to editors and reviewers. We strongly encourage code deposition in a community repository (e.g. GitHub). See the Nature Portfolio guidelines for submitting code & software for further information.

Data

Policy information about availability of data

All manuscripts must include a data availability statement. This statement should provide the following information, where applicable:

- Accession codes, unique identifiers, or web links for publicly available datasets
- A description of any restrictions on data availability
- For clinical datasets or third party data, please ensure that the statement adheres to our policy

All relevant data are available within the article and its Supplementary Information and from the corresponding authors on request. The structures of LbUGT1 and LbUGT3 have been deposited in the Protein Data Bank under codes 8WP5 [https://www.rcsb.org/structure/unreleased/8WP5] and 8W53 [https://www.rcsb.org/structure/unreleased/8W53], respectively. The GenBank accession numbers for nucleotide sequences of LbUGT1, LbUGT2, LbUGT3, LbUGT4 and LbUGT5 are BankIt2754211 LbUGT1 OR684509, BankIt2754211 LbUGT2 OR684510, BankIt2754211 LbUGT3 OR684511, BankIt2754211 LbUGT4 OR684512 and BankIt2754211 LbUGT5 OR684513, respectively. The primers and predicted DNA sequences are given in Supplementary Table 10 and 11. Source data are provided with this paper.

Research involving human participants, their data, or biological material

Policy information about studies with human participants or human data. See also policy information about sex, gender (identity/presentation), and sexual orientation and race, ethnicity and racism.

Reporting on sex and gender	Not involved in this study
Reporting on race, ethnicity, or other socially relevant groupings	Not involved in this study
Population characteristics	Not involved in this study
Recruitment	Not involved in this study
Ethics oversight	Not involved in this study

Note that full information on the approval of the study protocol must also be provided in the manuscript.

Field-specific reporting

Please select the one below that is the best fit for your research. If you are not sure, read the appropriate sections before making your selection.

[x] Life sciences [ ] Behavioural & social sciences [ ] Ecological, evolutionary & environmental sciences

For a reference copy of the document with all sections, see nature.com/documents/nr-reporting-summary-flat.pdf

Life sciences study design

All studies must disclose on these points even when the disclosure is negative.

Sample size	Biochemical and enzymatic assays were completed using wild type or mutants of LbUGT1-5 and three independent biological samples were tested to obtain the mean values. The sample size is sufficient to delineate the mutational effects of the proteins.
Data exclusions	No data excluded
Replication	All the experiments were performed by three biologically independent samples, and they show good reproducibility.
Randomization	The assays performed in this study require a rational approach for activity comparison. Therefore, randomization is not applicable to our experimental set up.
Blinding	Blinding is not applicable in our study because it does not involve subjects receiving different treatments. All experiments were conducted by analyzing data derived from different biological replicates.

Reporting for specific materials, systems and methods

We require information from authors about some types of materials, experimental systems and methods used in many studies. Here, indicate whether each material, system or method listed is relevant to your study. If you are not sure if a list item applies to your research, read the appropriate section before selecting a response.

Materials & experimental systems

n/a	Involved in the study
☒	☐ Antibodies
☒	☐ Eukaryotic cell lines
☒	☐ Palaeontology and archaeology
☒	☐ Animals and other organisms
☒	☐ Clinical data
☒	☐ Dual use research of concern
☒	☐ Plants

Methods

n/a	Involved in the study
☒	☐ ChIP-seq
☒	☐ Flow cytometry
☒	☐ MRI-based neuroimaging

Plants

Seed stocks

Report on the source of all seed stocks or other plant material used. If applicable, state the seed stock centre and catalogue number. If plant specimens were collected from the field, describe the collection location, date and sampling procedures.

Novel plant genotypes

Describe the methods by which all novel plant genotypes were produced. This includes those generated by transgenic approaches, gene editing, chemical/radiation-based mutagenesis and hybridization. For transgenic lines, describe the transformation method, the number of independent lines analyzed and the generation upon which experiments were performed. For gene-edited lines, describe the editor used, the endogenous sequence targeted for editing, the targeting guide RNA sequence (if applicable) and how the editor was applied.

Authentication

Describe any authentication procedures for each seed stock used or novel genotype generated. Describe any experiments used to assess the effect of a mutation and, where applicable, how potential secondary effects (e.g. second site T-DNA insertions, mosiacism, off-target gene editing) were examined.
